# Exploration of Aromatic Hydrazides as Inhibitors of Human Carbonic Anhydrases

**DOI:** 10.1002/ardp.202400963

**Published:** 2025-04-01

**Authors:** German Benito Menendez, Simone Giovannuzzi, Alessandro Bonardi, Alessio Nocentini, Paola Gratteri, Claudiu T. Supuran

**Affiliations:** ^1^ NEUROFARBA Department, Pharmaceutical and Nutraceutical Section University of Florence Florence Italy; ^2^ NEUROFARBA Department, Pharmaceutical and Nutraceutical Section, Laboratory of Molecular Modeling Cheminformatics & QSAR University of Florence Florence Italy

**Keywords:** carbonic anhydrase, enzyme inhibition, hydrazide, *in silico*

## Abstract

A large set of hydrazide‐based derivatives were explored as inhibitors of the human (h) carbonic anhydrase (CA) isoforms I, II, IV, IX, and XII. A wide series of compounds were synthesized and then assessed for their CA inhibitory activity using a CO_2_ hydrase stopped‐flow assay. Generally, these inhibitors demonstrated micromolar activity against the evaluated hCAs. Specifically, some derivatives bearing a ureido‐linker exhibited the highest inhibitory potency, showing inhibition constants (*K*_I_s) in the low‐micromolar range against hCAs IV, XI, and XII. Moreover, two of them were detected as submicromolar inhibitors of isoform IV (*K*_I_s: 0.8–0.96 µM). Molecular modeling was carried out to investigate the binding mode of the most selective and potent compounds and reinforce the experimental results. The latter suggests that hydrazide compounds act as zinc binders, being bidentate ligands, and can be developed as an alternative to classic CA inhibitors.

## Graphical abstract

Hydrazide‐based derivatives were evaluated as inhibitors of human carbonic anhydrase (hCA) isoforms I, II, IV, IX, and XII. Several derivatives, particularly those with ureido‐linkers, exhibited strong inhibitory activity with inhibition constants in the low micromolar range. Molecular modeling confirmed that these inhibitors act as bidentate zinc binders, elucidating their binding modes and selectivity.

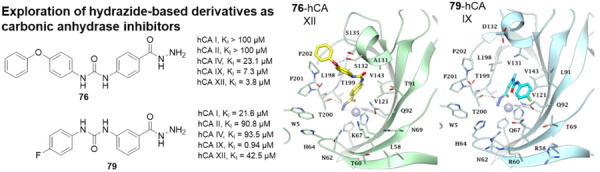

## 1 Introduction

**Figure 1:**
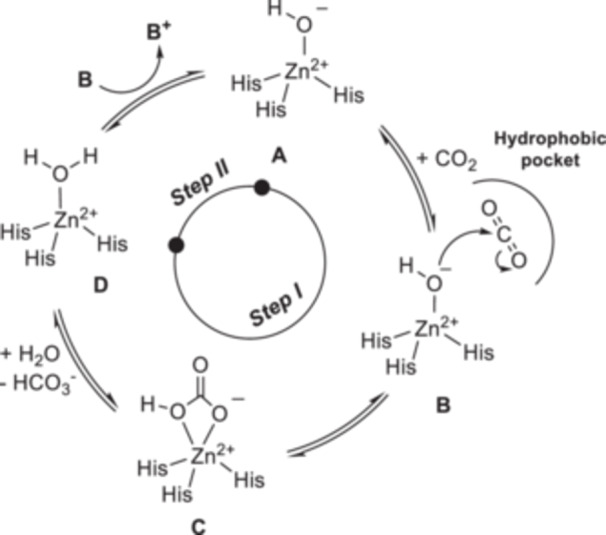
Schematic representation of the hCA catalytic mechanism.

Carbonic anhydrases (CAs; EC 4.2.1.1) are a family of ubiquitous *meta*lloenzymes found in most eukaryotic and prokaryotic organisms. CAs are encoded by eight evolutionarily unrelated gene families [1, 2, 3, 4, 5, 6, 7, 8, 9, 10, 11, 12, 13, 14, 15, 16]. Fifteen α‐class isozymes have been identified in humans (h), differing in molecular features, cellular localization, organ and tissue distribution and expression, and kinetic properties [17, 18]. CAs play a crucial role in the reversible hydration of carbon dioxide (CO_2_) to produce bicarbonate (HCO_3_^−^) and proton, a reaction of significance at the physiological level as it serves as the primary buffer system and is involved in vital *meta*bolic transformations. The reversible carbon hydration reaction catalyzed by hCAs occurs through a two‐step catalytic mechanism (Figure 1): primarily, the production of bicarbonate and proton (step I) and regeneration of the enzyme active form through to the proton shuttle (step II) [1, 2].

The reaction catalyzed by CA mentioned above is crucial for various physiological processes, including acid–base homeostasis, respiration, and ion transport. Initially, inhibition of these isozymes was primarily employed in the production of diuretic drugs, antiglaucoma agents, antiepileptic drugs, and in managing altitude sickness [1]. Furthermore, in recent decades, abnormal levels or activities of these enzymes have been linked to multifactorial human diseases, such as various types of hypoxic cancers, Alzheimer's disease, mnemonic dysfunction, and neuropathic pain [19, 20, 21]. Consequently, carbonic anhydrases have emerged as intriguing targets for the design of numerous inhibitors or activators.

**Figure 2:**
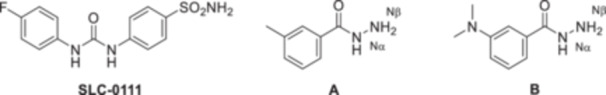
Structure of SLC‐0111 and compounds cocrystallized with hCA II, i.e., 3‐methylbenzohydrazide **A** and 3‐(dimethylamino)benzohydrazide **B**.

Among all human CAs, isozymes IX and XII are well‐established as anticancer drug targets, particularly for treating hypoxic tumors where these isozymes are aberrantly expressed. Their role primarily involves maintaining intracellular pH values within viable ranges as part of dynamic survival mechanisms in cancer cells. Recent studies have associated their inhibition with significant growth inhibition of both primary tumors and *meta*stases. Various classes of compounds exhibiting good selectivity for tumor‐associated CAs have been reported. Inhibition of CA IX/XII reverses tumor acidification by impeding the catalytic activity of the enzyme, thereby inhibiting the generation of H^+^ ions and ultimately restraining cancer cell growth [22, 23]. The most notable and well‐known selective inhibitor of hCA IX and XII is SLC‐0111 (Figure 2), currently undergoing phase Ib/II clinical trials in North America for treating advanced hypoxic tumors.

Given the role of specific carbonic anhydrase isoforms in various pathologies, there is a growing demand for novel isoform‐selective inhibitors. Consequently, researchers are increasingly focusing on exploring new carbonic anhydrase modulators [24, 25, 26]. In this context, we present the design, synthesis, and kinetic evaluation of a new series of aromatic hydrazides as potential carbonic anhydrase inhibitors.

## 2 Results and Discussions

### 2.1 Chemistry

**Figure 3:**
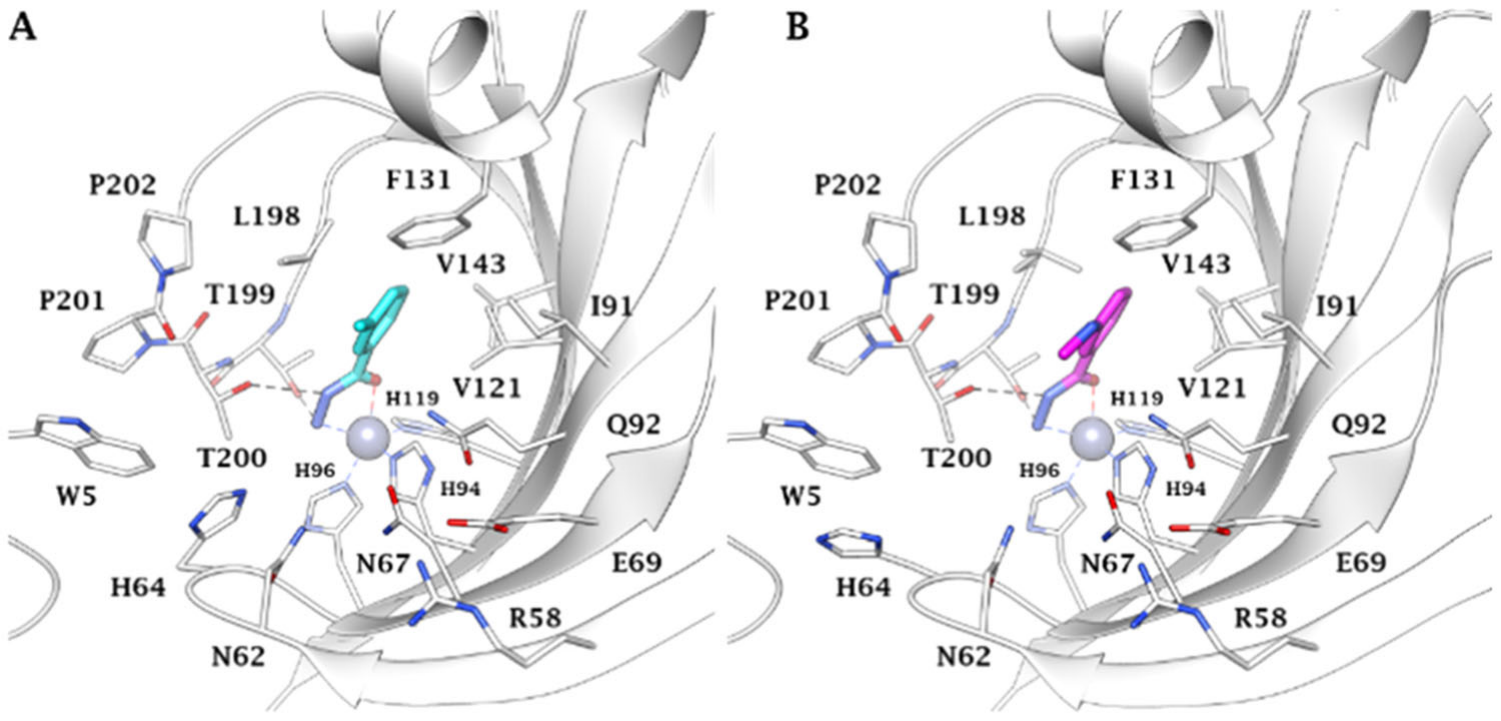
X‐ray solved structures of (A) 3‐methylbenzohydrazide (magenta, PDB: 6RM1), and (B) 3‐(dimethylamino)benzohydrazide (green, PDB: 6RMP) in complex with hCA II [29]. The zinc ion is represented as a gray sphere coordinated with H94, H96, and H119, whereas the H‐bond interactions are depicted as black dashed lines.

*Design and chemistry*. Based on preceding studies reported by our group [27, 28], in 2020, Glöckner et al. cocrystallized hCA II with hydrazide‐based compounds unveiling their mechanism of action as zinc binder compounds. Specifically, the authors evaluated a 96‐entry fragment library by a crystallographic screening with several target proteins, among which is hCA II. The screening achieved the cocrystallization of hCA II with 3‐methylbenzohydrazide (Figure 2A) and 3‐(dimethylamino)benzohydrazide (Figure 2B). The hydrazide group coordinates the Zn(II) cofactor with the Nβ and the carbonyl O‐atom of the hydrazide function, which results in a penta‐coordinated cofactor. Moreover, Nα and Nβ atoms are involved in a hydrogen bond with T200 and T199, respectively, while the phenyl ring generates a π‐interaction with L198 (Figure 3).

The findings led us to extend the series of aromatic hydrazides as CA inhibitors to work out a thorough structure–activity relationship against a panel of hCAs. A computational study was used to elucidate the inhibition profiles against the different isozymes.

**Scheme 1:**
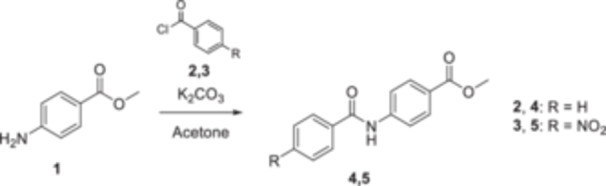
Synthesis of the amido‐based intermediates 4 and 5.

**Scheme 2:**
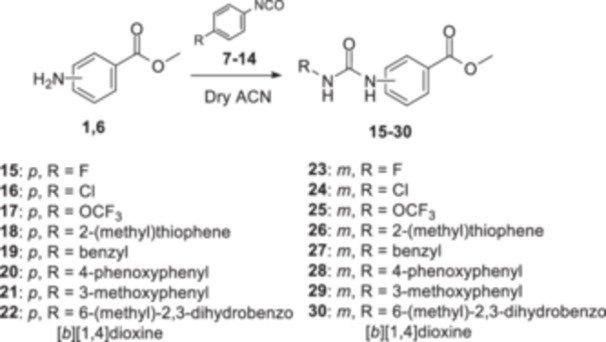
Synthesis of the urea‐based intermediates **15–30**.

**Scheme 3:**
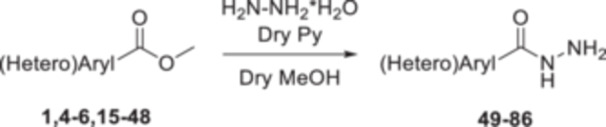
Synthesis of the aromatic hydrazide‐based derivatives **49–86**.

Initially, the core of each compound was synthesized starting with compounds **4** and **5**, which were achieved using the commercially available methyl 4‐aminobenzoate **1**, by a reaction with acyl chlorides **2** and **3** in acetone with potassium carbonate as a base (Scheme 1). Therefore, a set of urea‐based methyl ester compounds (**15**–**30**) was synthesized by reacting methyl 4‐ or 3‐aminobenzoate **1** or **6** with isocyanates **7**–**14** in anhydrous acetonitrile at room temperature (Scheme 2). Finally, all methyl esters **4**, **5**, **15**–**48** were converted into the corresponding hydrazide‐based compounds (**49**–**86**) by aminolysis promoted by hydrazine monohydrate in the presence of pyridine in refluxing anhydrous methanol (Scheme 3). Among them, derivatives **71** and **72** have already been synthesized and characterized as intermediates for the synthesis of RAGE inhibitors [30].

### 2.2 Carbonic Anhydrase Inhibition Assay

The CA inhibition profile of compounds **49**–**86** was evaluated against five physiologically relevant isoforms, hCA I, II, IV, IX, and XII, by a CO_2_ hydrase, stopped‐flow assay [31], using acetazolamide (AAZ) as a reference drug (Table 1).

**Table 1:** Inhibition data of human isoforms hCA I, II, IV, IX, and XII, by a CO_2_ hydrase, stopped‐flow assay, using acetazolamide (AAZ) as reference drug.

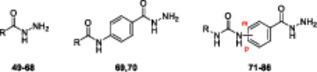							
Cmp	*m/p*	R	*K*_I_ (μM)^a^				
			CA I	CA II	CA IV	CA IX	CA XII
**49**	—	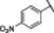	> 100	> 100	> 100	> 100	> 100
**50**	—	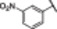	> 100	> 100	> 100	> 100	> 100
**51**	—	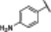	> 100	> 100	> 100	> 100	> 100
**52**	—	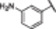	> 100	77.1	> 100	> 100	> 100
**53**	—	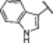	> 100	> 100	> 100	> 100	> 100
**54**	—	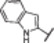	> 100	> 100	> 100	> 100	> 100
**55**	—	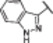	> 100	79.2	> 100	> 100	> 100
**56**	—	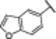	> 100	> 100	> 100	> 100	> 100
**57**	—	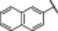	> 100	> 100	> 100	> 100	> 100
**58**	—	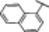	> 100	> 100	> 100	> 100	> 100
**59**	—	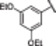	> 100	> 100	> 100	> 100	> 100
**60**	—	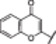	> 100	> 100	> 100	> 100	> 100
**61**	—	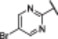	> 100	> 100	> 100	> 100	> 100
**62**	—	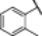	> 100	> 100	> 100	> 100	> 100
**63**	—	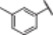	> 100	> 100	> 100	> 100	> 100
**64**	—	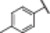	> 100	> 100	> 100	> 100	> 100
**65**	—	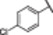	> 100	93.5	> 100	> 100	> 100
**66**	—	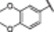	> 100	> 100	> 100	> 100	> 100
**67**	—	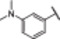	> 100	38.8	> 100	> 100	> 100
**68**	—	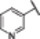	> 100	> 100	> 100	> 100	> 100
**69**	—	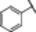	> 100	71.5	> 100	68.4	45.4
**70**	—	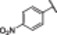	> 100	69.5	79.5	22.0	36.6
**71**	p	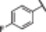	88.5	88.6	> 100	2.3	20.6
**72**	p	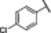	> 100	56.8	> 100	29.2	61.2
**73**	p	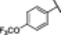	> 100	88.2	> 100	36.7	64.7
**74**	p	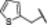	> 100	> 100	6.1	91.7	39.8
**75**	p	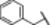	> 100	> 100	34.2	39.6	9.2
**76**	p	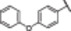	> 100	> 100	23.1	7.3	3.8
**77**	p	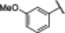	> 100	96.6	29.7	79.3	6.5
**78**	p	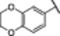	> 100	> 100	2.2	13.9	5.0
**79**	m	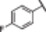	21.6	90.8	93.5	0.94	42.5
**80**	m	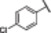	> 100	62.8	> 100	18.5	23.6
**81**	m	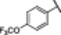	> 100	64.8	61.8	23.6	67.0
**82**	m	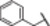	> 100	> 100	0.96	34.4	28.3
**83**	m	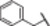	75.6	89.0	48.4	9.3	7.7
**84**	m	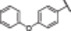	> 100	87.1	24.6	20.9	8.5
**85**	m	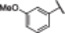	> 100	> 100	2.0	80.6	6.9
**86**	m	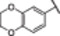	> 100	17.2	0.80	9.6	12.0
AAZ	—	—	0.25	0.012	0.074	0.025	0.0057

^a^ Mean from three different assays, by a stopped‐flow technique (errors were in the range of ± 5%–10% of the reported values).

The following structure–activity relationship (SAR) can be gathered from the inhibition data reported in Table 1:

i. The cytosolic hCA I was the less effectively inhibited isoform of the panel here tested. All the derivatives **49**–**68** resulted to be inactive toward this isoform, *K*_I_ > 100 μM. Nevertheless, three compounds, **71**, **79,** and **83**, showed a poor inhibition profile, observing *K*_I_s of 88.5, 21.6, and 75.6 μM, respectively.
ii. The cytosolic hCA II was more inhibited than the previous one. Among compounds **49**–**68**, compound **52**, bearing the amino group in the *meta* position, showed regioselective inhibition with a *K*_I_ of 77.1 μM, as its *para* derivative was inactive. Additionally, the indazole‐based compound **55**, *K*_I_ of 79.2 μM, and compound **67**, *K*_I_ of 38.8 μM, with a dimethylamino portion in *meta* position, exhibited low micromolar inhibition, making it one of the most potent hCA II inhibitors. Meanwhile, the amido‐based compounds **69** and **70** pointed out high micromolar inhibition, with *K*_I_s of 71.5 and 69.5 μM, respectively. Regarding the ureido‐based compounds, both *para* and *meta* 4‐fluorophenyl ureido‐based compounds **71** and **79** were found to be high micromolar inhibitors, showing *K*_I_s of 88.6 and 90.8 μM, respectively, while a slight inhibition improvement was observed for the *para* and *meta* 4‐chlorophenyl ureido‐based compounds **72** and **80**, resulting also in micromolar inhibition, *K*_I_s of 56.8 and 62.8 μM, respectively. Moreover, also the *para* and *meta* 4‐(trifluoromethoxy)phenyl ureido compounds **73** (*K*_I_ of 88.2 μM) and **81** (*K*_I_ of 64.8 μM) were found to be medium micromolar inhibitors, as well as *meta* 4‐phenoxyphenyl ureido‐based derivative **84**, which showed *K*_I_ of 87.1 μM. On the other side, the *meta* benzyl ureido‐based compound **83** exhibited high micromolar inhibition, *K*_I_ of 89.0 μM, while its *para* isomer **75** was inactive. A similar behavior was detected also for isomers **77** and **85**, having *K*_I_s of 96.6 μM and over 100 μM, respectively. Finally, the most potent inhibitor against hCA II was the *meta* 2,3‐dihydrobenzo[*b*][1,4]dioxin‐6‐yl ureido‐based compound **86**, acting as a low micromolar inhibitor with a *K*_I_ value of 17.2 μM.
iii. In the context of the hCA IV, compounds **49**–**68** exhibited inactivity, *K*_I_s > 100 μM. Conversely, the majority of the remained compounds displayed inhibitory effects on this isoform, resulting in a heterogeneous distribution of inhibition. Notably, compound **70**, bearing a 4‐nitrobenzamide tail, emerged as a high micromolar inhibitor, *K*_I_ of 79.5 μM, while its analog, compound **69**, remained inactive. Within the realm of halogenated derivatives, only compound **79**, featuring a *meta* 4‐fluorophenyl ureido moiety, demonstrated significant inhibition of this isoform with a high micromolar inhibition value, *K*_I_ of 93.5 μM, as well as compound **81**, bearing a 4‐(trifluoromethoxy)phenyl ureido group, which exhibited a *K*_I_ value of 61.8 μM. On the other side, its *para* isomer, compound **73**, showed no activity against this membrane‐bound isoform. Surprisingly, compounds **74** and **82**, incorporating *para* and *meta* furan‐2‐ylmethyl ureido moieties, respectively, emerged as among the most potent hCA IV inhibitors, boasting low micromolar *K*_I_ values, 6.1, and 0.96 μM, respectively. Compounds **75** and **83**, featuring *para* and *meta* benzyl ureido groups, respectively, displayed a poor inhibition if compared to the previous two, *K*_I_s of 34.2 and 48.4 μM, respectively. Moreover, introducing a 4‐phenoxyphenyl group as the R substituent led to a slight increase in inhibitory potency, as evidenced by compounds **76** and **84**, *K*_I_s of 23.1 and 24.6 μM, respectively. Notably, a significant stereoisomer effect was observed when substituting the R group with a 3‐methoxyphenyl moiety, with the *para* derivative **77**, *K*_I_ of 29.7 μM, being 14‐fold less potent than its *meta*‐analog **85**, *K*_I_ of 2.0 μM, showcasing a low micromolar inhibition. Consistent with findings for hCA II, the most potent inhibitors for this isoform were compounds **78** and **86**, bearing *para* and *meta* 2,3‐dihydrobenzo[*b*][1,4]dioxin‐6‐yl ureido groups, respectively, both demonstrating a low micromolar or submicromolar inhibition, *K*_I_s of 2.2 μM and 0.8 μM, respectively.
iv. The transmembrane tumor‐associated hCA IX isoform exhibited significant inhibition by select compounds. Notably, none of the untailed compounds **49**–**68** demonstrated inhibitory activity against this isoform, consistent with findings for other evaluated isozymes. Among the amido‐linked compounds, compound **69**, a benzyl derivative, displayed medium micromolar inhibition with a *K*_I_ of 68.4 μM, as well as compound **70**, a 4‐nitrobenzamide derivative, that achieved the same range of inhibition, *K*_I_ of 22.0 μM. Surprisingly, the *para* and *meta* 4‐fluorophenyl ureido‐based compounds **71** and **79** emerged as the most potent inhibitors against this isoform, *K*_I_s of 2.3 and 0.94 μM, respectively, also exhibiting notable selectivity. However, replacing fluorine with chlorine led to inhibition impairment, as evidenced by compounds **72**, *K*_I_ of 29.2 μM, and **80**, *K*_I_ of 18.5 μM. Similarly, the *para* and *meta* 4‐(trifluoromethoxy)phenyl ureido compounds **73** and **81** demonstrated medium‐to‐low micromolar inhibition, *K*_I_s of 29.2 and 18.5 μM, respectively). Differently, *para* and *meta* furan‐2‐ylmethyl ureido‐based compounds **74** and **82** exhibited poor inhibition compared with other isoforms, with high‐to‐medium micromolar inhibition values of 91.7 and 34.4 μM, respectively. Interestingly, inhibition values for benzyl ureido‐based compounds varied significantly, with the *para* isomer **75**, *K*_I_ of 39.6 μM, being fourfold less potent than its *meta* analog **83**, *K*_I_ of 9.3 μM. Additionally, 4‐phenoxyphenyl ureido‐based compounds displayed upsidedown stereoisomer effects, with the *para* isomer **76**, *K*_I_ of 7.3 μM, exhibiting twofold greater potency than the *meta* isomer **84**, *K*_I_ of 20.9 μM. Substituting the R group with a 3‐methoxyphenyl moiety resulted in inhibition impairment, as observed with compounds **77** and **85**, which displayed medium‐to‐high micromolar inhibition, *K*_I_s of 79.3 and 80.6 μM, respectively. Consistent with previous isoforms, *para* and *meta* 2,3‐dihydrobenzo[*b*][1,4]dioxin‐6‐yl ureido‐based compounds **78** and **86** demonstrated low micromolar inhibition, *K*_I_s of 13.9 and 9.6 μM, respectively.
v. The tumor‐associated hCA XII isoform exhibited promising results, emerging as one of the most inhibited isoforms among the tested CAs. Notably, as visible for most of the other isozymes, compounds **49**–**68** showed no activity against this isoform, *K*_I_s > 100 μM. Conversely, all R group‐tailed compounds demonstrated some level of inhibition, with *K*_I_ values ranging from 3.8 to 67.0 μM. Amido‐based compounds **69** and **70** displayed medium micromolar inhibition, *K_I_* s of 45.4 and 36.6 μM, respectively. Notably, a significant inhibition decrease compared with hCA IX isoform was observed for *para* and *meta* 4‐fluorophenyl ureido‐based compounds **71** and **79**, *K*_I_s of 20.6 and 42.5 μM, respectively. Remarkably, the *meta* isomer exhibited a substantial impairment, being 45‐fold less potent against this tumor‐related isoform compared with the former one. This trend persisted with 4‐chlorophenyl ureido‐based compounds **72**, *K*_I_ of 61.2 μM, and **80**, *K*_I_ of 23.6 μM, and 4‐(trifluoromethoxy)phenyl ureido compounds **73**, *K*_I_ of 64.7 μM, and **81**, *K*_I_ of 67.0 μM, which were about twofold less potent. However, the remaining compounds exhibited greater potency against this tumor‐related isoform. In fact, *para* and *meta* furan‐2‐ylmethyl ureido‐based compounds **74** and **82** displayed medium micromolar inhibition, *K*_I_s of 39.8 and 28.3 μM, respectively, while *para* and *meta* benzyl ureido‐based compounds **75**, *K*_I_ of 9.2 μM, and 83, *K*_I_ of 7.7 μM, and *para* and *meta* 4‐phenoxyphenyl ureido‐based compounds **76**, *K*_I_ of 3.8 μM, and 84, *K*_I_ of 8.5 μM, exhibited very low micromolar inhibition. The *para* isomer **76** proves to be the most potent inhibitor against this discussed tumor‐related isoform. Additionally, *para* and *meta* 3‐methoxyphenyl ureido‐based compounds **77** and **85** also displayed very low micromolar inhibition, *K*_I_s of 6.5 and 6.9 μM, respectively, as well as compounds **78** and **86,** which exhibited low micromolar inhibition, *K*_I_s of 6.9 and 12.0 μM, respectively.

### 2.3 In Silico Studies

**Figure 4:**
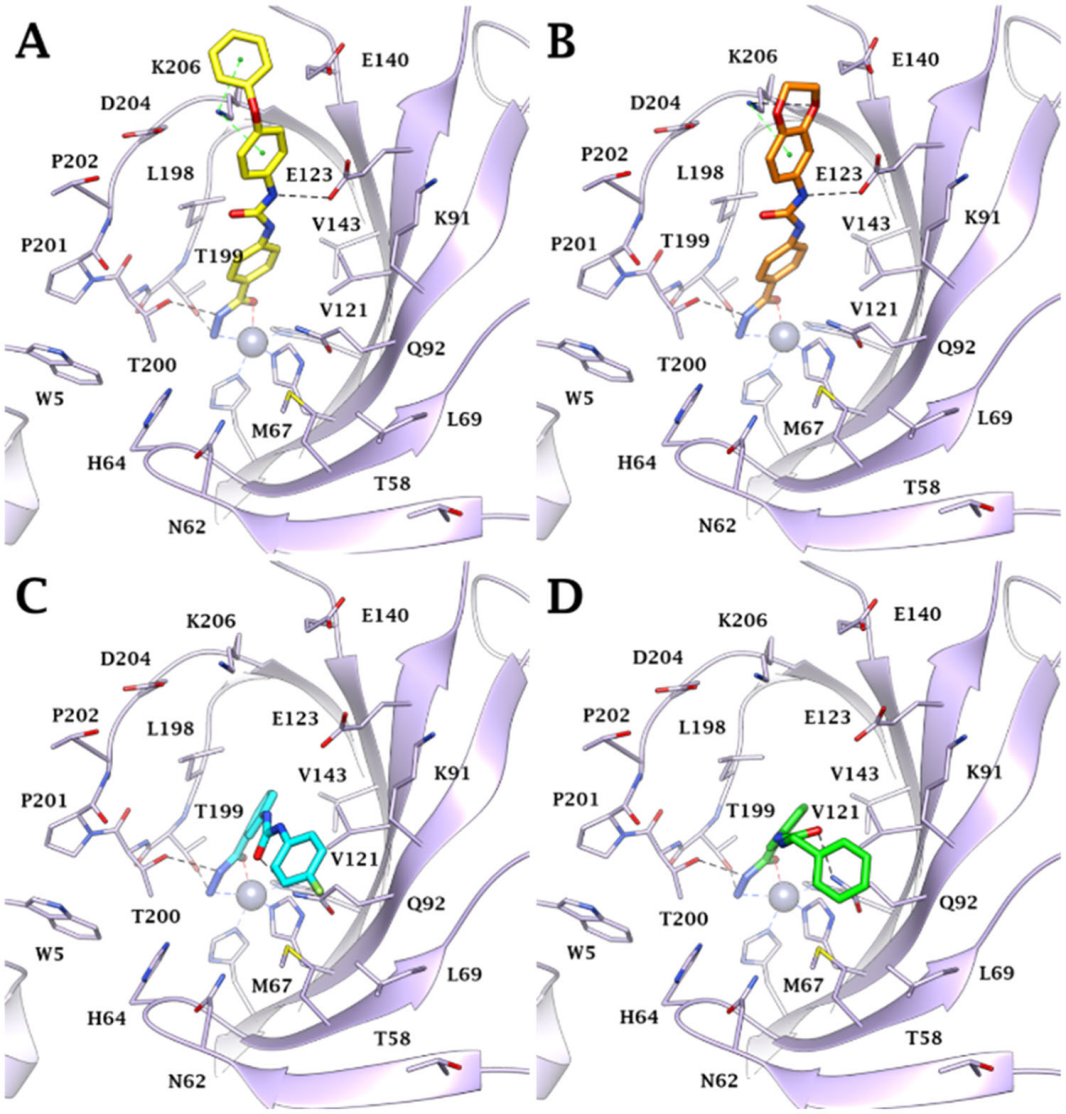
Predicted binding mode of compounds (A) **76** (yellow), (B) **78** (orange), (C) **79** (cyan), and (D) **83** (green) within the hCA IV active site. H‐bonds and π–cation interactions are depicted as black and green dashed lines, respectively.

**Figure 5:**
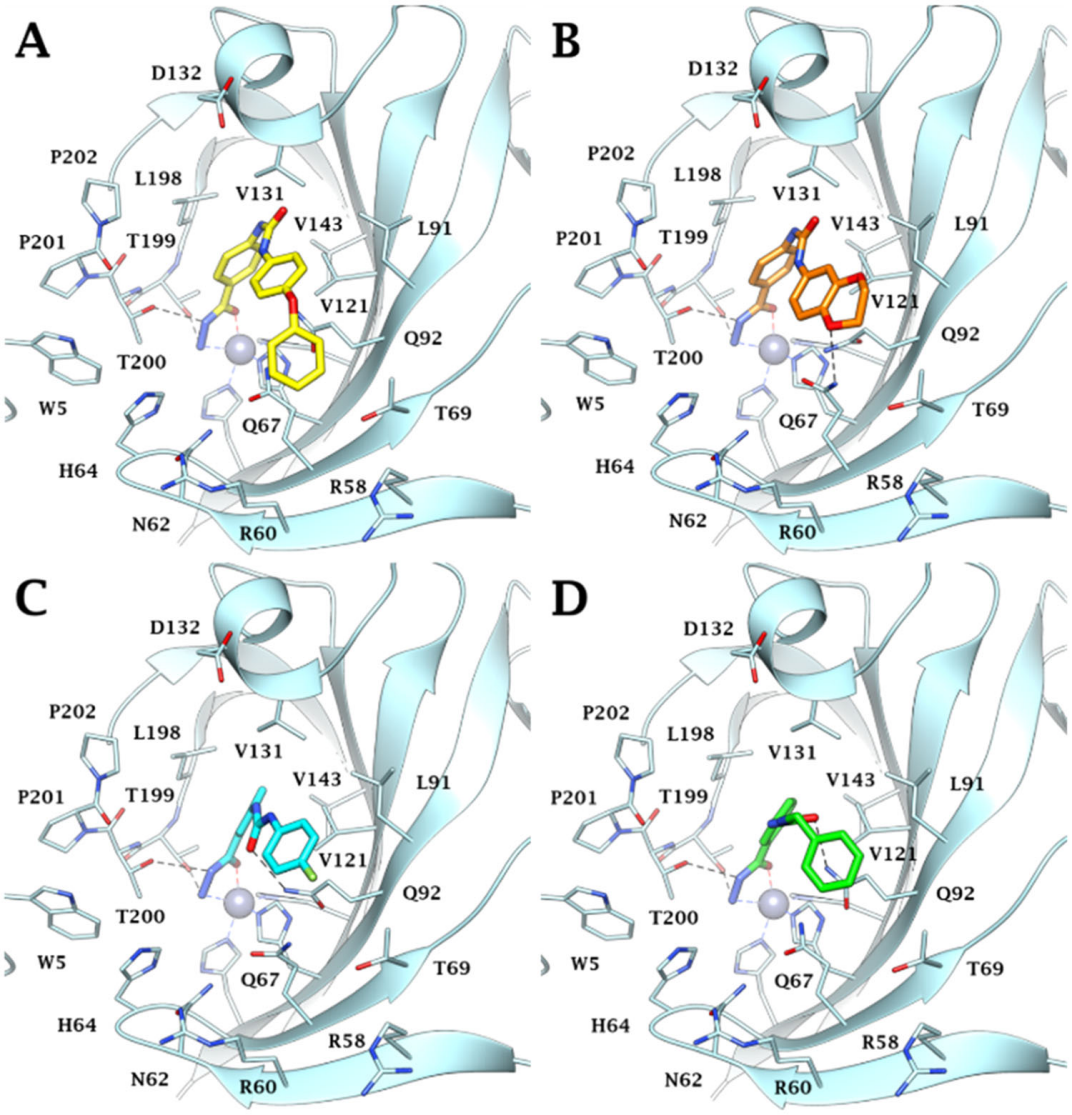
Predicted binding mode of compounds (A) **76** (yellow), (B) **78** (orange), (C) **79** (cyan), and (D) **83** (green) within the hCA IX active site. H‐bonds are depicted as black dashed lines.

**Figure 6:**
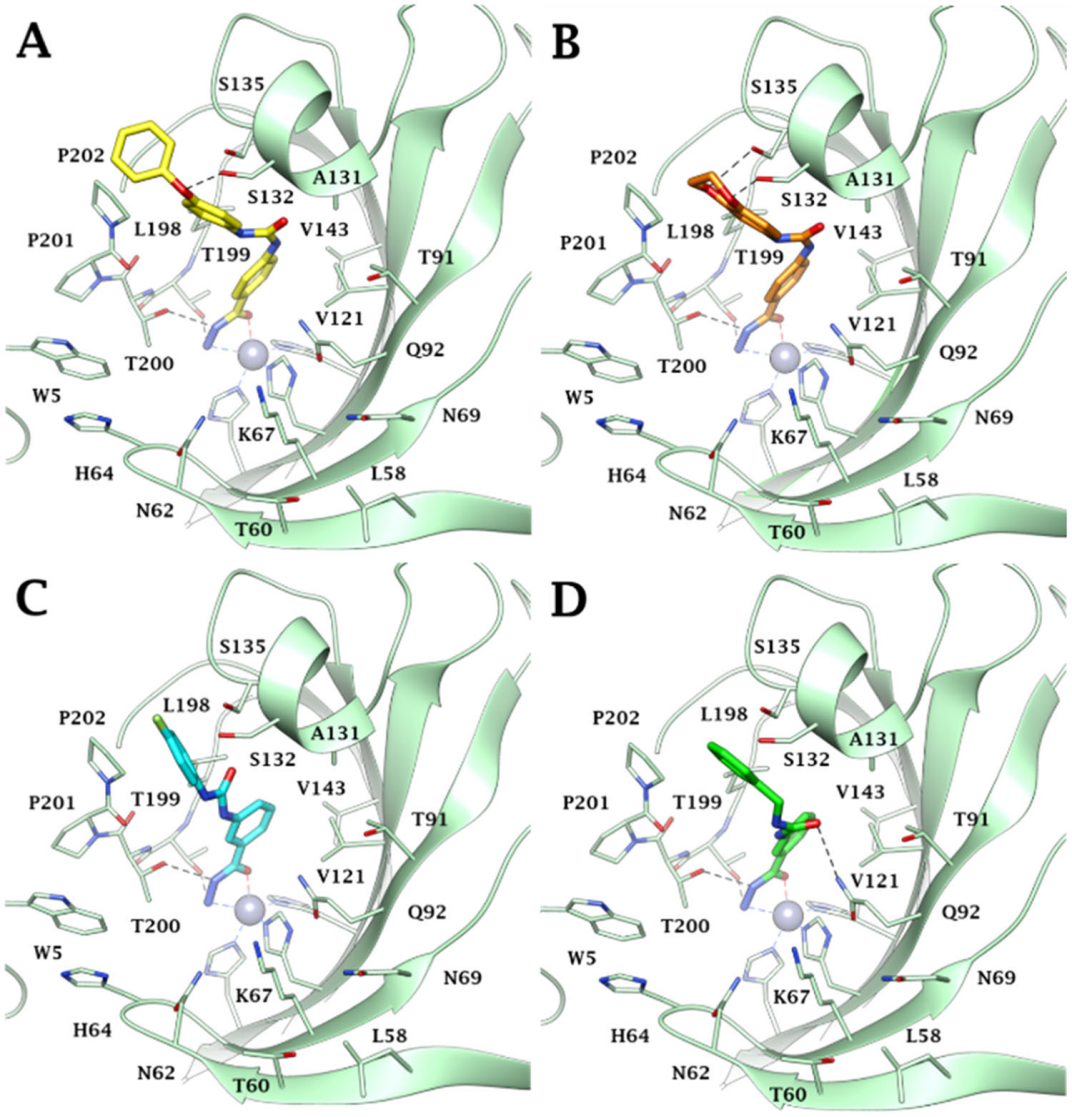
Predicted binding mode of compounds (A) **76** (yellow), (B) **78** (orange), (C) **79** (cyan), and (D) **83** (green) within the hCA XII active site. H‐bonds are depicted as black dashed lines.

Docking studies were carried out to investigate the binding mode of the most selective and potent compounds (**76**, **78**, **79**, **83**) against the most targeted isoforms hCA IV, IX, and XII. Low‐scoring solutions or lack of predictions, consistent with the literature data [29], are observed with hCAs I and II, whose narrow active site probably hinders the hydrazide binding mode. Indeed, the steric hindrance exerted by H200, H67, and F91 in hCA I and F131 in hCA II on the *para* and *meta*‐substituents on the benzene hydrazide scaffold prevents the correct *meta*l coordination, explaining the higher KI values found in vitro against these isoforms. Conversely, as reported in Reference [29], all docking solutions within the hCA IV, IX, and XII active sites found the ligands hydrazide group (CONHNH_2_) deeply bound to the zinc ion in a pentacoordinate geometry through the nitrogen and oxygen electron pairs of the terminal NH_2_ and C═O moiety, respectively (Figures 4, 5, 6). The ligand–target complexes stabilization is also supported by the formation of two H‐bonds between the hydrazide NH_2_ and C═O with the sidechain OH of T199 and T200, respectively, and van der Waals (vdW) contacts of the benzene ring with the lipophilic residues V121, V143, L198, and W205.

Within the hCA IV active site, the increased *K*_I_ values showed by the *para*‐substituted compounds **76** and **78** and their better selectivity with respect to hCA I and II could be attributable to their ability to engage in interactions with the peculiar residues E123 and K206 (Figure 4). Specifically, the urea NH forms a hydrogen bond with the sidechain COO^‐^ of E123 in both ligands. Meanwhile, the 4‐phenoxyphenyl tail of derivative **76** and the 2,3‐dihydrobenzo[b][1,4]dioxin‐6‐yl moiety of compound **78** engages in two π–cation interactions (for **76**, Figure 4A) and one hydrogen bond along with one π–cation interaction (for **78**, Figure 4B) with the sidechain NH_3_^+^ of K206.

On the other hand, the urea spacer C═O of the *meta*‐substituted ligands **79** and **83** is in H‐bond distance with the sidechain NH_2_ of the conserved Q92, orienting the aromatic tail toward an optimal lipophilic area lined by M67, L69, and the carbon chains of Q92 and K91 (Figure 4B,C).

Within the more spacious hCA IX active site, all ligands place their aromatic tails in a lipophilic pocket defined by residues T69, L91, Q92, V121, V131, and V143, engaging a wide network of vdW interactions (Figure 5). In particular, the longer ligand **76** extends its hydrophobic interactions to the carbon chains of the peculiar residues R58, R60, and Q67 through its terminal aromatic ring (Figure 5A), while derivative **78** is in H‐bond distance with the side chain NH_2_ of Q67 (Figure 5B). Instead, the urea linker C═O of *meta*‐substituted ligands **79** and **83** establishes an H‐bond with the sidechain NH_2_ of Q92 (Figure 5C,D).

In the hCA XII active site, while all ligands orient the aromatic tails toward a cleft lined by A131, S132, S135, P201, and P202 (Figure 6A–C), only the urea spacer C═O of ligand **83** is in H‐bond distance with the sidechain NH_2_ of Q92 (Figure 6D). Moreover, the high CA XII‐selectivity of *para*‐substituted derivatives **76** and **78** is explained by their ability to engage H‐bonds with the peculiar residues S132 and S135. Specifically, the oxygen atoms of the 4‐phenoxyphenyl pendant of compound **76** and the 2,3‐dihydrobenzo[b][1,4]dioxin‐6‐yl tail of derivative **78** engage an H‐bond with the side chain OH of S132 (for **76**, Figure 6A) and two H‐bonds with the side chain OH of S132 and S135 (for **78**, Figure 6B), respectively.

## 3 Conclusion

We successfully synthesized and evaluated a wide series of hydrazide‐based derivatives as inhibitors of human carbonic anhydrase (hCA) isoforms I, II, IV, IX, and XII, highlighting their potential as an alternative to sulfonamide CAIs. The inhibitory activity of these compounds, assessed using a CO_2_ hydrase stopped‐flow assay, demonstrated that ureido derivatives (**71**–**86**) exhibit the highest potency, with some of them reaching submicromolar inhibition constants (*K*_I_s: 0.8–0.96 µM) against hCA IV. Molecular modeling provided further insights into the binding mode of the most promising inhibitors, reinforcing the experimental findings. Such results suggest that hydrazide‐based compounds could represent promising leads for the development of targeted hCA inhibitors, particularly for hCA IV and the cancer‐associated IX and XII, as well as for other relevant hCA isoforms, offering an alternative to traditional CA inhibitors. Future research could focus on optimizing the potency and selectivity of these compounds to further explore their therapeutic potential.

## 4 Experimental

### 4.1 Chemistry

#### 4.1.1 General

Anhydrous solvents and all reagents were purchased from Merck, Fluorochem, and TCI. All reactions involving air‐ or moisture‐sensitive compounds were performed under a nitrogen atmosphere using dried glassware and syringe techniques to transfer solutions. Nuclear magnetic resonance (^1^H‐NMR, ^13^C‐NMR, ^19^F‐NMR) spectra (see the Supporting Information) were recorded using a Bruker Advance III 400 MHz spectrometer in DMSO‐*d*_6_. Chemical shifts are reported in parts per million (ppm), and the coupling constants (J) are expressed in Hertz (Hz). Splitting patterns are designated as follows: s, singlet; bs, broad singlet; d, doublet; t, triplet; q, quadruplet; m, multiplet; dd, doublet of doublets. The assignment of exchangeable protons was confirmed by the addition of D_2_O. Analytical thin‐layer chromatography (TLC) was carried out on Sigma‐Aldrich silica gel F‐254 plates. All spectra were in accord with the assigned structures. HRMS analyzes were performed on a Bruker Daltonics MicrOTOF‐Q II mass spectrometer. All final compounds were > 96% pure. The purity of target compounds was assessed by HPLC using an Agilent 1200 Series gradient HPLC system with a Luna PFP column (3 lm, 2 mm × 30 mm) at a flow rate of 0.25 mL/min and a linear gradient of the mobile phase—i.e., 10 mM formic acid and 5 mM ammonium formate in mQ water solution (solvent A) and 10 mM formic acid and 5 mM ammonium formate in methanol (solvent B).

The InChI codes of the investigated compounds, together with some biological activity data, are provided as Supporting Information.

#### 4.1.2 General Synthetic Procedure of Amido‐Based Intermediates **4** and **5**

The proper acyl chloride **2** or **3** (1 equiv.) was added dropwise at 0°C to a stirred suspension of methyl 4‐aminobenzoate **1** (0.2 g, 1 equiv.) and K_2_CO_3_ (1.5 equiv.) in acetone (10 mL), then the reaction mixture was stirred on at rt. After the completion of the reaction, the solvent was removed under vacuum, and H_2_O (30 mL) was added, forming a suspension that was filtered, yielding the intermediates **4** and **5**.

Methyl 4‐benzamidobenzoate (**4**): Compound **4** was obtained according to the general procedure earlier reported using benzoyl chloride (**2**) as the starting material. Yield 20%; silica gel TLC R_*f*_ 0.72 (MeOH/DCM 5% *v/v*); *δ*_H_ (400 MHz, DMSO‐*d*_6_): 10.55 (s, 1H, exchange with D_2_O, N*H)*, 7.97 (m, 6H, 6 x Ar‐*H*), 7.62 (dd, *J* = 7.22 Hz, 1H, Ar‐*H*), 7.55 (dd, *J* = 7.70 Hz, 2H, 2 x Ar‐*H*), 3.84 (s, 3H, C*H*_3_).

Methyl 4‐benzamidobenzoate (**5**): Compound **5** was obtained according to the general procedure earlier reported with 4‐nitrobenzoyl chloride (**3**) as starting material. Yield 30%; silica gel TLC R_*f*_ 0.62 (MeOH/DCM 5% *v/v*); *δ*_H_ (400 MHz, DMSO‐*d*_6_): 10.83 (s, 1H, exchange with D_2_O, N*H*), 8.37 (d, *J* = 8.6 Hz, 2H, 2 x Ar‐*H*), 8.19 (d, *J* = 8.6 Hz, 2H, 2 x Ar‐*H*), 7.98 (d, *J* = 8.85 Hz, 2H, 2 x Ar‐*H*), 7.94 (d, *J* = 8.85 Hz, 2H, 2 x Ar‐*H*), 3.84 (s, 3H, C*H*_3_).

#### 4.1.3 General Synthetic Procedure of Urea‐Based Intermediates **15‐30**

The proper isocyanates **7**–**14** (1 equiv.) were added dropwise to a stirred solution of **1** or **6** (0.2 g, 1 equiv.) in anhydrous ACN (5 mL) under a nitrogen atmosphere; then the reaction mixture was stirred on at rt. After the completion of the reaction, the resulting precipitates were filtered to yield intermediates **15–30.**

Methyl 4‐[3‐(4‐fluorophenyl)ureido]benzoate (**15**): Compound **15** was obtained according to the general procedure earlier reported with 1‐fluoro‐4‐isocyanatobenzene (**7**) and methyl 4‐aminobenzoate (**1**) as starting materials. Yield 77%; silica gel TLC R_*f*_ 0.75 (MeOH/DCM 5% *v/v*); *δ*_H_ (400 MHz, DMSO‐*d*_6_): 9.07 (s, 1H, exchange with D_2_O, N*H*CONH), 8.81 (s, 1H, exchange with D_2_O, NHCON*H*), 7.88 (d, *J* = 8.7 Hz, 2H, 2 x Ar‐*H*), 7.58 (d, *J* = 8.7 Hz, 2H, 2 x Ar‐*H*), 7.47 (m, 2H, 2 x Ar‐*H*), 7.13 (m, 2H, 2 x Ar‐*H*), 3.82 (s, 3H, C*H*_3_).

Methyl 4‐[3‐(4‐chlorophenyl)ureido]benzoate (**16**): Compound **16** was obtained according to the general procedure earlier reported with 1‐chloro‐4‐isocyanatobenzene (**8**) and methyl 4‐aminobenzoate (**1**) as starting materials. Yield 65%; silica gel TLC R_*f*_ 0.65 (MeOH/DCM 5% *v/v*); *δ*_H_ (400 MHz, DMSO‐*d*_6_): 9.09 (s, 1H, exchange with D_2_O, N*H*CONH), 8.90 (s, 1H, exchange with D_2_O, NHCON*H*), 7.89 (d, *J* = 8.5 Hz, 2H, 2 x Ar‐*H*), 7.58 (d, *J* = 8.5 Hz, 2H, 2 x Ar‐*H*), 7.49 (d, *J* = 8.7 Hz, 2H, 2 x Ar‐*H*), 7.34 (d, *J* = 8.7 Hz, 2H, 2 x Ar‐*H*), 3.82 (s, 3H, C*H*_3_).

Methyl 4‐{3‐[4‐(trifluoromethoxy)phenyl]ureido}benzoate (**17**): Compound **17** was obtained according to the general procedure earlier reported with 1‐isocyanato‐4‐(trifluoromethoxy)benzene (**9**) and methyl 4‐aminobenzoate (**1**) as starting materials. Yield 30%; silica gel TLC R_*f*_ 0.64 (MeOH/DCM 5% *v/v*); *δ*_H_ (400 MHz, DMSO‐*d*_6_): 9.14 (s, 1H, exchange with D_2_O, N*H*CONH), 9.00 (s, 1H, exchange with D_2_O, NHCON*H*), 7.89 (d, *J* = 8.6 Hz, 2H, 2 x Ar‐*H*), 7.58 (m, 4H, 4 x Ar‐*H*), 7.29 (d, *J* = 8.6 Hz, 2H, 2 x Ar‐*H*), 3.82 (s, 3H, C*H*_3_).

Methyl 4‐[3‐(furan‐2‐ylmethyl)ureido]benzoate (**18**): Compound **18** was obtained according to the general procedure earlier reported with 2‐(isocyanatomethyl)furan (**10**) and methyl 4‐aminobenzoate (**1**) as starting materials. Yield 45%; silica gel TLC R_*f*_ 0.67 (MeOH/DCM 5% *v/v*); *δ*_H_ (400 MHz, DMSO‐*d*_6_): 8.97 (s, 1H, exchange with D_2_O, N*H*CONH), 7.84 (d, *J* = 8.5 Hz, 2H, 2 x Ar‐*H*), 7.58 (s, 1H, Ar‐*H*), 7.52 (d, *J* = 8.5 Hz, 2H, 2 x Ar‐*H*) 6.72 (t, *J* = 5.5 Hz, 1H, exchange with D_2_O, NHCON*H*), 6.39 (s, 1H, Ar‐*H*), 6.27 (m, 1H, Ar‐*H*), 4.30 (d, *J* = 5.5 Hz, 2H, C*H*_2_), 3.79 (s, 3H, C*H*_3_).

Methyl 4‐(3‐benzylureido)benzoate (**19**): Compound **19** was obtained according to the general procedure earlier reported with (isocyanatomethyl)benzene (**11**) and methyl 4‐aminobenzoate (**1**) as starting materials. Yield 55%; silica gel TLC R_*f*_ 0.68 (MeOH/DCM 5% *v/v*); *δ*_H_ (400 MHz, DMSO‐*d*_6_): 9.01 (s, 1H, exchange with D_2_O, N*H*CONH), 7.84 (d, *J* = 8.8 Hz, 2H, 2 x Ar‐*H*), 7.54 (d, *J* = 8.8 Hz, 2H, 2 x Ar‐*H*), 7.33 (m, 4H, 4 x Ar‐*H*), 7.24 (m, 1H, Ar‐*H*), 6.79 (t, *J* = 5.9 Hz, 1H, exchange with D_2_O, NHCON*H*), 4.31 (d, *J* = 5.9 Hz, 2H, C*H*_2_), 3.79 (s, 3H, C*H*_3_).

Methyl 4‐[3‐(4‐phenoxyphenyl)ureido]benzoate (**20**): Compound **20** was obtained according to the general procedure earlier reported with 1‐isocyanato‐4‐phenoxybenzene (**12**) and methyl 4‐aminobenzoate (**1**) as starting materials. Yield 55%; silica gel TLC R_*f*_ 0.72 (MeOH/DCM 5% *v/v*); *δ*_H_ (400 MHz, DMSO‐*d*_*6*_): 9.06 (s, 1H, exchange with D_2_O, N*H*CONH), 8.80 (s, 1H, exchange with D_2_O, NHCON*H*), 7.89 (d, *J* = 8.7 Hz, 2H, 2 x Ar‐*H*), 7.59 (d, *J* = 8.7 Hz, 2H, 2 x Ar‐*H*), 7.48 (d, *J* = 8.7 Hz, 2H, 2 x Ar‐*H*), 7.36 (dd, *J* = 7.6 Hz, 2H, 2 x Ar‐*H*), 7.09 (dd, *J* = 7.6 Hz), 6.98 (m, 4H, 4 x Ar‐*H*), 3.81 (s, 3H, C*H*_3_).

Methyl 4‐[3‐(3‐methoxyphenyl)ureido]benzoate (**21**): Compound **21** was obtained according to the general procedure earlier reported with 1‐isocyanato‐3‐methoxybenzene (**13**) and methyl 4‐aminobenzoate (**1**) as starting materials. Yield 50%; silica gel TLC R_*f*_ 0.60 (MeOH/DCM 5% *v/v*); *δ*_H_ (400 MHz, DMSO‐*d*_*6*_): 9.05 (s, 1H, exchange with D_2_O, N*H*CONH), 8.79 (s, 1H, exchange with D_2_O, NHCON*H*), 7.89 (d, *J* = 8.8 Hz, 2H, 2 x Ar‐*H*), 7.58 (d, *J* = 8.8 Hz, 2H, 2 x Ar‐*H*), 7.19 (m, 2H, 2 x Ar‐*H*), 6.94 (dd, *J* = 8.2, 1.9 Hz, 1H, Ar‐*H*), 6.58 (dd, *J* = 8.2, 1.9 Hz, 1H, Ar‐*H*), 3.81 (s, 3H, C*H*_3_), 3.73 (s, 3H, C*H*_3_).

Methyl 4‐[3‐(2,3‐dihydrobenzo[*b*][1,4]dioxin‐6‐yl)ureido]benzoate (**22**): Compound **22** was obtained according to the general procedure earlier reported with 6‐isocyanato‐2,3‐dihydrobenzo[b][1,4]dioxine **14** and methyl 4‐aminobenzoate **1** as starting materials. Yield 80%; silica gel TLC R_*f*_ 0.56 (MeOH/DCM 5% *v/v*); *δ*_H_ (400 MHz, DMSO‐*d*_*6*_): 8.99 (s, 1H, exchange with D_2_O, N*H*CONH), 8.59 (s, 1H, exchange with D_2_O, NHCON*H*), 7.87 (d, *J* = 8.8 Hz, 2H, 2 x Ar‐*H*), 7.56 (d, *J* = 8.8 Hz, 2H, 2 x Ar‐*H*), 7.10 (d, *J* = 2.3 Hz, 1H, Ar‐*H*), 6.79 (m, 2H, 2 x Ar‐*H*), 4.20 (m, 4H, 2 x C*H*_2_), 3.81 (s, 3H, C*H*_3_).

Methyl 3‐[3‐(4‐fluorophenyl)ureido]benzoate (**23**): Compound **23** was obtained according to the general procedure earlier reported with 1‐fluoro‐4‐isocyanatobenzene (**7**) and methyl 3‐aminobenzoate (**6**) as starting materials. Yield 60%; silica gel TLC R_*f*_ 0.62 (MeOH/DCM 5% *v/v*); *δ*_H_ (400 MHz, DMSO‐*d*_6_): 9.75 (s, 1H, exchange with D_2_O, N*H*CONH), 9.59 (s, 1H, exchange with D_2_O, NHCON*H*), 8.22 (s, 1H, Ar‐H), 7.67 (d, *J* = 7.5 Hz, 1H, Ar‐*H*), 7.53 (m, 3H, 3 x Ar‐*H*), 7.40 (dd, *J* = 7.4 Hz, 1H, Ar‐*H*), 7.11 (m, 1H, Ar‐*H*), 3.85 (s, 3H, C*H*_3_).

Methyl 3‐[3‐(4‐chlorophenyl)ureido]benzoate (**24**): Compound **24** was obtained according to the general procedure earlier reported with 1‐chloro‐4‐isocyanatobenzene (**8**) and methyl 3‐aminobenzoate (**6**) as starting materials. Yield 45%; silica gel TLC R_*f*_ 0.63 (MeOH/DCM 5% *v/v*); *δ*_H_ (400 MHz, DMSO‐*d*_6_): 9.56 (bs, 2H, exchange with D2O, NHCONH), 8.22 (s, 1H, Ar‐H), 7.66 (d, *J* = 7.9 Hz, 1H, Ar‐H), 7.53 (m, 3H, Ar‐H), 7.40 (dd, *J* = 7.9 Hz, 1H, Ar‐H), 7.30 (d, *J* = 8.9 Hz, 2H, 2 x Ar‐H), 3.84 (s, 3H, CH3).

Methyl 3‐{3‐[4‐(trifluoromethoxy)phenyl]ureido}benzoate (**25**): Compound **25** was obtained according to the general procedure earlier reported with 1‐isocyanato‐4‐(trifluoromethoxy)benzene (**9**) and methyl 3‐aminobenzoate (**6**) as starting materials. Yield 30%; silica gel TLC R_*f*_ 0.62 (MeOH/DCM 5% *v/v*); δ_H_ (400 MHz, DMSO‐*d*_6_): 9.75 (bs, 2H, exchange with D_2_O, 2 x N*H*CON*H*), 8.22 (s, 1H, Ar‐*H*), 7.67 (d, *J* = 8.1 Hz, 1H, Ar‐*H*), 7.60 (dd, *J* = 8.8 Hz, 2H, 2 x Ar‐*H*), 7.54 (d, *J* = 8.1 Hz, 1H, Ar‐*H*), 7.39 (dd, *J* = 8.1 Hz, 1H, Ar‐*H*), 7.25 (d, *J* = 8.8 Hz), 3.84 (s, 3H, C*H*_3_).

Methyl 3‐[3‐(furan‐2‐ylmethyl)ureido]benzoate (**26**): Compound **26** was obtained according to the general procedure earlier reported with 2‐(isocyanatomethyl)furan (**10**) and methyl 3‐aminobenzoate (**6**) as starting materials. Yield 60%; silica gel TLC R_*f*_ 0.70 (MeOH/DCM 5% *v/v*); δ_H_ (400 MHz, DMSO‐*d*_6_): 8.79 (s, 1H, exchange with D_2_O, N*H*CONH), 8.12 (dd, *J* = 1.8 Hz, 1H, Ar‐*H*), 7.58 (m, 2H, 2 x Ar‐*H*), 7.50 (m, 1H, Ar‐*H*), 7.37 (dd, *J* = 7.9 Hz, 1H, Ar‐*H*), 6.59 (t, *J* = 5.8 Hz, 1H, exchange with D_2_O, NHCON*H*), 6.9 (m, 1H, Ar‐*H*), 6.26 (d, *J* = 2.8 Hz, 1H, Ar‐*H*), 4.29 (d, *J* = 5.8 Hz, 2H, C*H*_2_), 3.83 (s, 3H, C*H*_3_).

Methyl 3‐(3‐benzylureido)benzoate (**27**): Compound **27** was obtained according to the general procedure earlier reported with (isocyanatomethyl)benzene (**11**) and methyl 3‐aminobenzoate (**6**) as starting materials. Yield 60%; silica gel TLC R_*f*_ 0.70 (MeOH/DCM 5% *v/v*); *δ*_H_ (400 MHz, DMSO‐*d*_6_): 8.83 (s, 1H, exchange with D_2_O, N*H*CONH), 8.14 (s, 1H, Ar‐*H*), 7.60 (d, *J* = 8.5 Hz, 1H, Ar‐*H*), 7.50 (d, *J* = 8.5 Hz, 2H, 2 x Ar‐*H*), 7.33 (m, 4H, 4 x Ar‐*H*), 7.24 (m, 1H, Ar‐*H*), 6.79 (t, *J* = 5.9 Hz, 1H, exchange with D_2_O, NHCON*H*), 4.31 (d, *J* = 5.9 Hz, 2H, C*H*_2_), 3.79 (s, 3H, C*H*_3_).

Methyl 3‐[3‐(4‐phenoxyphenyl)ureido]benzoate (**28**): Compound **28** was obtained according to the general procedure earlier reported with 1‐isocyanato‐4‐phenoxybenzene (**12**) and methyl 3‐aminobenzoate (**6**) as starting materials. Yield 80%; silica gel TLC R_*f*_ 0.73 (MeOH/DCM 5% *v/v*); δ_H_ (400 MHz, DMSO‐*d*_6_): 8.91 (s, 1H, exchange with D_2_O, N*H*CONH), 8.71 (s, 1H, exchange with D_2_O, NHCON*H*), 8.20 (s, 1H, Ar‐*H*), 7.63 (d, *J* = 7.8 Hz, 1H, Ar‐*H*), 7.56 (d, *J* = 7.8 Hz, 1H, Ar‐*H*), 7.49 (m, 2H, 2 x Ar‐*H*), 7.42 (dd, *J* = 7.8 Hz, 1H, Ar‐*H*), 7.36 (dd, *J* = 7.9 Hz, 1H, Ar‐*H*), 7.09 (dd, *J* = 7.8 Hz, 1H, Ar‐*H*), 6.97 (m, 4H, 4 x Ar‐*H*), 3.85 (s, 3H, C*H*_3_).

Methyl 3‐[3‐(3‐methoxyphenyl)ureido]benzoate (**29**): Compound **29** was obtained according to the general procedure earlier reported with 1‐isocyanato‐3‐methoxybenzene (**13**) and methyl 3‐aminobenzoate (**6**) as starting materials. Yield 80%; silica gel TLC R_*f*_ 0.68 (MeOH/DCM 5% *v/v*); δ_H_ (400 MHz, DMSO‐*d*_6_): 8.91 (s, 1H, exchange with D_2_O, N*H*CONH), 8.70 (s, 1H, exchange with D_2_O, NHCON*H*), 8.19 (s, 1H, Ar‐*H*), 7.62 (d, *J* = 8.0 Hz, 1H, Ar‐*H*), 7.57 (d, *J* = 8.0 Hz, 1H, Ar‐*H*), 7.42 (dd, *J* = 8.0 Hz, 1H, Ar‐*H*), 7.19 (d, *J* = 8.0 Hz, 1H, Ar‐*H*), 7.16 (s, 1H, Ar‐*H*), 6.95 (d, *J* = 8.0 Hz, 1H, Ar‐*H*), 6.57 (d, *J* = 8.0 Hz, 1H, Ar‐*H*), 3.86 (s, 3H, C*H*_3_), 3.73 (s, 3H, C*H*_3_).

Methyl 3‐[3‐(2,3‐dihydrobenzo[*b*][1,4]dioxin‐6‐yl)ureido]benzoate (**30**): Compound **30** was obtained according to the general procedure earlier reported with 6‐isocyanato‐2,3‐dihydrobenzo[b][1,4]dioxine (**14**) and methyl 3‐aminobenzoate (**6**) as starting materials. Yield 79%; silica gel TLC R_*f*_ 0.58 (MeOH/DCM 5% *v/v*); *δ*_H_ (400 MHz, DMSO‐*d*_6_): 8.83 (s, 1H, exchange with D_2_O, N*H*CONH), 8.49 (s, 1H, exchange with D_2_O, NHCON*H*), 8.16 (s, 1H, Ar‐*H*), 7.60 (d, *J* = 7.9 Hz, 1H, Ar‐*H*), 7.55 (d, *J* = 7.9 Hz, 1H, Ar‐*H*), 7.40 (dd, *J* = 7.9 Hz), 7.09 (d, *J* = 2.3 Hz, 1H, Ar‐*H*), 6.81 (d, *J* = 8.6 Hz, 1H, Ar‐*H*), 6.76 (d, *J* = 8.6 Hz, 1H, Ar‐*H*), 4.20 (m, 4H, 2 x C*H*_2_), 3.85 (s, 3H, C*H*_3_).

#### 4.1.4 General Synthetic Procedure of Hydrazide‐Based Derivatives **49‐86**

Hydrazine hydrate (10 equiv.) was added to a stirred solution of the proper methyl esters **1**, **4**–**6**, **15**–**48** (0.3 g, 1 equiv.) in anhydrous methanol (5 mL), then anhydrous pyridine (0.1 equiv.) was added. The resulted reaction mixture was refluxed on. After completion of the reaction, the resulting precipitates were filtered and purified by crystallization from EtOAc, affording the hydrazide‐based compounds **49**–**86**.

4‐Nitrobenzohydrazide (**49**): Compound **49** was obtained according to the general procedure earlier reported, with methyl 4‐nitrobenzoate (**31**) as the starting material. Yield 30%; silica gel TLC R_*f*_ 0.25 (MeOH/DCM 5% *v/v*); *δ*_H_ (400 MHz, DMSO‐*d*_6_): 10.11 (s, 1H, exchange with D_2_O, N*H*), 8.29 (d, *J* = 8.5 Hz, 2H, 2 x Ar‐*H*), 8.04 (d, *J* = 8.5 Hz, 2H, 2 x Ar‐*H*), 4.64 (s, 2H, exchange with D_2_O, N*H*_2_); *δ*_C_ (100 MHz, DMSO‐*d*_6_): 164.96, 149.99, 140.08, 129.51, 124.62; ESI‐HRMS (*m/z*) [M‐H]^−^: calculated for C_7_H_7_N_3_O_3_ 181.0487; found 181.0493.

3‐Nitrobenzohydrazide (**50**): Compound **50** was obtained according to the general procedure earlier reported, with methyl 3‐nitrobenzoate (**32**) as the starting material. Yield 24%; silica gel TLC R_*f*_ 0.25 (MeOH/DCM 5% *v/v*); *δ*_H_ (400 MHz, DMSO‐*d*_6_): 10.14 (s, 1H, exchange with D_2_O, N*H*), 8.62 (s, 1H, Ar‐*H*), 8.36 (d, *J* = 7.8 Hz, 2H, 2 x Ar‐*H*), 8.25 (d, *J* = 7.8 Hz, 1H, Ar‐*H*), 7.76 (dd, *J* = 7.8 Hz, 1H, Ar‐*H*), 4.61 (s, 2H, exchange with D_2_O, N*H*_2_); δ_C_ (100 MHz, DMSO‐*d*_6_): 164.61, 148.85, 135.79, 134.31, 131.23, 126.78, 122.86; ESI‐HRMS (*m/z*) [M‐H]^−^: calculated for C_7_H_7_N_3_O_3_ 181.0487; found 181.0482.

4‐Aminobenzohydrazide (**51**): Compound **52** was obtained according to the general procedure earlier reported with methyl 4‐aminobenzoate (**1**) as the starting material. Yield 65%; silica gel TLC R_*f*_ 0.24 (MeOH/DCM 5% *v/v*); *δ*_H_ (400 MHz, DMSO‐*d*_6_): 9.25 (s, 1H, exchange with D_2_O, N*H*), 7.55 (d, *J* = 8.5 Hz, 2H, 2 x Ar‐*H*), 6.52 (d, *J* = 8.5 Hz, 2H, 2 x Ar‐*H*), 5.55 (s, 1H, exchange with D_2_O, N*H*_2_), 4.29 (s, 2H, exchange with D_2_O, N*H*_2_); δ_C_ (100 MHz, DMSO‐*d*_6_): 167.50, 152.59, 129.48, 120.97, 113.64; ESI‐HRMS (*m/z*) [M‐H]^−^: calculated for C_7_H_9_N_3_O 151.0746; found 151.0751.

3‐Aminobenzohydrazide (**52**): Compound (**52**) was obtained according to the general procedure earlier reported with methyl 3‐aminobenzoate (**6**) as the starting material. Yield 55%; silica gel TLC R_*f*_ 0.26 (MeOH/DCM 5% *v/v*); δ_H_ (400 MHz, DMSO‐*d*_6_): 9.51 (s, 1H, exchange with D_2_O, N*H*), 7.03 (m, 2H, Ar‐*H*), 6.91 (d, *J* = 7.5 Hz, 1H, Ar‐*H*), 6.66 (d, *J* = 7.5 Hz, 1H, Ar‐*H*), 5.20 (s, 2H, exchange with D_2_O, N*H*_2_), 4.42 (s, 2H, exchange with D_2_O, N*H*_2_); δ_C_ (100 MHz, DMSO‐*d*_6_): 167.82, 149.70, 135.25, 129.72, 117.39, 115.05, 113.80; ESI‐HRMS (*m/z*) [M‐H]^−^: calculated for C_7_H_9_N_3_O 151.0746; found 151.0755.

1*H*‐Indole‐3‐carbohydrazide (**53**): Compound **53** was obtained according to the general procedure earlier reported with methyl 1H‐indole‐3‐carboxylate (**33**) as the starting material. Yield 21%; silica gel TLC R_*f*_ 0.30 (MeOH/DCM 5% *v/v*); *δ*_H_ (400 MHz, DMSO‐*d*_6_): 11.52 (s, 1H, exchange with D_2_O, N*H*), 9.15 (s, 1H, exchange with D_2_O, N*H*), 8.14 (d, *J* = 7.9 Hz, 1H, Ar‐*H*), 7.97 (s, 1H, Ar‐H), 7.42 (d, *J* = 7.9 Hz, 1H, Ar‐*H*), 7.11 (m, 2H, 2 x Ar‐*H*), 4.29 (s, 2H, exchange with D_2_O, N*H*_2_); δ_C_ (100 MHz, DMSO‐*d*_6_): 166.21, 137.03, 128.11, 127.20, 122.88, 121.98, 121.34, 112.82, 110.01; ESI‐HRMS (*m/z*) [M‐H]^−^: calculated for C_9_H_9_N_3_O 175.0746; found 175.0740.

1*H*‐Indole‐2‐carbohydrazide (**54**): Compound **54** was obtained according to the general procedure earlier reported with methyl 1H‐indole‐2‐carboxylate (**34**) as the starting material. Yield 61%; silica gel TLC R_*f*_ 0.27 (MeOH/DCM 5% *v/v*); δ_H_ (400 MHz, DMSO‐*d*_6_): 11.60 (s, 1H, exchange with D_2_O, N*H*), 9.79 (s, 1H, exchange with D_2_O, N*H*), 7.58 (d, *J* = 7.7 Hz, 1H, Ar‐*H*), 7.44 (d, *J* = 7.7 Hz, 1H, Ar‐H) 7.17 (dd, *J* = 7.7 Hz, 1H, Ar‐H), 7.10 (s, 1H, Ar‐*H*), 7.02 (dd, *J* = 7.7 Hz, 1H, Ar‐H), 4.52 (s, 2H, exchange with D_2_O, N*H*_2_); δ_C_ (100 MHz, DMSO‐*d*_6_): 162.29, 137.37, 131.56, 128.17, 124.16, 122.46, 120.76, 113.31, 102.88; ESI‐HRMS (*m/z*) [M‐H]^−^: calculated for C_9_H_9_N_3_O 175.0746; found 175.0751.

1*H*‐Indazole‐3‐carbohydrazide (**55**): Compound **55** was obtained according to the general procedure earlier reported with methyl 1H‐indazole‐3‐carboxylate (**35**) as the starting material. Yield 61%; silica gel TLC R_*f*_ 0.29 (MeOH/DCM 5% *v/v*); *δ*_H_ (400 MHz, DMSO‐*d*_6_): 13.49 (s, 1H, exchange with D_2_O, N*H*), 9.55 (s, 1H, exchange with D_2_O, N*H*), 8.16 (d, *J* = 8.0 Hz, 1H, Ar‐*H*), 7.61 (d, *J* = 8.0 Hz, 1H, Ar‐*H*), 7.42 (dd, *J* = 8.0 Hz, 1H, Ar‐*H*), 7.25 (dd, *J* = 8.0 Hz, 1H, Ar‐*H*), 4.48 (s, 2H, exchange with D_2_O, N*H*_2_); *δ*_C_ (100 MHz, DMSO‐*d*_6_): 163.06, 141.87, 138.59, 127.53, 122.94, 122.62, 122.46, 111.68; ESI‐HRMS (*m/z*) [M‐H]^−^: calculated for C_8_H_8_N_4_O 176.0698; found 176.0691.

Benzofuran‐5‐carbohydrazide (**56**): Compound **56** was obtained according to the general procedure earlier reported with methyl benzofuran‐5‐carboxylate (**36**) as the starting material. Yield 4%; silica gel TLC R_*f*_ 0.29 (MeOH/DCM 5% *v/v*); δ_H_ (400 MHz, DMSO‐*d*_6_): 9.76 (s, 1H, exchange with D_2_O, N*H*), 8.15 (s, 1H, Ar‐*H*), 8.07 (d, *J* = 1.9 Hz, 1H, Ar‐*H*), 7.80 (dd, *J* = 8.6 1.9 Hz, 1H, Ar‐H), 7.64 (d, *J* = 8.6 Hz, 1H, Ar‐*H*), 7.04 (d, *J* = 1.9 Hz, 1H, Ar‐*H*), 4.37 (bs, 2H, exchange with D_2_O, N*H*_2_); *δ*_C_ (100 MHz, DMSO‐*d*_6_): 162.29, 137.37, 131.56, 128.17, 124.16, 122.46, 120.76, 113.31, 102.88; ESI‐HRMS (*m/z*) [M‐H]^−^: calculated for C_9_H_8_N_2_O_2_ 176.0586; found 176.0595.

Naphthalene‐2‐carbohydrazide (**57**): Compound **57** was obtained according to the general procedure earlier reported with methyl naphthalene‐2‐carboxylate (**37**) as the starting material. Yield 61%; silica gel TLC R_*f*_ 0.31 (MeOH/DCM 5% *v/v*); δ_H_ (400 MHz, DMSO‐*d*_6_): 9.91 (s, 1H, exchange with D_2_O, N*H*), 8.42 (s, 1H, Ar‐*H*), 7.98 (m, 3H, 3 x Ar‐*H*), 7.91 (dd, *J* = 8.6 1.6 Hz, 1H, Ar‐*H*), 7.59 (m, 2H, 2 x Ar‐*H*), 4.56 (s, 2H, exchange with D_2_O, N*H*_2_); δ_C_ (100 MHz, DMSO‐*d*_6_): 166.97, 135.13, 133.24, 131.73, 129.89, 128.95, 128.66, 128.58, 128.34, 127.75, 124.92; ESI‐HRMS (*m/z*) [M‐H]^−^: calculated for C_11_H_10_N_2_O 186.0793; found 186.0785.

Naphthalene‐1‐carbohydrazide (**58**): Compound **58** was obtained according to the general procedure earlier reported with methyl naphthalene‐1‐carboxylate (**38**) as the starting material. Yield 61%; silica gel TLC R_*f*_ 0.33 (MeOH/DCM 5% *v/v*); *δ*_H_ (400 MHz, DMSO‐*d*_6_): 9.69 (s, 1H, exchange with D_2_O, N*H*), 8.21 (m, 1H, Ar‐*H*), 7.99 (m, 2H, 2 x Ar‐*H*), 7.55 (m, 4H, 4 x Ar‐*H*), 4.54 (bs, 2H, exchange with D_2_O, N*H*_2_); *δ*_C_ (100 MHz, DMSO‐*d*_6_): 169.04, 134.42, 134.17, 131.05, 130.90, 129.24, 127.69, 127.29, 126.48, 126.40, 126.05; ESI‐HRMS (*m/z*) [M‐H]^−^: calculated for C_11_H_10_N_2_O 186.0793; found 186.0782.

3,5‐Diethoxybenzohydrazide (**59**): Compound **59** was obtained according to the general procedure earlier reported with methyl 3,5‐diethoxybenzoate (**39**) as the starting material. Yield 56%; silica gel TLC R_*f*_ 0.18 (MeOH/DCM 5% *v/v*); δ_H_ (400 MHz, DMSO‐*d*_6_): 9.71 (s, 1H, exchange with D_2_O, N*H*), 6.96 (d, *J* = 2.4 Hz, 2H, Ar‐*H*), 6.59 (dd, *J* = 2.4 Hz, 1H, Ar‐*H*), 4.05 (q, *J* = 6.9 Hz, 4H, 2 x C*H*_2_), 1.33 (t, *J* = 6.9 Hz, 6H, 2 x C*H*_3_), 4.40 (bs, 2H, exchange with D_2_O, N*H*_2_); δ_C_ (100 MHz, DMSO‐*d*_6_): 166.46, 160.57, 136.29, 106.20, 104.85, 64.33, 15.65; ESI‐HRMS (*m/z*) [M‐H]^−^: calculated for C_11_H_16_N_2_O_3_ 224.1161; found 224.1169.

4‐Oxo‐4*H*‐chromene‐2‐carbohydrazide (**60**): Compound **60** was obtained according to the general procedure earlier reported with methyl 4‐oxo‐4H‐chromene‐2‐carboxylate (**40**) as the starting material. Yield 59%; silica gel TLC R_*f*_ 0.26 (MeOH/DCM 5% *v/v*); *δ*_H_ (400 MHz, DMSO‐*d*_6_): 9.95 (s, 1H, exchange with D_2_O, N*H*), 7.64 (d, *J* = 7.5 Hz, 1H, Ar‐*H*), 7.19 (m, 2H, 2 x Ar‐*H*), 6.96 (d, *J* = 7.5 Hz, 1H, Ar‐H), 6.90 (dd, J = 7.5 Hz, 1H, Ar‐*H*), 4.46 (s, 2H, exchange with D_2_O, N*H*_2_); δ_C_ (100 MHz, DMSO‐*d*_6_): 179.74, 162.98, 117.63, 161.38, 155.63, 130.24, 128.12, 120.41, 117.49, 104.58; ESI‐HRMS (*m/z*) [M‐H]^−^: calculated for C_10_H_8_N_2_O_3_ 204.0535; found 204.0542.

5‐Bromopyrimidine‐2‐carbohydrazide (**61**): Compound **61** was obtained according to the general procedure earlier reported with methyl 5‐bromopyrimidine‐2‐carboxylate (**41**) as the starting material. Yield 22%; silica gel TLC R_*f*_ 0.20 (MeOH/DCM 5% *v/v*); *δ*_H_ (400 MHz, DMSO‐*d*_6_): 10.09 (s, 1H, exchange with D_2_O, N*H*), 9.11 (s, 2H, 2 x Ar‐*H*), 4.64 (s, 2H, exchange with D_2_O, N*H*_2_); *δ*_C_ (100 MHz, DMSO‐*d*_6_): 161.75, 159.27, 157.47, 122.80; ESI‐HRMS (*m/z*) [M‐H]^−^: calculated for C_5_H_5_BrN_4_O 215.9647; found 215.9655.

2‐Methylbenzohydrazide (**62**): Compound **62** was obtained according to the general procedure earlier reported with methyl 2‐methylbenzoate (**42**) as the starting material. Yield 7%; silica gel TLC R_*f*_ 0.27 (MeOH/DCM 5% *v/v*); *δ*_H_ (400 MHz, DMSO‐*d*_6_): 9.37 (s, 1H, exchange with D_2_O, N*H*), 7.26 (m, 4H, 4 x Ar‐*H*), 4.42 (s, 2H, exchange with D_2_O, N*H*_2_), 2.32 (s, 3H, C*H*_3_); *δ*_C_ (100 MHz, DMSO‐*d*_6_): 169.54, 136.74, 136.61, 131.45, 130.40, 128.31, 126.51, 20.43; ESI‐HRMS (*m/z*) [M‐H]^−^: calculated for C_8_H_10_N_2_O 150.0793; found 150.0801.

3‐Methylbenzohydrazide (**63**): Compound **63** was obtained according to the general procedure earlier reported with methyl 3‐methylbenzoate (**43**) as the starting material. Yield 10%; silica gel TLC R_*f*_ 0.25 (MeOH/DCM 5% *v/v*); δ_H_ (400 MHz, DMSO‐*d*_6_): 9.37 (s, 1H, exchange with D_2_O, N*H*), 7.26 (m, 4H, 4 x Ar‐*H*), 4.42 (s, 2H, exchange with D_2_O, N*H*_2_), 2.32 (s, 3H, C*H*_3_); *δ*_C_ (100 MHz, DMSO‐*d*_6_): 167.05, 138.60, 134.34, 132.66, 129.24, 128.65, 125.05, 22.02; ESI‐HRMS (*m/z*) [M‐H]^−^: calculated for C_8_H_10_N_2_O 150.0793; found 150.0797.

4‐Methylbenzohydrazide (**64**): Compound **64** was obtained according to the general procedure earlier reported, with methyl 4‐methylbenzoate (**44**) as the starting material. Yield 12%; silica gel TLC R_*f*_ 0.26 (MeOH/DCM 5% *v/v*); *δ*_H_ (400 MHz, DMSO‐*d*_6_): 9.69 (s, 1H, exchange with D_2_O, N*H*), 7.64 (s, 1H, Ar‐*H*), 7.60 (dd, *J* = 4.6 Hz, 1H, Ar‐*H*), 7.32 (d, *J* = 4.6 Hz, 2H, 2 x Ar‐*H*), 4.46 (s, 2H, exchange with D_2_O, N*H*_2_), 2.34 (s, 3H, C*H*_3_); *δ*_C_ (100 MHz, DMSO‐*d*_6_): 166.91, 141.93, 131.55, 129.88, 127.99, 22.00; ESI‐HRMS (*m/z*) [M‐H]^−^: calculated for C_8_H_10_N_2_O 150.0793; found 150.0785.

4‐Chlorobenzohydrazide (**65**): Compound **65** was obtained according to the general procedure earlier reported, with methyl 4‐chlorobenzoate (**45**) as the starting material. Yield 35%; silica gel TLC R_*f*_ 0.20 (MeOH/DCM 5% *v/v*); *δ*_H_ (400 MHz, DMSO‐*d*_6_): 9.84 (s, 1H, exchange with D_2_O, N*H*), 7.83 (d, *J* = 8.4 Hz, 2H, 2 x Ar‐*H*), 7.52 (d, *J* = 8.4 Hz, 2H, 2 x Ar‐*H*), 4.51 (s, 2H, exchange with D_2_O, N*H*_2_); *δ*_C_ (100 MHz, DMSO‐*d*_6_): 165.84, 136.93, 133.10, 129.92, 129.48; ESI‐HRMS (*m/z*) [M‐H]^−^: calculated for C_7_H_7_ClN_2_O 170.0247; found 170.0256.

3,4‐Dimethoxybenzohydrazide (**66**): Compound **66** was obtained according to the general procedure earlier reported, with methyl 3,4‐dimethoxybenzoate (**46**) as the starting material. Yield 22%; silica gel R_*f*_ 0.35 (MeOH/DCM 5% *v/v*); δ_H_ (400 MHz, DMSO‐*d*_6_): 9.62 (s, 1H, exchange with D_2_O, N*H*), 7.45 (m, 2H, 2 x Ar‐*H*), 7.00 (d, *J* = 8.2 Hz, 1H, Ar‐*H*), 4.42 (s, 2H, exchange with D_2_O, N*H*_2_), 3.79 (s, 6H, 2 x C*H*_3_); δ_C_ (100 MHz, DMSO‐*d*_6_): 166.66, 152.11, 149.25, 126.56, 121.17, 111.98, 111.27, 56.60, 56.54; ESI‐HRMS (*m/z*) [M‐H]^−^: calculated for C_9_H_12_N_2_O_3_ 196.0848; found 196.0843.

3‐(Dimethylamino)benzohydrazide (**67**): Compound **67** was obtained according to the general procedure earlier reported, with methyl 3‐(dimethylamino)benzoate (**47**) as the starting material. Yield 26%; silica gel TLC R_*f*_ 0.28 (MeOH/DCM 5% *v/v*); *δ*_H_ (400 MHz, DMSO‐*d*_6_): 10.28 (s, 1H, exchange with D_2_O, N*H*), 7.25 (dd, *J* = 7.8 Hz, 1H, Ar‐*H*), 7.09 (m, 2H, 2 x Ar‐*H*), 6.89 (d, *J* = 7.8 Hz, 1H, Ar‐*H*), 2.93 (s, 6H, 2 x C*H*_3_); *δ*_C_ (100 MHz, DMSO‐*d*_6_): 161.39, 151.28, 135.93, 129.86, 116.20, 116.06, 112.25, 26.14; ESI‐HRMS (*m/z*) [M‐H]^−^: calculated for C_9_H_12_N_3_O 179.1059; found 179.1064.

Pyridine‐3‐carbohydrazide (**68**): Compound (**68**) was obtained according to the general procedure earlier reported, with methyl pyridine‐3‐carboxylate (**48**) as the starting material. Yield 9%; silica gel TLC R_*f*_ 0.15 (MeOH/DCM 5% *v/v*); *δ*_H_ (400 MHz, DMSO‐*d*_6_): 9.95 (s, 1H, exchange with D_2_O, N*H*), 8.97 (s, 1H, Ar‐*H*), 8.69 (m, 1H, Ar‐*H*), 8.15 (d, *J* = 6.48 Hz, 1H, Ar‐*H*), 7.49 (m, 1H, Ar‐*H*), 4.55 (s, 2H, exchange with D_2_O, N*H*_2_); δ_C_ (100 MHz, DMSO‐*d*_6_): 165.37, 152.82, 149.14, 135.72, 129.97, 124.55; ESI‐HRMS (*m/z*) [M‐H]^−^: calculated for C_6_H_7_N_3_O 137.0589; found 137.0581.

*N*‐[4‐(Hydrazinecarbonyl)phenyl]benzamide (**69**): Compound **69** was obtained according to the general procedure earlier reported, with methyl 4‐benzamidobenzoate (**4**) as the starting material. Yield 39%; silica gel TLC R_*f*_ 0.31 (MeOH/DCM 5% *v/v*); *δ*_H_ (400 MHz, DMSO‐*d*_6_): 10.42 (s, 1H, exchange with D_2_O, N*H)*, 9.67 (s, 1H, N*H*), 7.96 (d, 2H, *J* = 7.2 Hz, 2 x Ar‐*H*), 7.85 (d, 2H, *J* = 9.0 Hz, 2 x Ar‐*H*), 7.82 (d, 2H, *J* = 9.0 Hz, 2 x Ar‐*H*), 7.61 (dd, *J* = 7.2 Hz, 1H, Ar‐*H*), 7.54 (dd, *J* = 7.2 Hz, 2H, 2 x Ar‐*H*), 4.48 (s, 2H, exchange with D_2_O, N*H*_2_); δ_C_ (100 MHz, DMSO‐*d*_6_): 166.89, 166.58, 142.76, 135.81, 132.84, 129.51, 129.24, 128.80, 128.69, 120.52; ESI‐HRMS (*m/z*) [M‐H]^−^: calculated for C_14_H_13_N_3_O_2_ 255.1008; found 255.1017.

*N*‐[4‐(Hydrazinecarbonyl)phenyl]‐4‐nitrobenzamide (**70**): Compound **70** was obtained according to the general procedure earlier reported, with methyl 4‐(4‐nitrobenzamido)benzoate (**5**) as the starting material. Yield 40%; silica gel TLC R_*f*_ 0.35 (MeOH/DCM 5% *v/v*); *δ*_H_ (400 MHz, DMSO‐*d*_6_): 10.78 (s, 1H, exchange with D_2_O, N*H*), 9.74 (s, 1H, exchange with D_2_O, N*H*), 8.43 (d, *J* = 8.7 Hz, 2H, 2 x Ar‐*H*), 8.24 (d, *J* = 8.7 Hz, 2H, 2 x Ar‐*H*), 7.89 (m, 4H, 4 x Ar‐*H*), 4.50 (s, 2H, 2 x Ar‐*H*), 4.64 (s, 2H, exchange with D_2_O, N*H*_2_); δ_C_ (100 MHz, DMSO‐*d*_6_): 166.49, 165.25, 150.31, 142.25, 141.43, 130.38, 129.75, 128.78, 124.67, 120.72; ESI‐HRMS (*m/z*) [M‐H]^−^: calculated for C_14_H_12_N_4_O_4_ 300.0859; found 300.0868.

1‐(4‐Fluorophenyl)‐3‐[4‐(hydrazinecarbonyl)phenyl]urea (**71**): Compound **71** was obtained according to the general procedure earlier reported, with methyl 4‐[3‐(4‐fluorophenyl)ureido]benzoate (**15**) as the starting material. Yield 73%; silica gel TLC R_*f*_ 0.19 (MeOH/DCM 5% *v/v*); *δ*_H_ (400 MHz, DMSO‐*d*_6_): 9.60 (s, 1H, exchange with D_2_O, N*H*), 8.90 (s, 1H, exchange with D_2_O, N*H*CONH), 8.79 (s, 1H, exchange with D_2_O, NHCON*H*), 7.76 (d, *J* = 8.8 Hz, 2H, 2 x Ar‐*H*), 7.48 (m, 4H, 4 x Ar‐*H*), 7.13 (m, 2H, 2 x Ar‐*H*), 4.42 (s, 2H, exchange with D_2_O, N*H*_2_); *δ*_C_ (100 MHz, DMSO‐*d*_6_): 166.76, 159.73 (s, *J* ^1^_C‐F_ = 243.7 Hz), 153.49, 143.41, 136.85 (s, *J* ^4^_C‐F_ = 2.3 Hz), 128.97, 127.45, 121.26 (s, *J* ^3^_C‐F_ = 7.6 Hz), 118.34, 116.48 (s, *J* ^2^_C‐F_ = 22.2 Hz); *δ*_F_ (376 MHz, DMSO‐*d*_6_): –121.12; ESI‐HRMS (*m/z*) [M‐H]^−^: calculated for C_14_H_13_FN_4_O_2_ 288.1023; found 288.1031.

1‐(4‐Chlorophenyl)‐3‐[4‐(hydrazinecarbonyl)phenyl]urea (**72**): Compound **72** was obtained according to the general procedure earlier reported, with methyl 4‐[3‐(4‐chlorophenyl)ureido]benzoate (**16**) as the starting material. Yield 50%; silica gel TLC R_*f*_ 0.26 (MeOH/DCM 5% *v/v*); *δ*_H_ (400 MHz, DMSO‐*d*_6_): 9.59 (s, 1H, exchange with D_2_O, N*H*), 8.92 (s, 1H, exchange with D_2_O, N*H*CONH), 8.88 (s, 1H, exchange with D_2_O, NHCON*H*), 7.77 (d, *J* = 8.6 Hz, 2H, 2 x Ar‐*H*), 7.49 (m, 4H, 4 x Ar‐*H*), 7.33 (d, *J* = 8.6 Hz, 2H, 2 x Ar‐*H*), 4.40 (s, 2H, exchange with D_2_O, N*H*_2_); δ_C_ (100 MHz, DMSO‐*d*_6_): 166.71, 153.30, 143.24, 139.54, 129.73, 128.97, 127.56, 126.64, 120.91, 118.39; ESI‐HRMS (*m/z*) [M‐H]^−^: calculated for C_14_H_13_ClN_4_O_2_ 304.0727; found 304.0720.

1‐[4‐(Hydrazinecarbonyl)phenyl]‐3‐[4‐(trifluoromethoxy)phenyl]urea (**73**): Compound **73** was obtained according to the general procedure earlier reported, with methyl 4‐{3‐[4‐(trifluoromethoxy)phenyl]ureido}benzoate (**17**) as the starting material. Yield 33%; silica gel TLC R_*f*_ 0.22 (MeOH/DCM 5% *v/v*); *δ*_H_ (400 MHz, DMSO‐*d*_6_): 9.62 (t, *J* = 3.3 Hz, 1H, exchange with D_2_O, N*H*), 8.96 (bs, 2H, exchange with D_2_O, N*H*CON*H*), 7.77 (d, *J* = 8.8 Hz, 2H, 2 x Ar‐*H*), 7.56 (d, *J* = 8.8 Hz, 2H, 2 x Ar‐*H*), 7.51 (d, *J* = 8.6 Hz, 2H, 2 x Ar‐*H*), 7.29 (d, *J* = 8.6 Hz, 2H, Ar‐*H*), 4.43 (d, *J* = 3.3 Hz, 2H, exchange with D_2_O, N*H*_2_); *δ*_C_ (100 MHz, DMSO‐*d*_6_): 166.68, 153.34, 143.88, 143.22, 139.85, 128.96, 127.59, 125.07 (q, *J* ^1^_C‐F_ = 250.4 Hz), 122.84, 120.59, 118.41; δ_F_ (376 MHz, DMSO‐*d*_6_): −57.10; ESI‐HRMS (*m/z*) [M‐H]^−^: calculated for C_15_H_13_F_3_N_4_O_3_ 354.0940; found 354.0932.

1‐(Furan‐2‐ylmethyl)‐3‐[4‐(hydrazinecarbonyl)phenyl]urea (**74**): Compound **74** was obtained according to the general procedure earlier reported with methyl 4‐[3‐(furan‐2‐ylmethyl)ureido]benzoate (**18**) as starting material. Yield 80%; silica gel TLC R_*f*_ 0.22 (MeOH/DCM 5% *v/v*); *δ*_H_ (400 MHz, DMSO‐*d*_6_): 9.55 (s, 1H, exchange with D_2_O, N*H*), 8.77 (s, 1H, exchange with D_2_O, N*H*CONH), 7.71 (d, *J* = 8.7 Hz, 2H, 2 x Ar‐*H*), 7.58 (s, 1H, Ar‐*H*), 7.43 (d, *J* = 8.7 Hz, 2H, 2 x Ar‐*H*), 6.65 (t, *J* = 5.6 Hz, 1H, exchange with D_2_O, NHCON*H*), 6.39 (dd, J = 2.5 Hz, 1H, Ar‐*H*), 6.26 (d, *J* = 2.5 Hz, 1H, Ar‐*H*), 4.39 (s, 2H, exchange with D_2_O, N*H*_2_), 4.29 (d, *J* = 5.6 Hz, 2H, C*H*_2_); δ_C_ (100 MHz, DMSO‐*d*_6_): 166.81, 155.74, 154.01, 144.05, 143.13, 128.89, 126.80, 117.77, 111.53, 107.62, 37.17; ESI‐HRMS (*m/z*) [M‐H]^−^: calculated for C_13_H_14_N_4_O_3_ 274.1066; found 274.1075.

1‐Benzyl‐3‐[4‐(hydrazinecarbonyl)phenyl]urea (**75**): Compound **75** was obtained according to the general procedure earlier reported, with methyl 4‐(3‐benzylureido)benzoate (**19**) as the starting material. Yield 50%; silica gel TLC R_*f*_ 0.30 (MeOH/DCM 5% *v/v*); *δ*_H_ (400 MHz, DMSO‐*d*_6_): 9.55 (s, 1H, exchange with D_2_O, N*H*), 8.81 (s, 1H, exchange with D_2_O, N*H*CONH), 7.71 (d, *J* = 8.7 Hz, 2H, 2 x Ar‐*H*), 7.45 (d, *J* = 8.7 Hz, 2H, 2 x Ar‐*H*), 7.32 (m, 4H, 4 x Ar‐*H*), 7.25 (m, 1H, Ar‐*H*), 6.72 (t, *J* = 5.9 Hz, 1H, exchange with D_2_O, NHCON*H*), 4.39 (s, 2H, exchange with D_2_O, N*H*_2_), 4.30 (d, *J* = 5.9 Hz, 2H, C*H*_2_); *δ*_C_ (100 MHz, DMSO‐*d*_6_): 166.81, 156.02, 144.17, 141.22, 129.38, 128.89, 128.20, 127.83, 126.71, 117.75, 43.81; ESI‐HRMS (*m/z*) [M‐H]^−^: calculated for C_15_H_16_N_4_O_2_ 284.1273; found 284.1280.

1‐[4‐(Hydrazinecarbonyl)phenyl]‐3‐(4‐phenoxyphenyl)urea (**76**): Compound **76** was obtained according to the general procedure earlier reported, with methyl 4‐[3‐(4‐phenoxyphenyl)ureido]benzoate (**20**) as the starting material. Yield 80%; silica gel TLC R_*f*_ 0.19 (MeOH/DCM 5% *v/v*); *δ*_H_ (400 MHz, DMSO‐*d*_6_): 9.60 (s, 1H, exchange with D_2_O, N*H*), 8.89 (s, 1H, exchange with D_2_O, N*H*CONH), 8.76 (s, 1H, exchange with D_2_O, NHCON*H*), 7.77 (d, *J* = 8.6 Hz, 2H, 2 x Ar‐*H*), 7.49 (m, 4H, 4 x Ar‐*H*), 7.36 (dd, *J* = 7.5 Hz, 2H, 2 x Ar‐*H*), 7.08 (dd, *J* = 7.5 Hz, 1H, Ar‐*H*), 6.97 (m, 4H, 4 x Ar‐*H*), 4.42 (s, 2H, exchange with D_2_O, N*H*_2_); δ_C_ (100 MHz, DMSO‐*d*_6_): 166.74, 158.66, 153.47, 151.93, 143.46, 136.49, 130.98, 128.97, 127.38, 123.87, 121.17, 120.83, 118.72, 118.27; ESI‐HRMS (*m/z*) [M‐H]^−^: calculated for C_20_H_18_N_4_O_3_ 362.1379; found 362.1371.

1‐[4‐(Hydrazinecarbonyl)phenyl]‐3‐(3‐methoxyphenyl)urea (**77**): Compound **77** was obtained according to the general procedure earlier reported, with methyl 4‐[3‐(3‐methoxyphenyl)ureido]benzoate (**21**) as the starting material. Yield 40%; silica gel TLC R_*f*_ 0.33 (MeOH/DCM 5% *v/v*); δ_H_ (400 MHz, DMSO‐*d*_6_): 9.60 (s, 1H, exchange with D_2_O, N*H*), 8.88 (s, 1H, exchange with D_2_O, N*H*CONH), 8.75 (s, 1H, exchange with D_2_O, NHCON*H*), 7.76 (d, *J* = 8.5 Hz, 2H, 2 x Ar‐*H*), 7.50 (d, *J* = 8.5 Hz, 2H, 2 x Ar‐*H*), 7.18 (m, 2H, 2 x Ar‐*H*), 6.93 (d, *J* = 8.0 Hz, 1H, 1 x Ar‐*H*), 6.57 (d, *J* = 8.0 Hz, 1H, 1 x Ar‐*H*), 4.41 (s, 2H, exchange with D_2_O, N*H*_2_), 3.72 (s, 3H, C*H*_3_); δ_C_ (100 MHz, DMSO‐*d*_6_): 166.72, 160.75, 153.30, 143.35, 141.73, 130.65, 128.97, 127.44, 118.30, 111.67, 108.51, 105.12, 55.99; ESI‐HRMS (*m/z*) [M‐H]^−^: calculated for C_15_H_16_N_4_O_3_ 300.1222; found 300.1228.

1‐(2,3‐Dihydrobenzo[*b*][1,4]dioxin‐6‐yl)‐3‐[4‐(hydrazinecarbonyl)phenyl]urea (**78**): Compound **78** was obtained according to the general procedure earlier reported, with methyl 4‐[3‐(2,3‐dihydrobenzo[b][1,4]dioxin‐6‐yl)ureido]benzoate (**22**) as the starting material. Yield 95%; silica gel TLC R_*f*_ 0.20 (MeOH/DCM 5% *v/v*); δ_H_ (400 MHz, DMSO‐*d*_6_): 9.58 (s, 1H, exchange with D_2_O, N*H*), 8.80 (s, 1H, exchange with D_2_O, N*H*CONH), 8.54 (s, 1H, exchange with D_2_O, NHCON*H*), 7.75 (d, *J* = 8.5 Hz, 2H, 2 x Ar‐*H*), 7.48 (d, *J* = 8.5 Hz, 2H, 2 x Ar‐*H*), 7.90 (s, 1H, Ar‐*H*), 6.78 (m, 2H, 2 x Ar‐*H*), 4.41 (s, 2H, exchange with D_2_O, N*H*_2_), 4.20 (m, 4H, 2 x C*H*_2_); δ_C_ (100 MHz, DMSO‐*d*_6_): 166.76, 153.43, 144.17, 143.43, 139.67, 134.11, 128.95, 127.26, 118.20, 117.93, 112.84, 108.72, 65.28, 64.94; ESI‐HRMS (*m/z*) [M‐H]^−^: calculated for C_16_H_16_N_4_O_4_ 328.1172; found 328.1181.

1‐(4‐Fluorophenyl)‐3‐[3‐(hydrazinecarbonyl)phenyl]urea (**79**): Compound **79** was obtained according to the general procedure earlier reported, with methyl 3‐[3‐(4‐fluorophenyl)ureido]benzoate (**23**) as the starting material. Yield 93%; silica gel TLC R_*f*_ 0.22 (MeOH/DCM 5% *v/v*); δ_H_ (400 MHz, DMSO‐*d*_6_): 9.67 (s, 1H, exchange with D_2_O, N*H*), 8.76 (s, 1H, exchange with D_2_O, N*H*CONH), 8.69 (s, 1H, exchange with D_2_O, NHCON*H*), 7.84 (s, 1H, Ar‐H), 7.60 (d, *J* = 7.6 Hz, 1H, Ar‐*H*), 7.47 (m, 2H, 2 x Ar‐*H*), 7.39 (d, *J* = 7.6 Hz, 1H, Ar‐*H*), 7.33 (dd, *J* = 7.8 Hz, 1H, Ar‐*H*), 7.12 (m, 2H, 2 x Ar‐*H*), 4.46 (s, 2H, exchange with D_2_O, N*H*_2_); *δ*_C_ (100 MHz, DMSO‐*d*_6_): 167.02, 159.83 (s, *J* ^1^_C‐F_ = 241.9 Hz), 153.66, 140.84, 137.00, 135.17, 129.76, 121.82, 121.17 (s, *J* ^3^_C‐F_ = 8.4 Hz), 118.39, 116.46 (s, *J* ^2^_C‐F_ = 23.1 Hz); δ_F_ (376 MHz, DMSO‐*d*_6_): −121.36; ESI‐HRMS (*m/z*) [M‐H]^−^: calculated for C_14_H_13_FN_4_O_2_ 288.1023; found 288.1029.

1‐(4‐Chlorophenyl)‐3‐[3‐(hydrazinecarbonyl)phenyl]urea (**80**): Compound **80** was obtained according to the general procedure earlier reported, with methyl 3‐[3‐(4‐chlorophenyl)ureido]benzoate (**24**) as the starting material. Yield 50%; silica gel TLC R_*f*_ 0.27 (MeOH/DCM 5% *v/v*); δ_H_ (400 MHz, DMSO‐*d*_6_): 9.72 (s, 1H, exchange with D_2_O, N*H*), 8.84 (bs, 2H, exchange with D_2_O, N*H*CON*H*), 7.85 (s, 1H, Ar‐*H*), 7.60 (d, *J* = 7.8 Hz, 1H, Ar‐*H*), 7.49 (d, *J* = 8.8 Hz, 2H, 2 x Ar‐*H*), 7.40 (d, *J* = 7.8 Hz, 1H, Ar‐*H*), 7.33 (m, 3H, 3 X Ar‐*H*), 4.47 (s, 2H, exchange with D_2_O, N*H*_2_); *δ*_C_ (100 MHz, DMSO‐*d*_6_): 166.99, 153.47, 140.69, 139.68, 135.18, 129.78, 129.69, 126.50, 121.90, 121.25, 120.88, 118.47; ESI‐HRMS (*m/z*) [M‐H]^−^: calculated for C_14_H_13_ClN_4_O_2_ 304.0727; found 304.0722.

1‐[3‐(Hydrazinecarbonyl)phenyl]‐3‐[4‐(trifluoromethoxy)phenyl]urea (**81**): Compound **81** was obtained according to the general procedure earlier reported, with methyl 3‐{3‐[4‐(trifluoromethoxy)phenyl]ureido}benzoate (**25**) as the starting material. Yield 50%; silica gel TLC R_*f*_ 0.25 (MeOH/DCM 5% *v/v*); *δ*_H_ (400 MHz, DMSO‐*d*_6_): 9.71 (s, 1H, exchange with D_2_O, N*H*), 8.92 (s, 1H, exchange with D_2_O, N*H*CONH), 8.87 (s, 1H, exchange with D_2_O, NHCON*H*), 7.86 (s, 1H, Ar‐*H*), 7.61 (d, *J* = 7.7 Hz, 1H, Ar‐*H*), 7.56 (d, *J* = 8.9 Hz, 2H, 2 x Ar‐*H*), 7.40 (d, *J* = 7.7 Hz, 1H, Ar‐*H*), 7.34 (dd, *J* = 7.7 Hz, 1H, Ar‐*H*), 7.29 (d, *J* = 8.9 Hz, 2H, 2 x Ar‐*H*), 4.47 (s, 2H, exchange with D_2_O, N*H*_2_); *δ*_C_ (100 MHz, DMSO‐*d*_6_): 167.00, 153.53, 143.80, 140.69, 140.00, 135.18, 129.79, 124.30 (q, *J* ^1^_C‐F_ = 286.2 Hz), 122.81, 121.92, 121.27, 120.53, 118.48; δ_F_ (376 MHz, DMSO‐*d*_6_): −57.10; ESI‐HRMS (*m/z*) [M‐H]^−^: calculated for C_15_H_13_F_3_N_4_O_3_ 354.0940; found 354.0947.

1‐(Furan‐2‐ylmethyl)‐3‐[3‐(hydrazinecarbonyl)phenyl]urea (**82**): Compound **82** was obtained according to the general procedure earlier reported, with methyl 3‐[3‐(furan‐2‐ylmethyl)ureido]benzoate (**26**) as the starting material. Yield 40%; silica gel TLC R_*f*_ 0.23 (MeOH/DCM 5% *v/v*); *δ*_H_ (400 MHz, DMSO‐*d*_6_): 0.66 (s, 1H, exchange with D_2_O, N*H*), 8.64 (s, 1H, exchange with D_2_O, N*H*CONH), 7.77 (s, 1H, Ar‐*H*), 7.57 (d, *J* = 7.6 Hz), 7.29 (m, 2H, 2 x Ar‐*H*), 6.58 (t, *J* = 5.5 Hz, 1H, exchange with D_2_O, NHCON*H*), 6.39 (m, 1H, Ar‐*H*), 6.26 (d, *J* = 2.9 Hz, 1H, Ar‐*H*), 4.45 (s, 2H, exchange with D_2_O, N*H*_2_), 4.29 (d, *J* = 5.5 Hz, 2H, C*H*_2_); δ_C_ (100 MHz, DMSO‐*d*_6_): 167.14, 155.89, 154.12, 143.12, 141.45, 135.08, 129.65, 121.27, 120.48, 117.85, 111.53, 107.59; ESI‐HRMS (*m/z*) [M‐H]^−^: calculated for C_13_H_14_N_4_O_3_ 274.1066; found 274.1071.

1‐Benzyl‐3‐[3‐(hydrazinecarbonyl)phenyl]urea (**83**): Compound **83** was obtained according to the general procedure earlier reported, with methyl 3‐(3‐benzylureido)benzoate (**27**) as the starting material. Yield 45%; silica gel TLC R_*f*_ 0.29 (MeOH/DCM 5% *v/v*); δ_H_ (400 MHz, DMSO‐*d*_6_): 9.65 (s, 1H, exchange with D_2_O, N*H*), 8.68 (s, 1H, exchange with D_2_O, N*H*CONH), 7.79 (s, 1H, Ar‐*H*), 7.58 (d, *J* = 7.8 Hz, 1H, Ar‐*H*), 7.29 (m, 7H, 7 x Ar‐*H*), 6.64 (t, *J* = 5.9 Hz, 1H, exchange with D_2_O, NHCON*H*), 4.44 (s, 2H, exchange with D_2_O, N*H*_2_), 4.30 (d, *J* = 5.9 Hz, 2H, C*H*_2_); δ_C_ (100 MHz, DMSO‐*d*_6_): 167.18, 156.21, 141.61, 141.37, 135.07, 129.63 129.37, 128.19, 127.80, 121.25, 120.37, 117.85; ESI‐HRMS (*m/z*) [M‐H]^−^: calculated for C_15_H_16_N_4_O_2_ 284.1273; found 284.1268.

1‐[3‐(Hydrazinecarbonyl)phenyl]‐3‐(4‐phenoxyphenyl)urea (**84**): Compound **84** was obtained according to the general procedure earlier reported, with methyl 3‐[3‐(4‐phenoxyphenyl)ureido]benzoate (**28**) as the starting material. Yield 70%; silica gel TLC R_*f*_ 0.22 (MeOH/DCM 5% *v/v*); *δ*_H_ (400 MHz, DMSO‐*d*_6_): 9.71 (s, 1H, exchange with D_2_O, N*H*), 8.78 (s, 1H, exchange with D_2_O, CON*H*‐CONH), 8.70 (s, 1H, exchange with D_2_O, CON*H*‐CONH), 7.86 (s, 1H, Ar‐*H*), 7.62 (d, *J* = 8.1 Hz, 1H, Ar‐*H*), 7.48 (d, *J* = 8.9 Hz, 2H, 2 x Ar‐*H*), 7.36 (m, 4H, 4 x Ar‐*H*), 7.09 (dd, *J* = 7.3 Hz, 1H, Ar‐*H*), 6.97 (m, 4H, 4 x Ar‐*H*), 4.48 (s, 2H, exchange with D_2_O, N*H*_2_); δ_C_ (100 MHz, DMSO‐*d*_6_): 167.06, 158.70, 153.65, 151.80, 140.91, 136.65, 135.17, 130.99, 129.78, 123.85, 121.78, 121.11, 120.84, 118.70, 118.33; ESI‐HRMS (*m/z*) [M‐H]^−^: calculated for C_20_H_18_N_4_O_3_ 362.1379; found 362.1373.

1‐[3‐(Hydrazinecarbonyl)phenyl]‐3‐(3‐methoxyphenyl)urea (**85**): Compound **85** was obtained according to the general procedure earlier reported, with methyl 3‐[3‐(3‐methoxyphenyl)ureido]benzoate (**29**) as the starting material. Yield 55%; silica gel TLC R_*f*_ 0.35 (MeOH/DCM 5% *v/v*); δ_H_ (400 MHz, DMSO‐*d*_6_): 9.72 (s, 1H, exchange with D_2_O, N*H*), 8.78 (s, 1H, exchange with D_2_O, N*H*CONH), 8.70 (s, 1H, exchange with D_2_O, NHCON*H*), 8.19 (s, 1H, Ar‐*H*), 7.87 (s, 1H, Ar‐*H*), 7.60 (d, *J* = 7.9 Hz, 1H, Ar‐*H*), 7.40 (d, *J* = 7.9 Hz, 1H, Ar‐*H*), 7.33 (dd, *J* = 7.9 Hz, 1H, Ar‐*H*), 7.18 (m, 2H, 2 x Ar‐*H*), 6.94 (d, *J* = 7.9 Hz, 1H, Ar‐*H*), 6.56 (d, *J* = 8.2 Hz, 1H, Ar‐*H*), 4.47 (s, 2H, exchange with D_2_O, N*H*_2_), 3.73 (s, 3H, C*H*_3_); *δ*_C_ (100 MHz, DMSO‐*d*_6_): 167.07, 160.76, 153.50, 141.89, 140.80, 135.18, 130.62, 129.78, 121.84, 121.17, 118.35, 111.64, 108.44, 105.07, 55.99; ESI‐HRMS (*m/z*) [M‐H]^−^: calculated for C_15_H_16_N_4_O_3_ 300.1222; found 300.1215.

1‐(2,3‐Dihydrobenzo[*b*][1,4]dioxin‐6‐yl)‐3‐[3‐(hydrazinecarbonyl)phenyl]urea (**86**): Compound **86** was obtained according to the general procedure earlier reported, with methyl 3‐[3‐(2,3‐dihydrobenzo[b][1,4]dioxin‐6‐yl)ureido]benzoate (**30**) as the starting material. Yield 80%; silica gel TLC R_*f*_ 0.21 (MeOH/DCM 5% *v/v*); δ_H_ (400 MHz, DMSO‐*d*_6_): 9.69 (s, 1H, exchange with D_2_O, N*H*), 8.97 (s, 1H, exchange with D_2_O, N*H*CONH), 8.76 (s, 1H, exchange with D_2_O, NHCON*H*), 7.85 (s, 1H, Ar‐*H*), 7.60 (d, *J* = 8.5 Hz, 1H, Ar‐*H*), 7.36 (d, *J* = 7.7 Hz, 1H, Ar‐*H*), 7.30 (dd, *J* = 7.7 Hz, 1H, Ar‐*H*), 7.11 (d, *J* = 2.3 Hz, 1H, Ar‐*H*), 6.81 (d, *J* = 8.5 Hz, 1H, Ar‐*H*), 6.75 (d, *J* = 8.7 Hz, 1H, Ar‐*H*), 4.20 (m, 6H, exchange with D_2_O, 2 x C*H*_2_ + N*H*_2_); *δ*_C_ (100 MHz, DMSO‐*d*_6_): 167.09, 153.60, 144.16, 140.97, 139.57, 135.15, 134.26, 129.74, 121.72, 120.97, 118.25, 117.90, 112.78, 108.66, 65.28, 64.94; ESI‐HRMS (*m/z*) [M‐H]^−^: calculated for C_16_H_16_N_4_O_4_ 328.1172; found 328.1177.

### 4.2 Carbonic Anhydrase Inhibition

An Applied Photophysics stopped‐flow instrument has been used for assaying the CA‐catalyzed CO_2_ hydration activity [31]. Phenol red (at a concentration of 0.2 mM) has been used as an indicator, working at the absorbance maximum of 557 nm, with 20 mM Hepes (pH 7.5) as a buffer and 20 mM Na_2_SO_4_ (for maintaining constant the ionic strength), following the initial rates of the CA‐catalyzed CO_2_ hydration reaction for a period of 10–100 s. The CO_2_ concentrations ranged from 1.7 to 17 mM for the determination of the kinetic *para*meters and inhibition constants. For each inhibitor, at least six traces of the initial 5%–10% of the reaction have been used to determine the initial velocity. The uncatalysed rates were determined in the same manner and subtracted from the total observed rates. Stock solutions of inhibitor (0.1 mM) were prepared in distilled‐deionized water and dilutions up to 0.01 nM were done thereafter with the assay buffer. Inhibitor and enzyme solutions were preincubated together for 30 min at room temperature before assay to allow for the formation of the E‐I complex. The inhibition constants were obtained by nonlinear least‐squares methods using PRISM 3 and the Cheng‐Prusoff equation, as reported earlier [24], and represent the mean from at least three different determinations. The enzyme concentrations were in the range 6–14 nM. All hCA isoforms were recombinant ones obtained in‐house, as reported earlier [26].

### 4.3 In Silico Studies

The crystal structures of hCA I (PDB: 2NMX) [32], hCA II (PDB: 6RM1) [29], hCA IV (PDB: 1ZNC) [33], CA IX (PDB: 5FL4) [34], and CA XII (PDB: 1JD0) [35] employed in computational studies were obtained from the Protein Data Bank [36]. These structures were then processed using the Protein Pre*para*tion module in the Maestro Schrödinger suite [37], assigning bond orders, adding hydrogens, deleting water molecules, and optimizing H‐bonding networks. Subsequently, energy minimization was performed with a root means square deviation (RMSD) value of 0.30, applying the Optimized Potential for Liquid Simulation (OPLS4) force field [37, 38, 39, 40, 41]. The 3D ligand structures were prepared by Maestro[37a] and evaluated for their ionization states at pH 7.3 ± 1.0 with Epik.[37b] The conjugate gradient method in Macromodel[37c] was used for energy minimization (maximum iteration number: 2500; convergence criterion: 0.05 Kcal/mol/Å^2^). Grids for docking were centered in the centroid of the complexed ligand. Docking studies were carried out with the program Glide[37d] using the standard precision (SP) mode. Figures were generated with Maestro and Chimera [37, 42].

## Supporting information

Supporting information.

Supporting information.

## Data Availability

Data that support the findings of this study are available from the corresponding author upon reasonable request.
