# Supplementary material for: Exploration of Aromatic Hydrazides as Inhibitors of Human Carbonic Anhydrases
Source: Arch Pharm (Weinheim). 2025 Apr 1;358(4):e202400963. doi: 10.1002/ardp.202400963 (PMC11959329; doi:10.1002/ardp.202400963)
Supplement: Supplementary file 1 — Supporting information. [file ARDP-358-e202400963-s002.doc]

**Supplemental Material: Novel Compounds and Biological Screening Results**

**Exploration of aromatic hydrazides as inhibitors of human carbonic anhydrases**

German Benito Menendez,^1^ Simone Giovannuzzi,^1^,* Alessandro Bonardi,^1,2^,* Alessio Nocentini,^1,2^ Paola Gratteri,^1,2^ Claudiu T. Supuran.^1^

1 NEUROFARBA Department, Pharmaceutical and Nutraceutical Section, University of Florence, 50019, Sesto Fiorentino, Florence, Italy

2 NEUROFARBA Department, Pharmaceutical and Nutraceutical Section, Laboratory of Molecular Modeling Cheminformatics & QSAR, University of Florence, Via U. Schiff 6, 50019, Sesto Fiorentino, Florence, Italy

[Simone.giovannuzzi@unifi.it](mailto:Simone.giovannuzzi@unifi.it); [alessandro.bonardi@unifi.it](mailto:alessandro.bonardi@unifi.it)

| Cmp | IncCode | K_I_ (μM)^a^ | | | | |
| --- | --- | --- | --- | --- | --- | --- |
|  |  | CA I | CA II | CA IV | CA IX | CA XII |
| **49** | InChI=1S/C7H7N3O3/c8-9-7(11)5-1-3-6(4-2-5)10(12)13/h1-4H,8H2,(H,9,11) | >100 | >100 | >100 | >100 | >100 |
| **50** | InChI=1S/C7H7N3O3/c8-9-7(11)5-2-1-3-6(4-5)10(12)13/h1-4H,8H2,(H,9,11) | >100 | >100 | >100 | >100 | >100 |
| **51** | InChI=1S/C7H9N3O/c8-6-3-1-5(2-4-6)7(11)10-9/h1-4H,8-9H2,(H,10,11) | >100 | >100 | >100 | >100 | >100 |
| **52** | InChI=1S/C7H9N3O/c8-6-3-1-2-5(4-6)7(11)10-9/h1-4H,8-9H2,(H,10,11) | >100 | 77.1 | >100 | >100 | >100 |
| **53** | InChI=1S/C9H9N3O/c10-12-9(13)7-5-11-8-4-2-1-3-6(7)8/h1-5,11H,10H2,(H,12,13) | >100 | >100 | >100 | >100 | >100 |
| **54** | InChI=1S/C9H9N3O/c10-12-9(13)8-5-6-3-1-2-4-7(6)11-8/h1-5,11H,10H2,(H,12,13) | >100 | >100 | >100 | >100 | >100 |
| **55** | InChI=1S/C8H8N4O/c9-10-8(13)7-5-3-1-2-4-6(5)11-12-7/h1-4H,9H2,(H,10,13)(H,11,12) | >100 | 79.2 | >100 | >100 | >100 |
| **56** | InChI=1S/C9H8N2O2/c10-11-9(12)7-1-2-8-6(5-7)3-4-13-8/h1-5H,10H2,(H,11,12) | >100 | >100 | >100 | >100 | >100 |
| **57** | InChI=1S/C11H10N2O/c12-13-11(14)10-6-5-8-3-1-2-4-9(8)7-10/h1-7H,12H2,(H,13,14) | >100 | >100 | >100 | >100 | >100 |
| **58** | InChI=1S/C11H10N2O/c12-13-11(14)10-7-3-5-8-4-1-2-6-9(8)10/h1-7H,12H2,(H,13,14) | >100 | >100 | >100 | >100 | >100 |
| **59** | InChI=1S/C11H16N2O3/c1-3-15-9-5-8(11(14)13-12)6-10(7-9)16-4-2/h5-7H,3-4,12H2,1-2H3,(H,13,14) | >100 | >100 | >100 | >100 | >100 |
| **60** | InChI=1S/C10H8N2O3/c11-12-10(14)9-5-7(13)6-3-1-2-4-8(6)15-9/h1-5H,11H2,(H,12,14) | >100 | >100 | >100 | >100 | >100 |
| **61** | InChI=1S/C5H5BrN4O/c6-3-1-8-4(9-2-3)5(11)10-7/h1-2H,7H2,(H,10,11) | >100 | >100 | >100 | >100 | >100 |
| **62** | InChI=1S/C8H10N2O/c1-6-4-2-3-5-7(6)8(11)10-9/h2-5H,9H2,1H3,(H,10,11) | >100 | >100 | >100 | >100 | >100 |
| **63** | InChI=1S/C8H10N2O/c1-6-3-2-4-7(5-6)8(11)10-9/h2-5H,9H2,1H3,(H,10,11) | >100 | >100 | >100 | >100 | >100 |
| **64** | InChI=1S/C8H10N2O/c1-6-2-4-7(5-3-6)8(11)10-9/h2-5H,9H2,1H3,(H,10,11) | >100 | >100 | >100 | >100 | >100 |
| **65** | InChI=1S/C7H7ClN2O/c8-6-3-1-5(2-4-6)7(11)10-9/h1-4H,9H2,(H,10,11) | >100 | 93.5 | >100 | >100 | >100 |
| **66** | InChI=1S/C9H12N2O3/c1-13-7-4-3-6(9(12)11-10)5-8(7)14-2/h3-5H,10H2,1-2H3,(H,11,12) | >100 | >100 | >100 | >100 | >100 |
| **67** | InChI=1S/C9H13N3O/c1-12(2)8-5-3-4-7(6-8)9(13)11-10/h3-6H,10H2,1-2H3,(H,11,13) | >100 | 38.8 | >100 | >100 | >100 |
| **68** | InChI=1S/C6H7N3O/c7-9-6(10)5-2-1-3-8-4-5/h1-4H,7H2,(H,9,10) | >100 | >100 | >100 | >100 | >100 |
| **69** | InChI=1S/C14H13N3O2/c15-17-14(19)11-6-8-12(9-7-11)16-13(18)10-4-2-1-3-5-10/h1-9H,15H2,(H,16,18)(H,17,19) | >100 | 71.5 | >100 | 68.4 | 45.4 |
| **70** | InChI=1S/C14H12N4O4/c15-17-14(20)10-1-5-11(6-2-10)16-13(19)9-3-7-12(8-4-9)18(21)22/h1-8H,15H2,(H,16,19)(H,17,20) | >100 | 69.5 | 79.5 | 22.0 | 36.6 |
| **71** | InChI=1S/C14H13FN4O2/c15-10-3-7-12(8-4-10)18-14(21)17-11-5-1-9(2-6-11)13(20)19-16/h1-8H,16H2,(H,19,20)(H2,17,18,21) | 88.5 | 88.6 | >100 | 2.3 | 20.6 |
| **72** | InChI=1S/C14H13ClN4O2/c15-10-3-7-12(8-4-10)18-14(21)17-11-5-1-9(2-6-11)13(20)19-16/h1-8H,16H2,(H,19,20)(H2,17,18,21) | >100 | 56.8 | >100 | 29.2 | 61.2 |
| **73** | InChI=1S/C15H13F3N4O3/c16-15(17,18)25-12-7-5-11(6-8-12)21-14(24)20-10-3-1-9(2-4-10)13(23)22-19/h1-8H,19H2,(H,22,23)(H2,20,21,24) | >100 | 88.2 | >100 | 36.7 | 64.7 |
| **74** | InChI=1S/C13H14N4O3/c14-17-12(18)9-3-5-10(6-4-9)16-13(19)15-8-11-2-1-7-20-11/h1-7H,8,14H2,(H,17,18)(H2,15,16,19) | >100 | >100 | 6.1 | 91.7 | 39.8 |
| **75** | InChI=1S/C15H16N4O2/c16-19-14(20)12-6-8-13(9-7-12)18-15(21)17-10-11-4-2-1-3-5-11/h1-9H,10,16H2,(H,19,20)(H2,17,18,21) | >100 | >100 | 34.2 | 39.6 | 9.2 |
| **76** | InChI=1S/C20H18N4O3/c21-24-19(25)14-6-8-15(9-7-14)22-20(26)23-16-10-12-18(13-11-16)27-17-4-2-1-3-5-17/h1-13H,21H2,(H,24,25)(H2,22,23,26) | >100 | >100 | 23.1 | 7.3 | 3.8 |
| **77** | InChI=1S/C15H16N4O3/c1-22-13-4-2-3-12(9-13)18-15(21)17-11-7-5-10(6-8-11)14(20)19-16/h2-9H,16H2,1H3,(H,19,20)(H2,17,18,21) | >100 | 96.6 | 29.7 | 79.3 | 6.5 |
| **78** | InChI=1S/C16H16N4O4/c17-20-15(21)10-1-3-11(4-2-10)18-16(22)19-12-5-6-13-14(9-12)24-8-7-23-13/h1-6,9H,7-8,17H2,(H,20,21)(H2,18,19,22) | >100 | >100 | 2.2 | 13.9 | 5.0 |
| **79** | InChI=1S/C14H13FN4O2/c15-10-4-6-11(7-5-10)17-14(21)18-12-3-1-2-9(8-12)13(20)19-16/h1-8H,16H2,(H,19,20)(H2,17,18,21) | 21.6 | 90.8 | 93.5 | 0.94 | 42.5 |
| **80** | InChI=1S/C14H13ClN4O2/c15-10-4-6-11(7-5-10)17-14(21)18-12-3-1-2-9(8-12)13(20)19-16/h1-8H,16H2,(H,19,20)(H2,17,18,21) | >100 | 62.8 | >100 | 18.5 | 23.6 |
| **81** | InChI=1S/C15H13F3N4O3/c16-15(17,18)25-12-6-4-10(5-7-12)20-14(24)21-11-3-1-2-9(8-11)13(23)22-19/h1-8H,19H2,(H,22,23)(H2,20,21,24) | >100 | 64.8 | 61.8 | 23.6 | 67.0 |
| **82** | InChI=1S/C13H14N4O3/c14-17-12(18)9-3-1-4-10(7-9)16-13(19)15-8-11-5-2-6-20-11/h1-7H,8,14H2,(H,17,18)(H2,15,16,19) | >100 | >100 | 0.96 | 34.4 | 28.3 |
| **83** | InChI=1S/C15H16N4O2/c16-19-14(20)12-7-4-8-13(9-12)18-15(21)17-10-11-5-2-1-3-6-11/h1-9H,10,16H2,(H,19,20)(H2,17,18,21) | 75.6 | 89.0 | 48.4 | 9.3 | 7.7 |
| **84** | InChI=1S/C20H18N4O3/c21-24-19(25)14-5-4-6-16(13-14)23-20(26)22-15-9-11-18(12-10-15)27-17-7-2-1-3-8-17/h1-13H,21H2,(H,24,25)(H2,22,23,26) | >100 | 87.1 | 24.6 | 20.9 | 8.5 |
| **85** | InChI=1S/C15H16N4O3/c1-22-13-7-3-6-12(9-13)18-15(21)17-11-5-2-4-10(8-11)14(20)19-16/h2-9H,16H2,1H3,(H,19,20)(H2,17,18,21) | >100 | >100 | 2.0 | 80.6 | 6.9 |
| **86** | InChI=1S/C16H16N4O4/c17-20-15(21)10-2-1-3-11(8-10)18-16(22)19-12-4-5-13-14(9-12)24-7-6-23-13/h1-5,8-9H,6-7,17H2,(H,20,21)(H2,18,19,22) | >100 | 17.2 | 0.80 | 9.6 | 12.0 |

^a^ *Carbonic anhydrase inhibition.* An Applied Photophysics stopped-flow instrument has been used for assaying the CA-catalysed CO_2_ hydration activity.^[30]^ Phenol red (at a concentration of 0.2 mM) has been used as an indicator, working at the absorbance maximum of 557 nm, with 20 mM Hepes (pH 7.5) as a buffer and 20 mM Na_2_SO_4_ (for maintaining constant the ionic strength), following the initial rates of the CA-catalysed CO_2_ hydration reaction for a period of 10-100 s. The CO_2_ concentrations ranged from 1.7 to 17 mM for the determination of the kinetic parameters and inhibition constants. For each inhibitor, at least six traces of the initial 5-10% of the reaction have been used for determining the initial velocity. The uncatalysed rates were determined in the same manner and subtracted from the total observed rates. Stock solutions of inhibitor (0.1 mM) were prepared in distilled-deionized water and dilutions up to 0.01 nM were done thereafter with the assay buffer. Inhibitor and enzyme solutions were preincubated together for 30 min at room temperature before assay to allow for the formation of the E-I complex. The inhibition constants were obtained by nonlinear least-squares methods using PRISM 3 and the Cheng-Prusoff equation, as reported earlier,^[24]^ and represent the mean from at least three different determinations. The enzyme concentrations were in the range 6-14 nM. All hCA isoforms were recombinant ones obtained in-house as reported earlier.^[26]^

24) S. Giovannuzzi, C. Capasso, A. Nocentini, C. T. Supuran, *Molecules*. **2022**, 27, 4076.

26) S. Giovannuzzi, A. K. Marapaka, N. S. Abutaleb, F. Carta, H. W. Liang, A. Nocentini, L. Pisano, M. N. Seleem, D. P. Flaherty, C. T. Supuran, *J. Enzyme Inhib. Med. Chem*. **2023**, 38, 2284119.

30) R. G. Khalifah, *J. Biol. Chem*. **1971**, 246, 2561-2573.
