# Supplementary material for: Exploration of Aromatic Hydrazides as Inhibitors of Human Carbonic Anhydrases
Source: Arch Pharm (Weinheim). 2025 Apr 1;358(4):e202400963. doi: 10.1002/ardp.202400963 (PMC11959329; doi:10.1002/ardp.202400963)

Supplementary information for

**Exploration of aromatic hydrazides as inhibitors of human carbonic anhydrases**

German Benito Menendez,^1^ Simone Giovannuzzi,^1,^* Alessandro Bonardi,^1,2,^* Alessio Nocentini,^1,2^ Paola Gratteri,^1,2^ Claudiu T. Supuran.^1^

1 NEUROFARBA Department, Pharmaceutical and Nutraceutical Section, University of Florence, 50019, Sesto Fiorentino, Florence, Italy

2 NEUROFARBA Department, Pharmaceutical and Nutraceutical Section, Laboratory of Molecular Modeling Cheminformatics & QSAR, University of Florence, Via U. Schiff 6, 50019, Sesto Fiorentino, Florence, Italy

**Table of contents**

^1^H-NMR, ^13^C-NMR and ^19^F-NMR spectra of compounds **49**-**86** S3


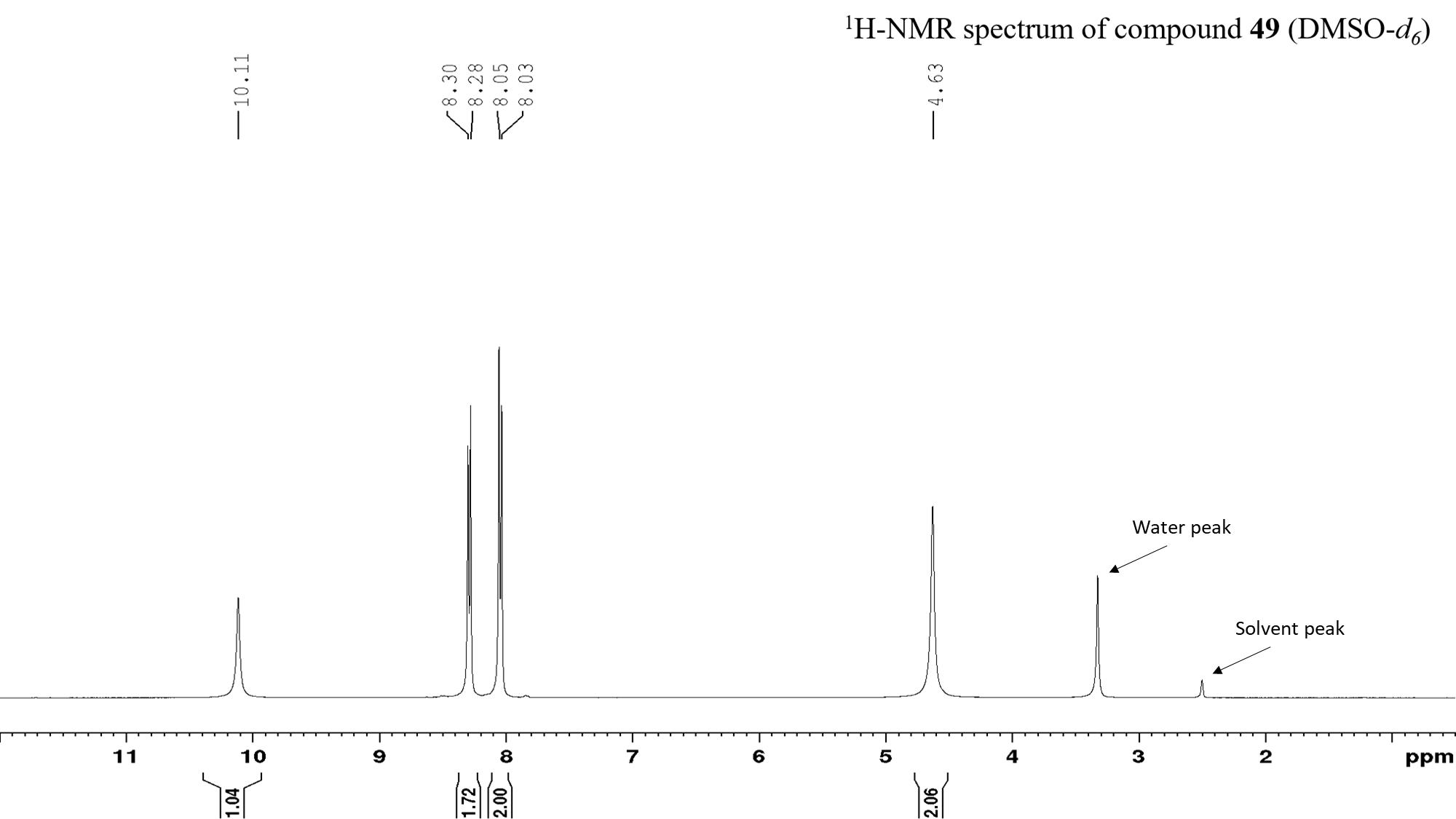


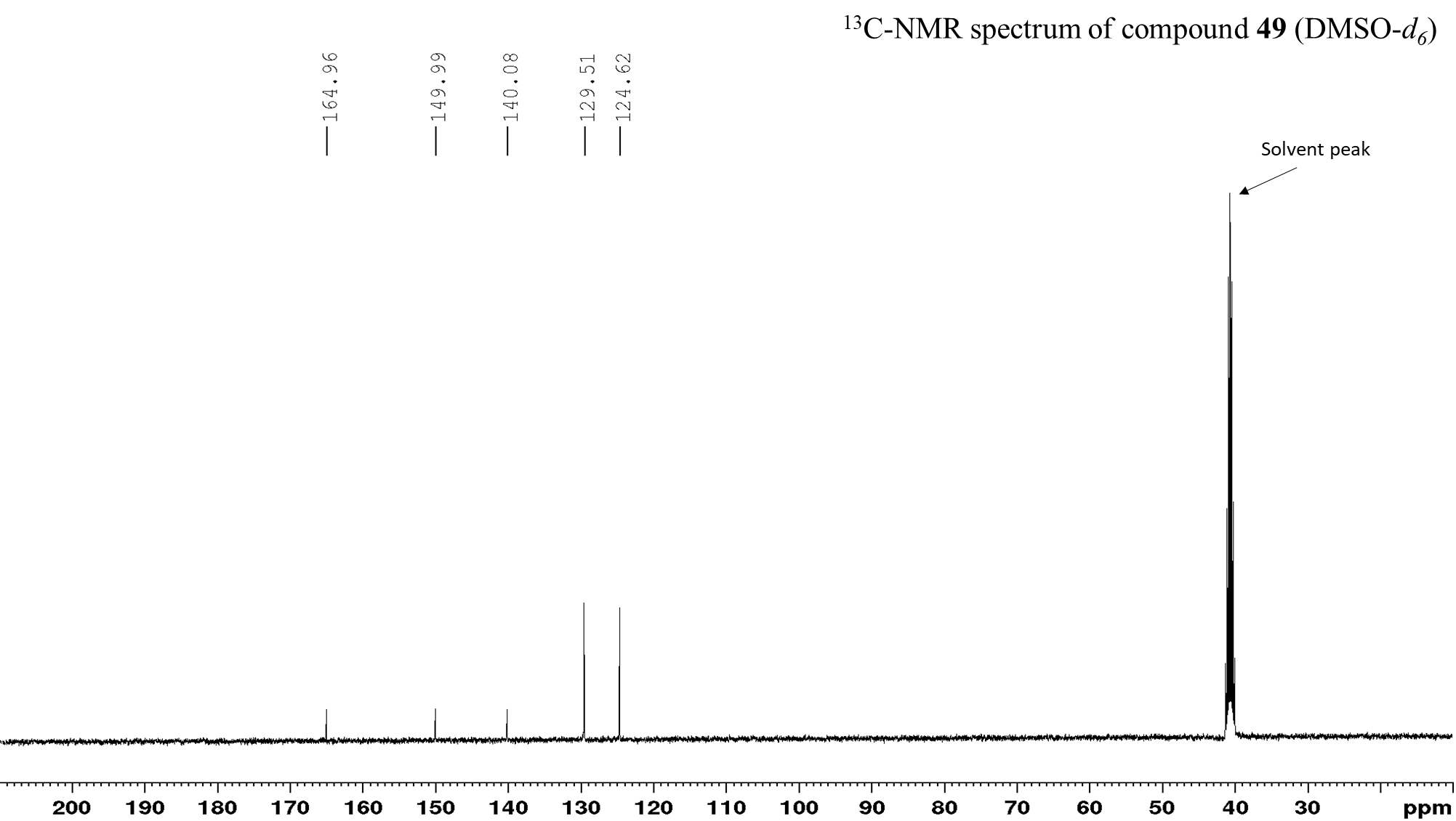


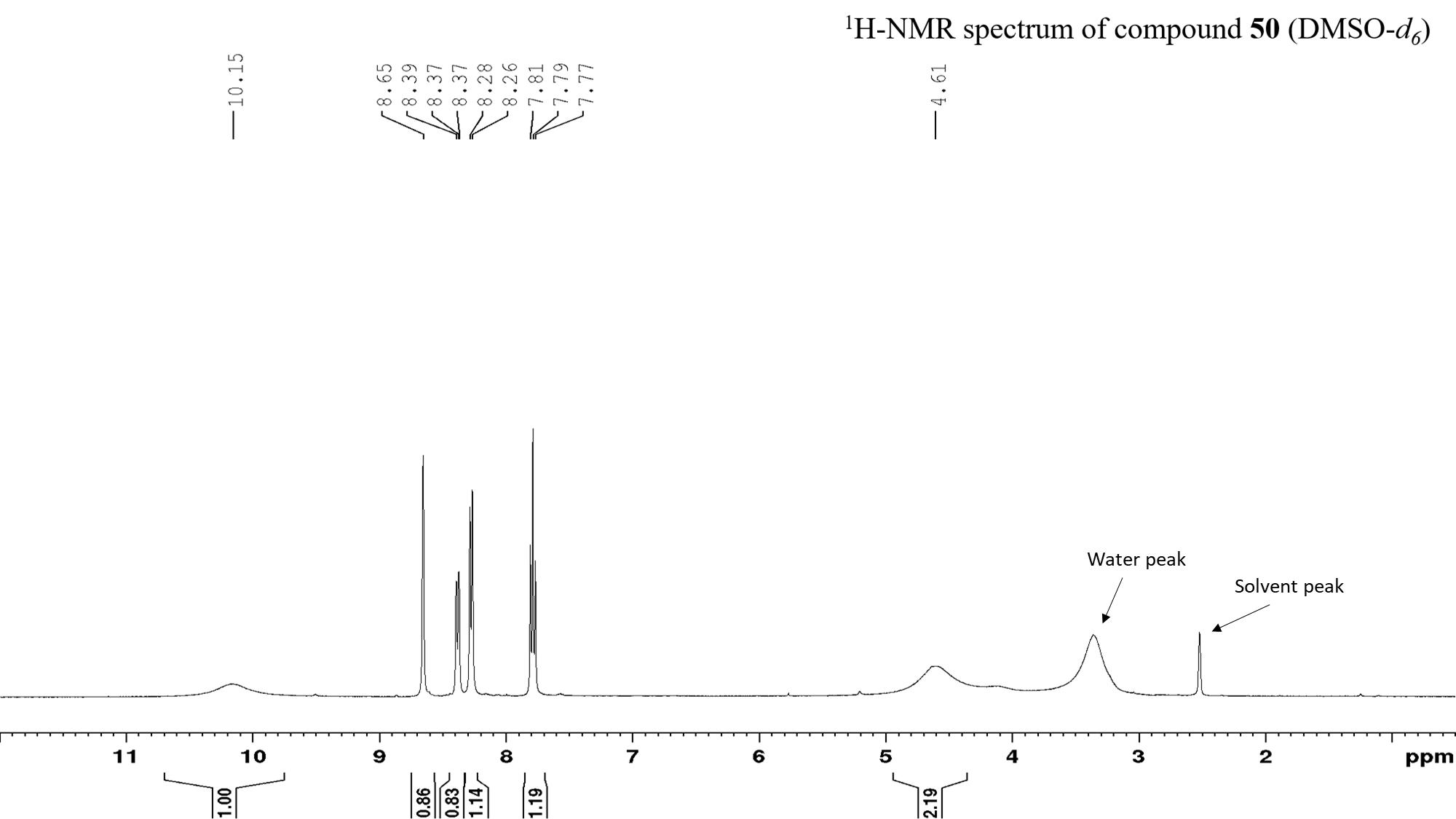


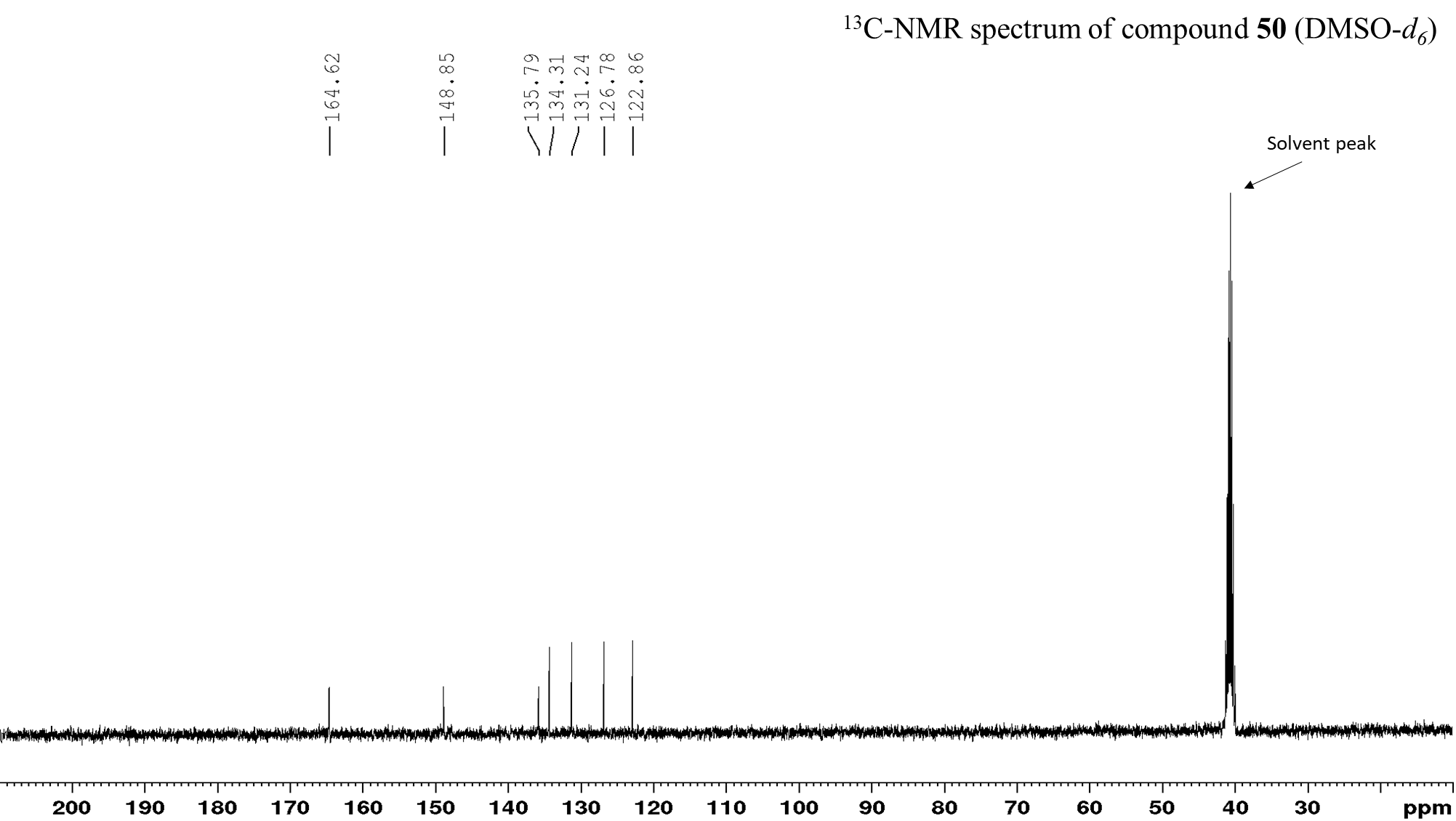


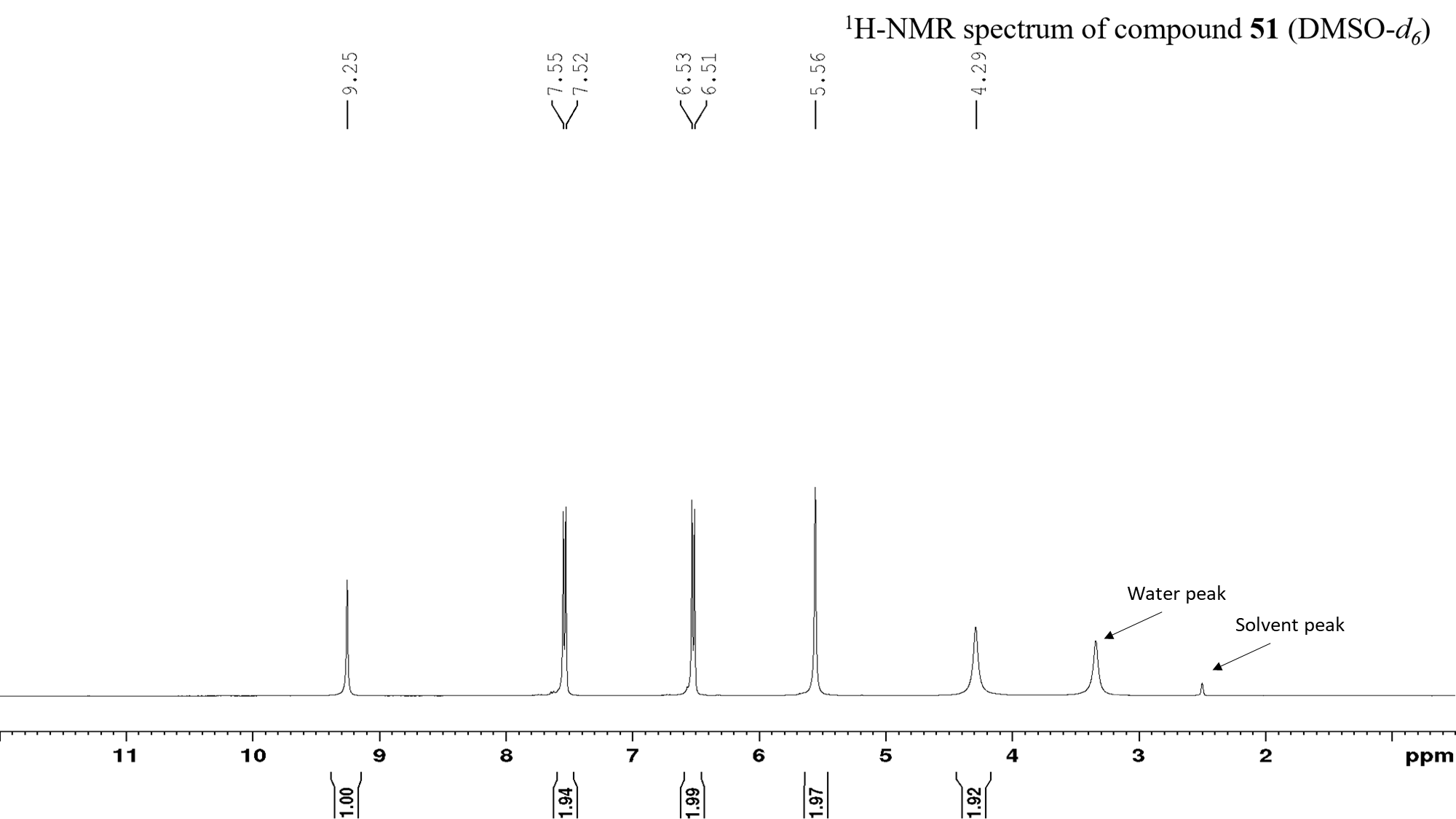


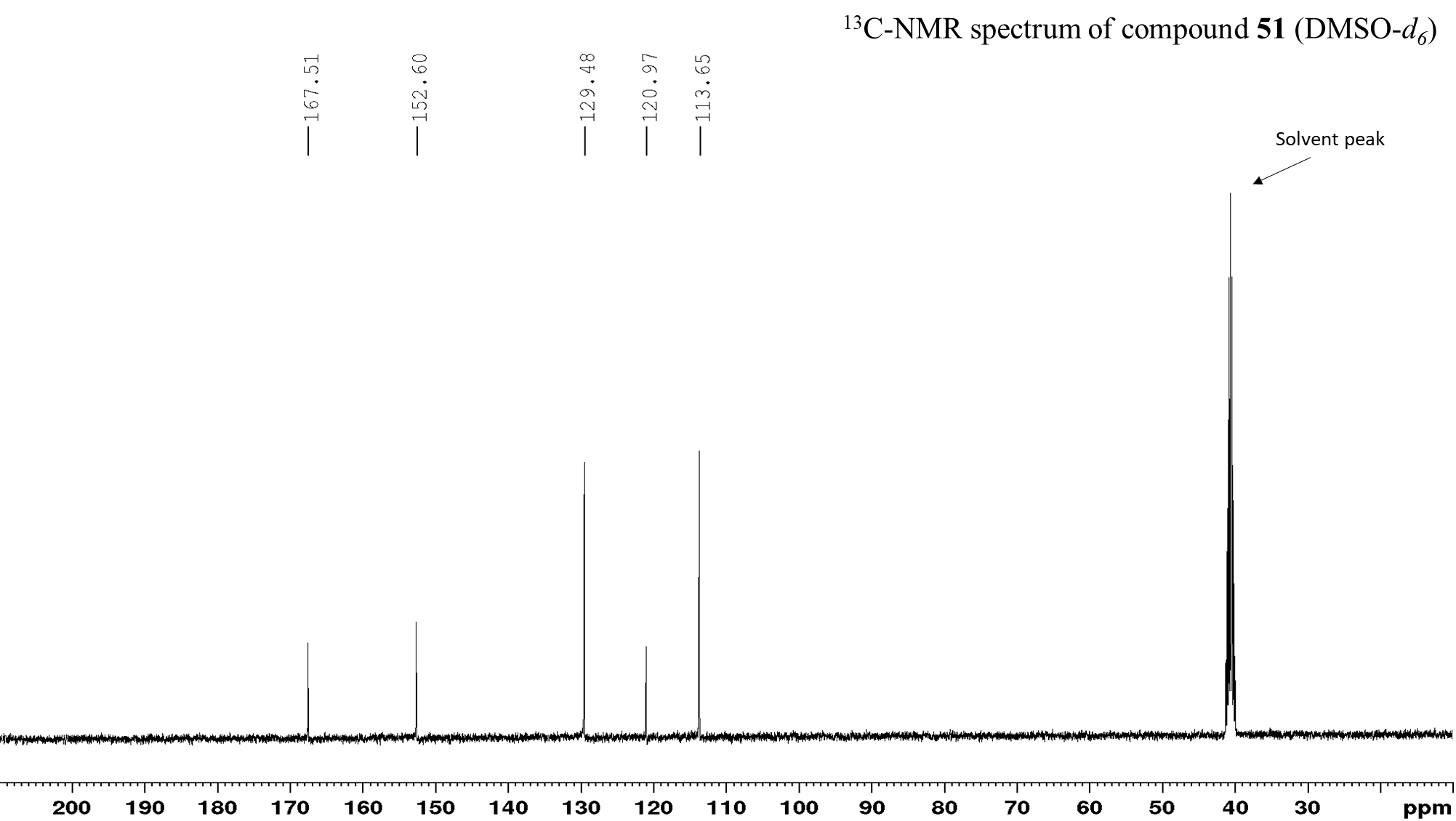


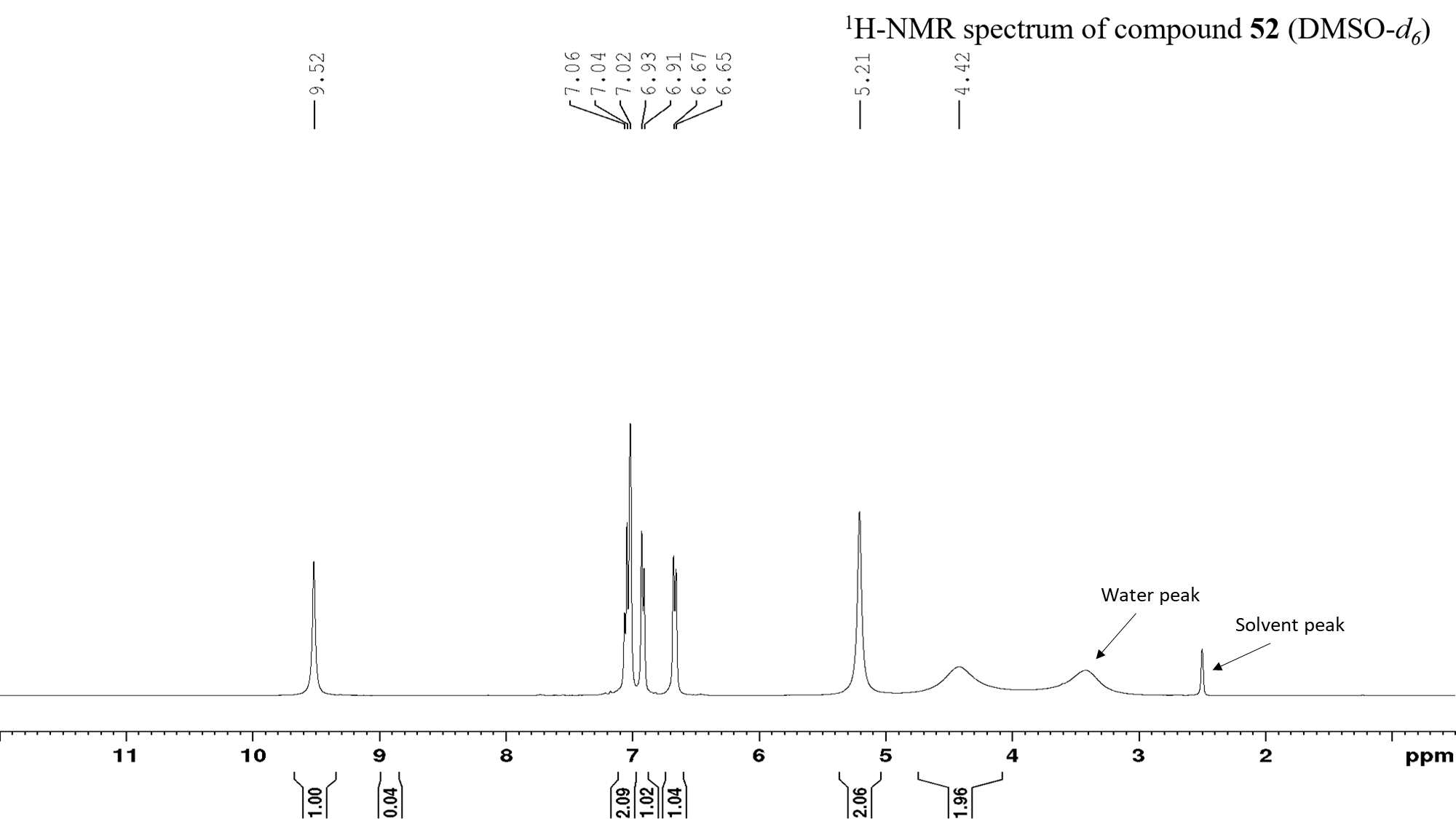


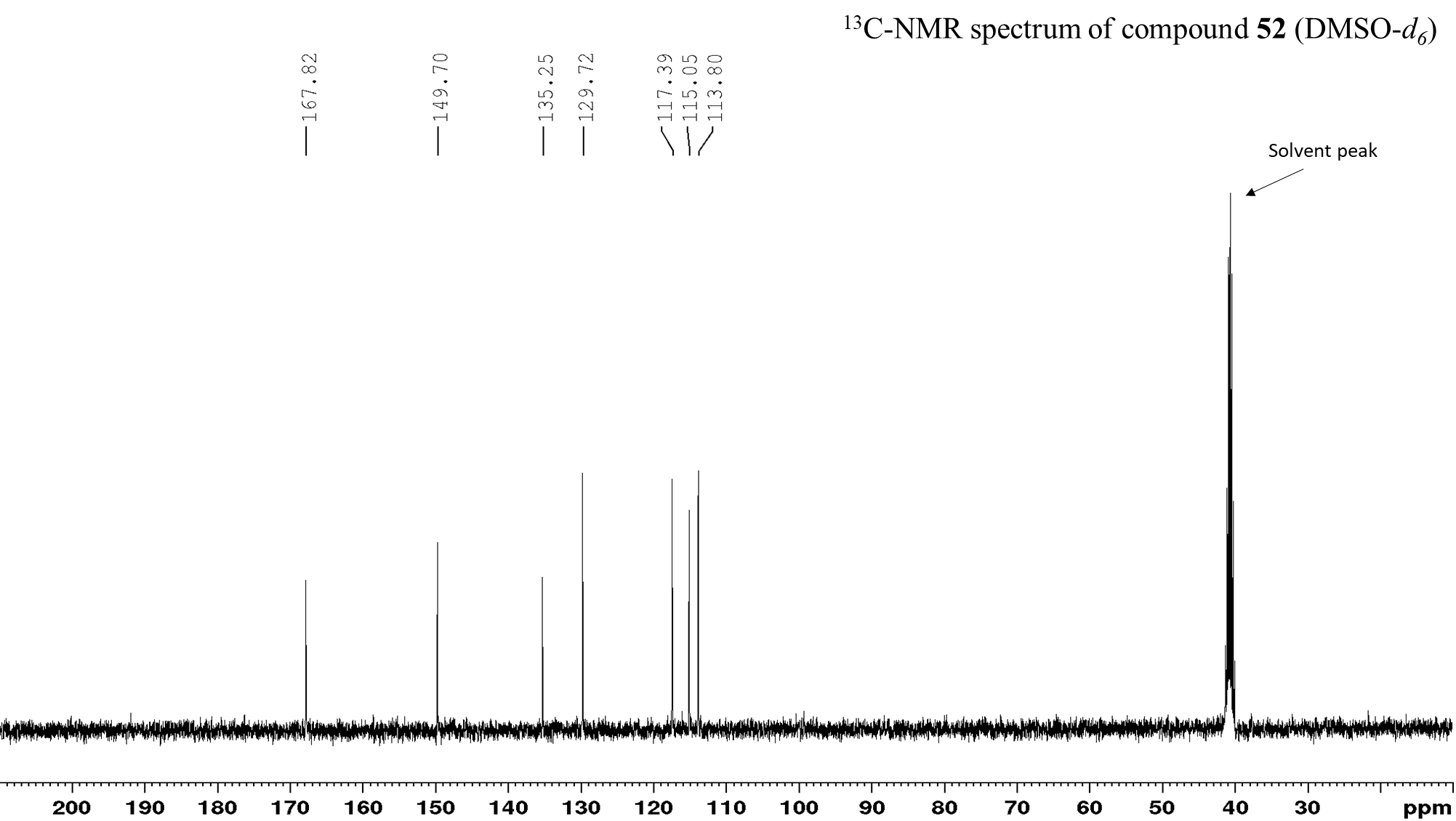


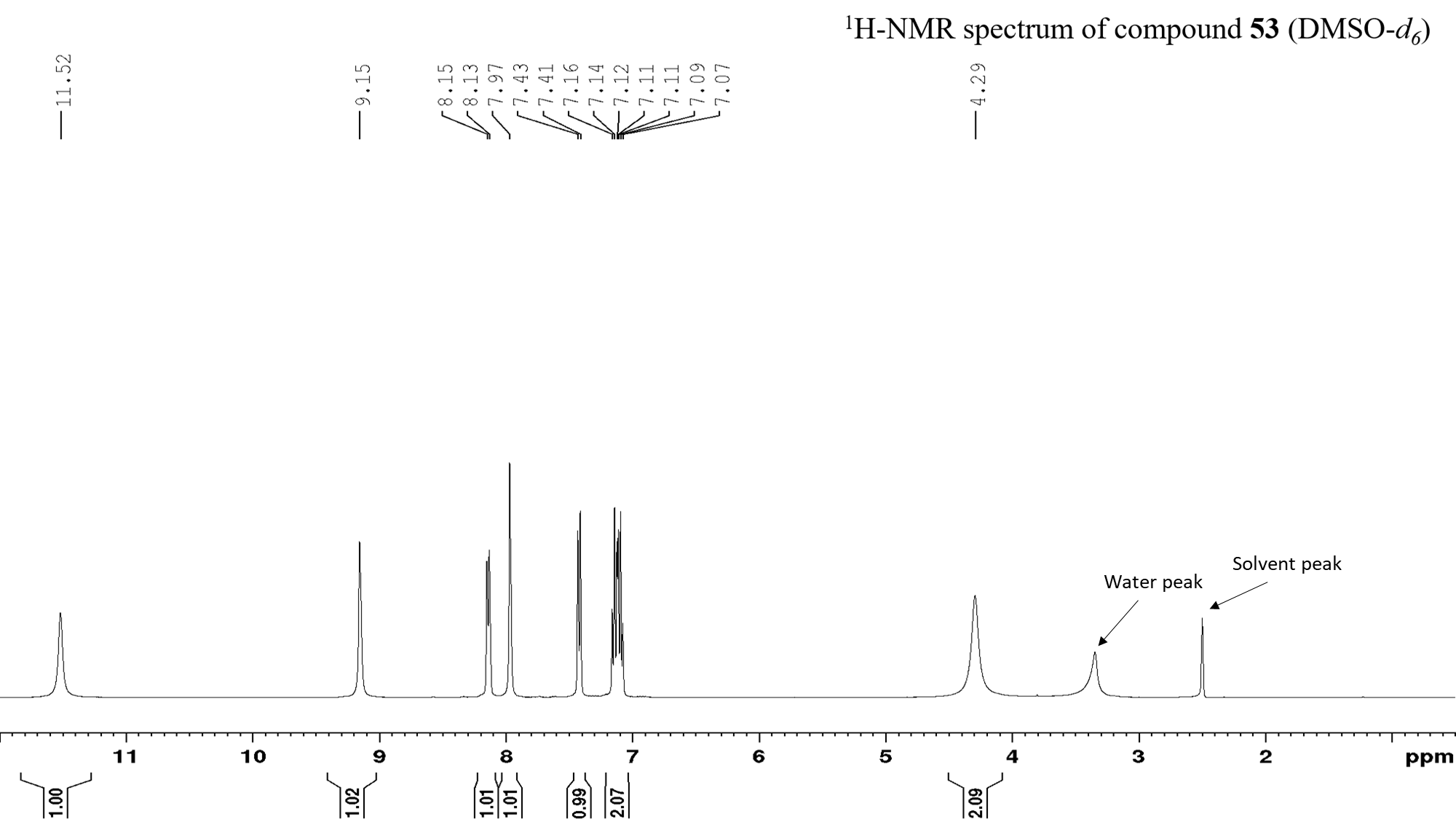


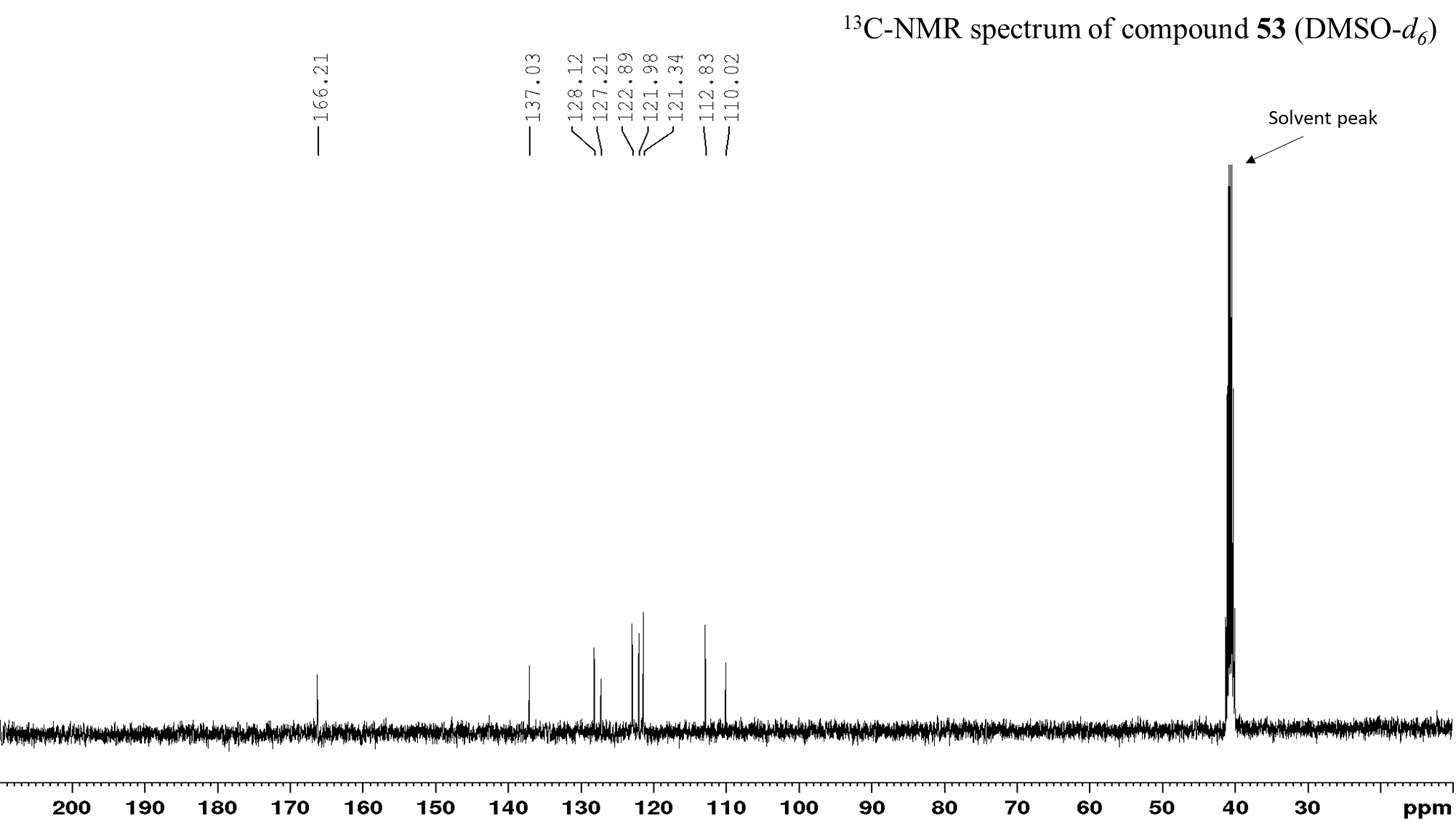


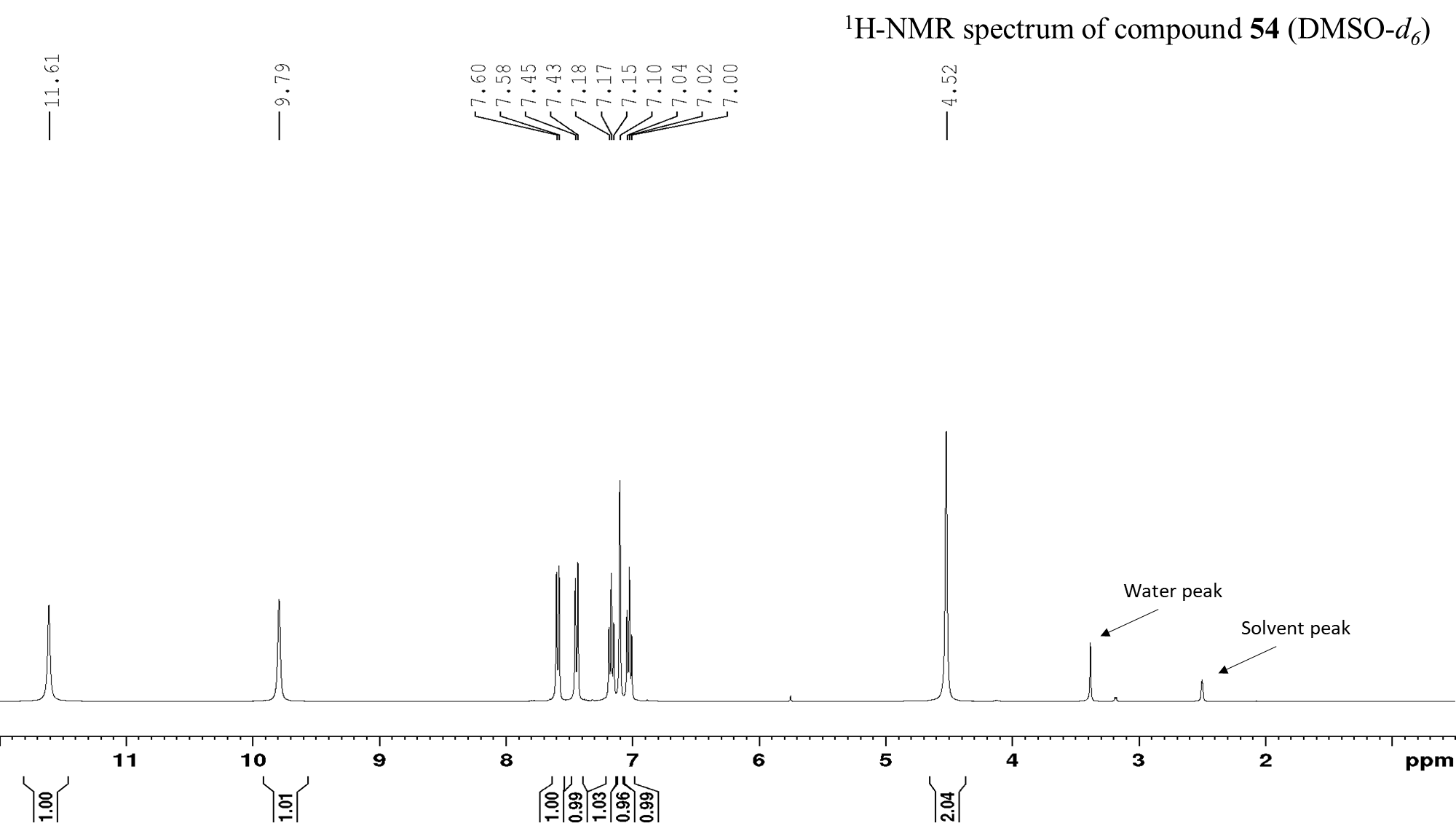


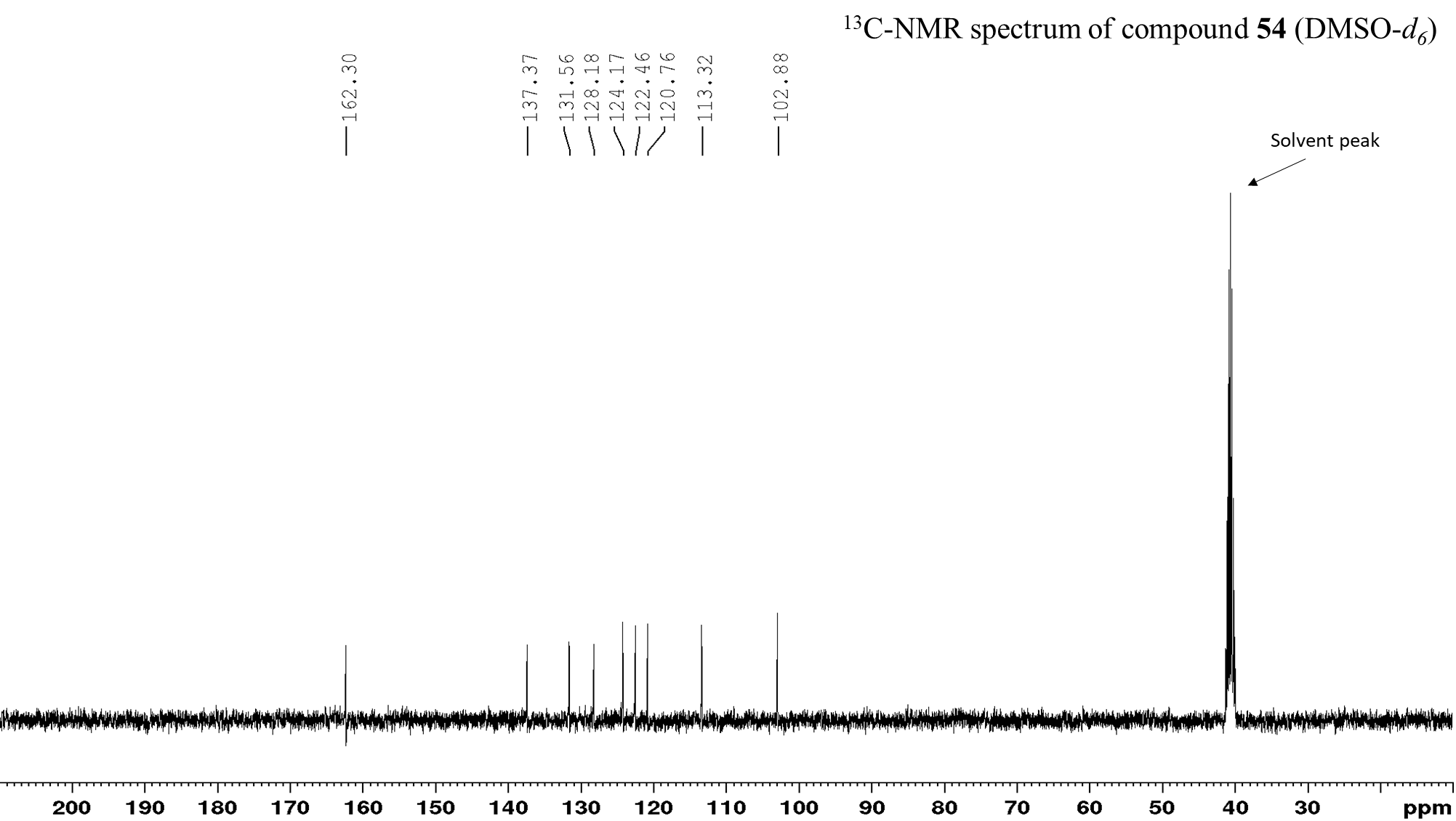


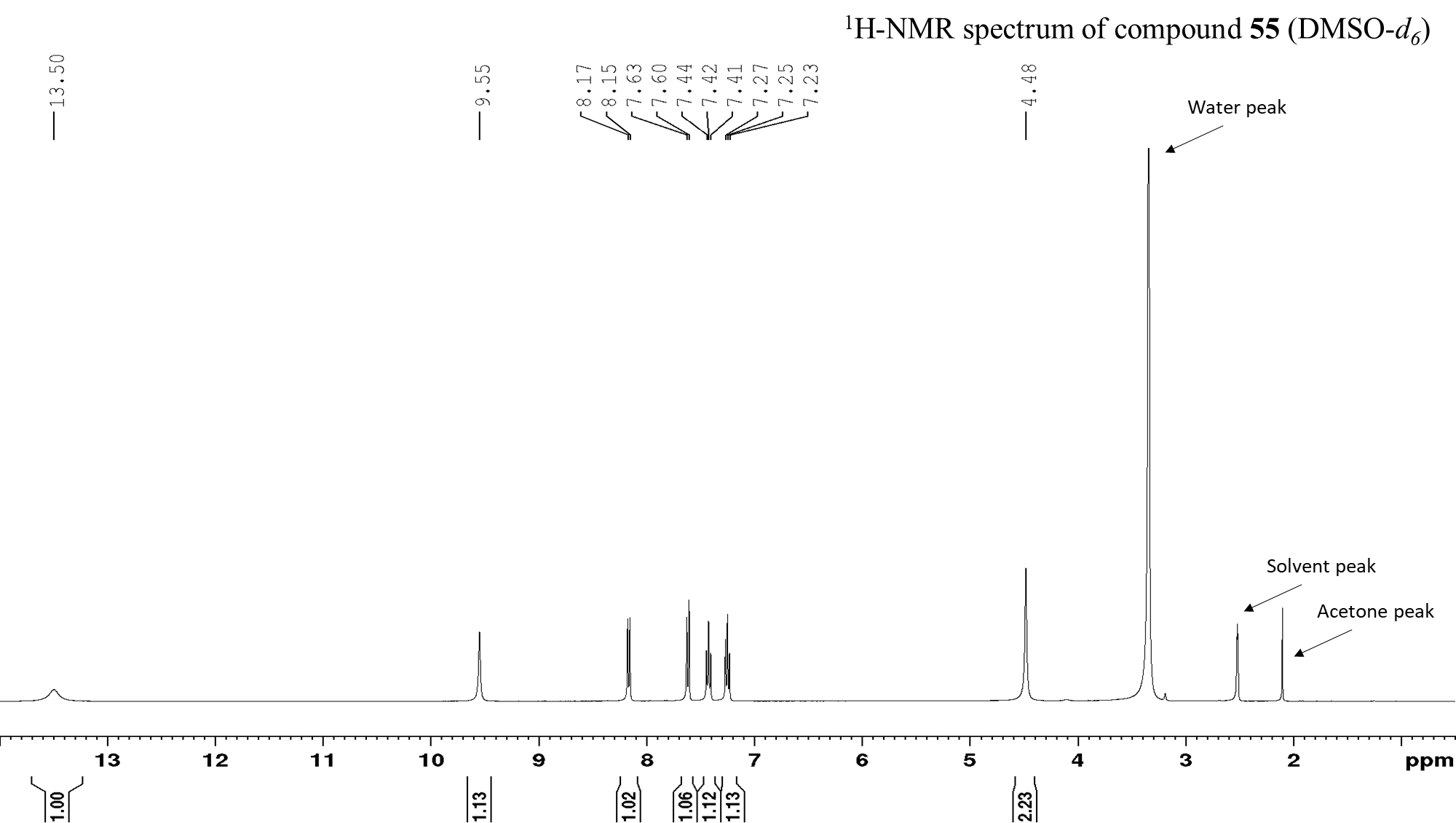


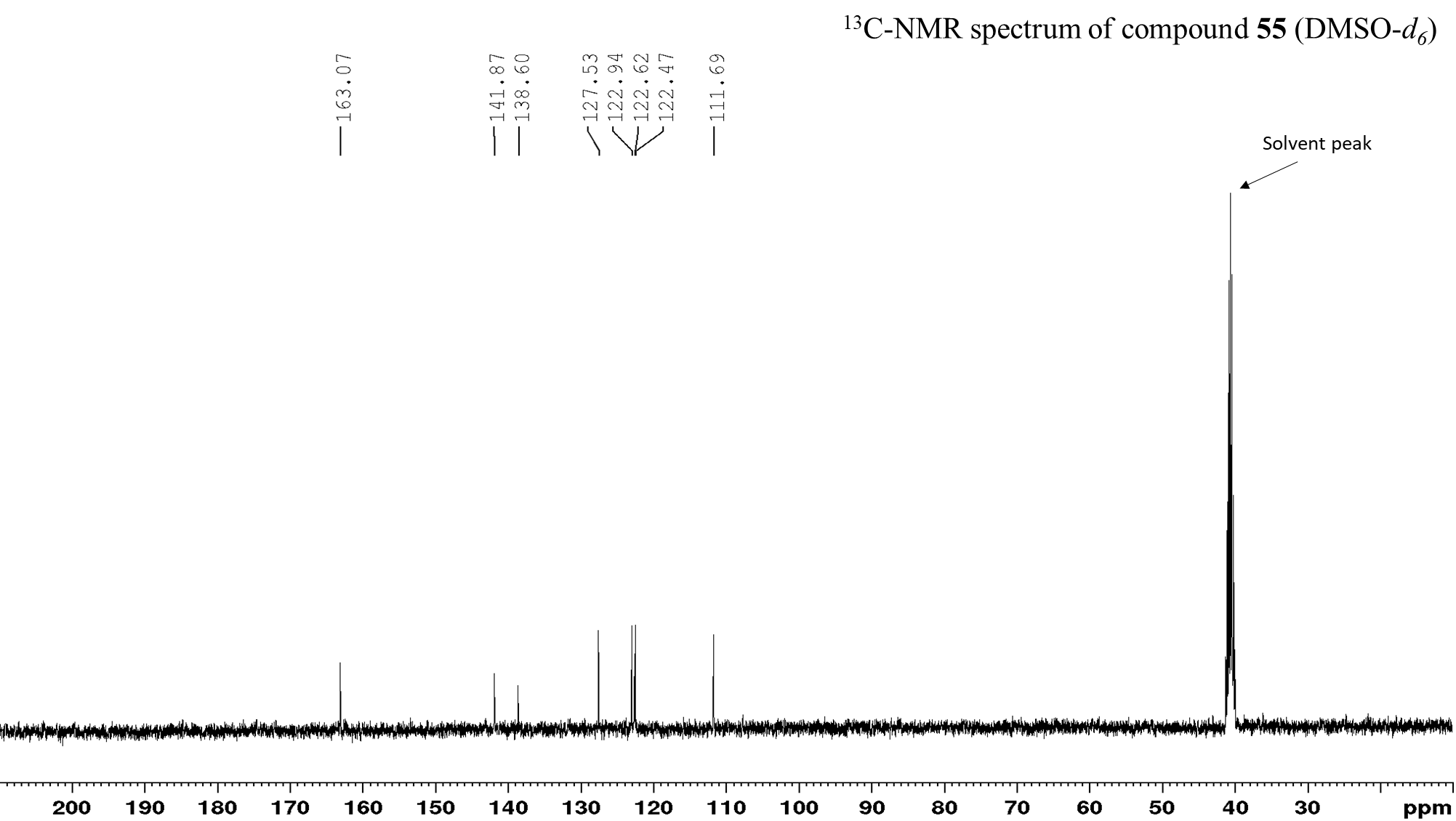


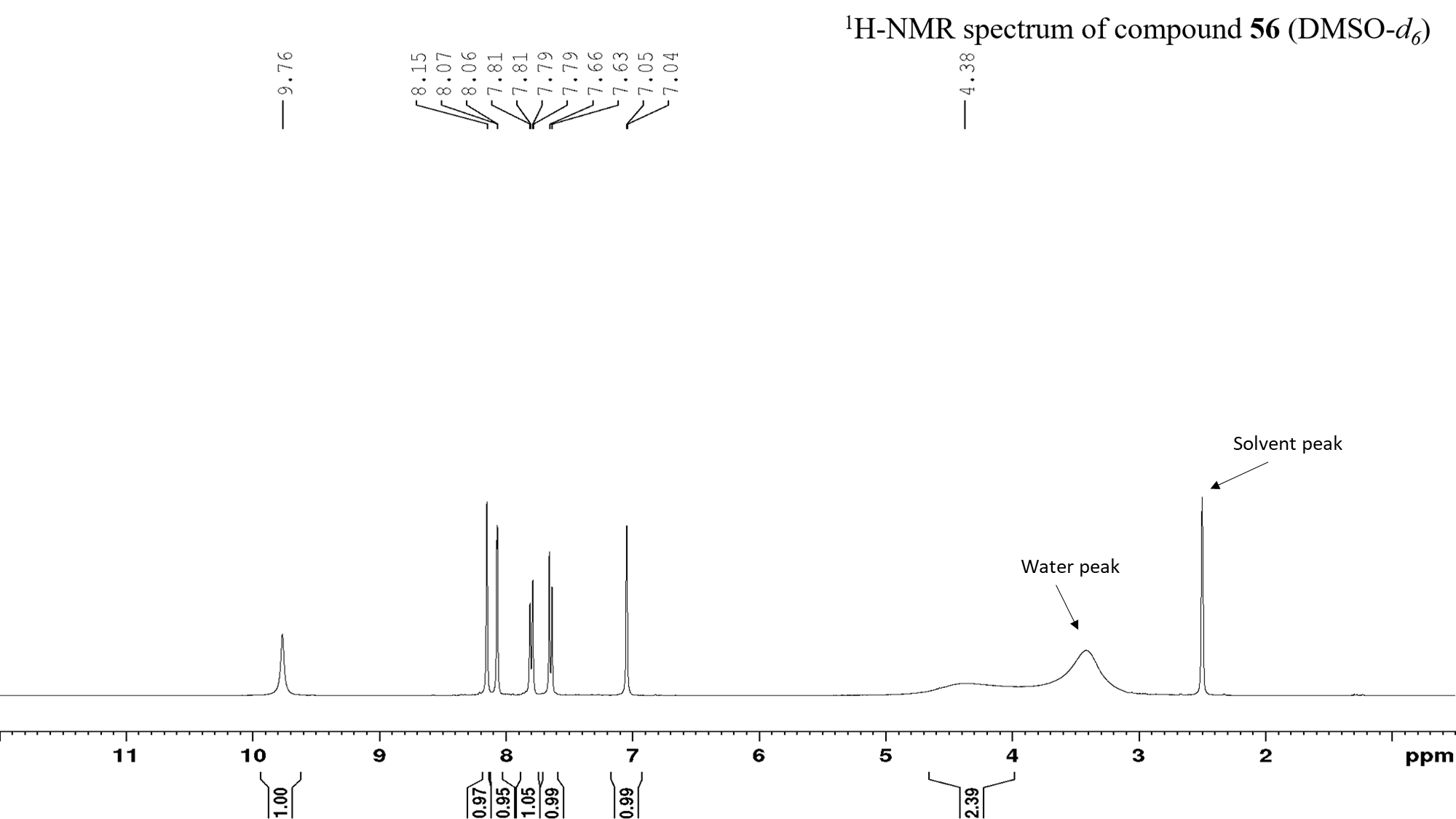


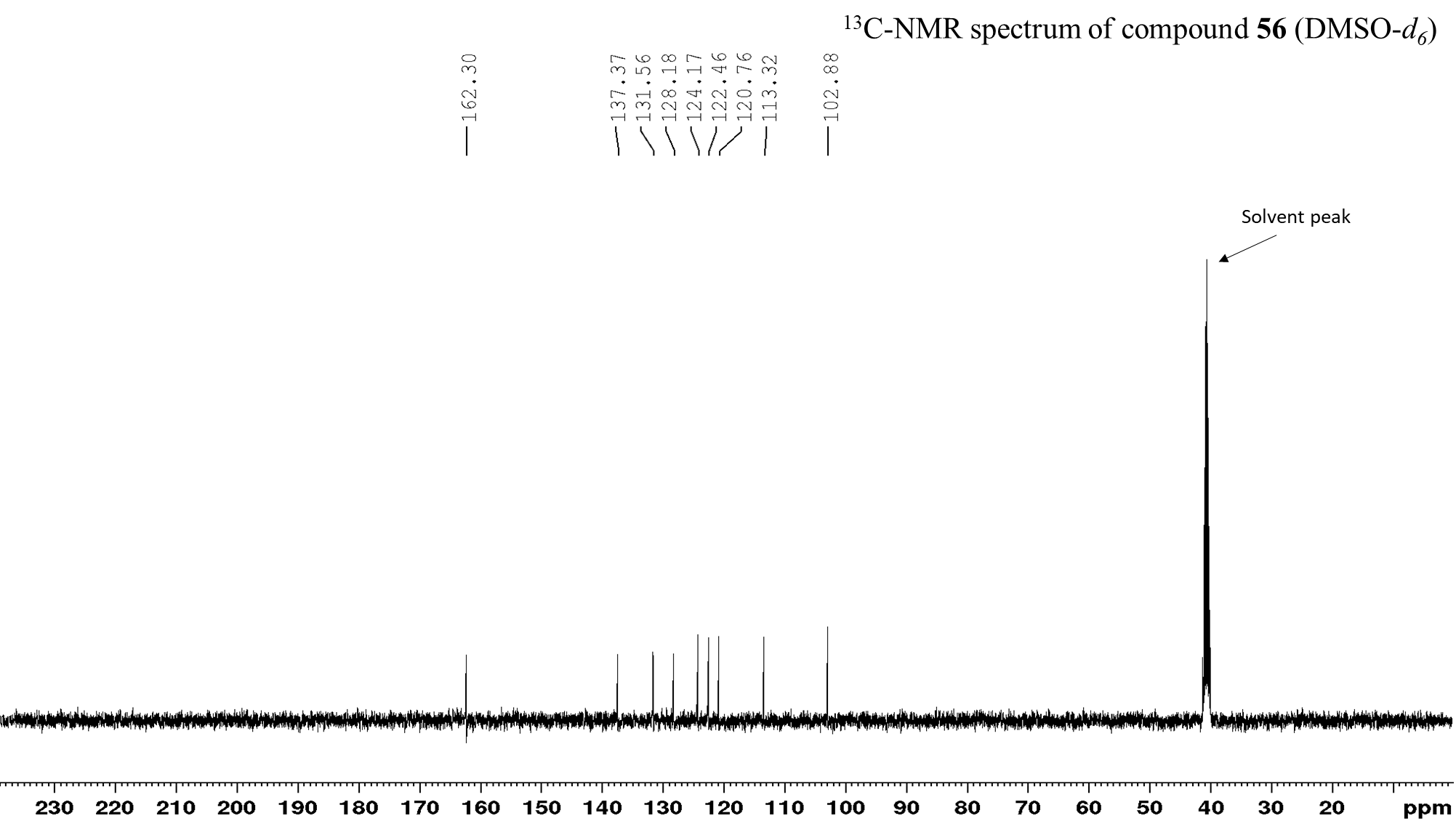


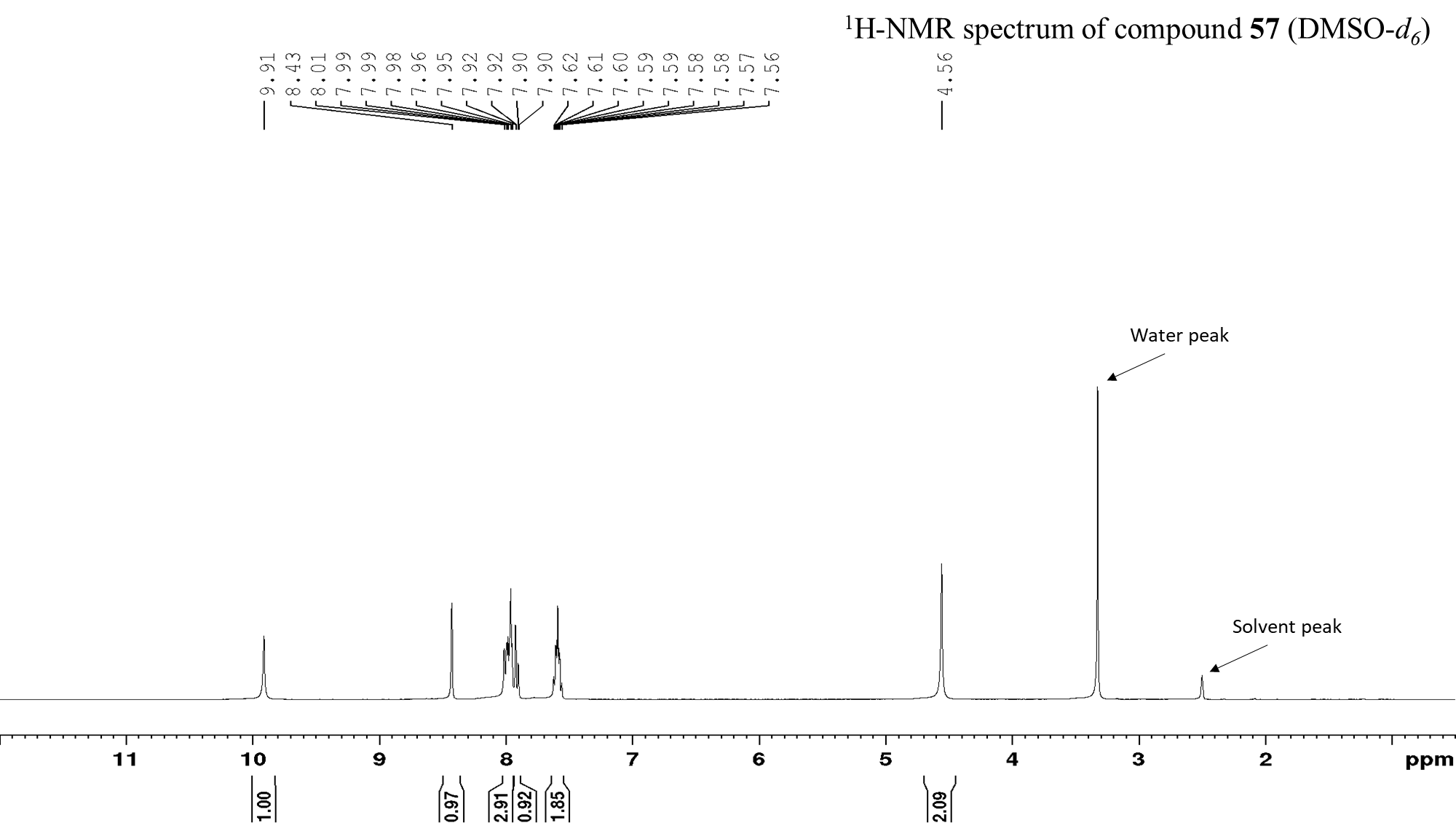


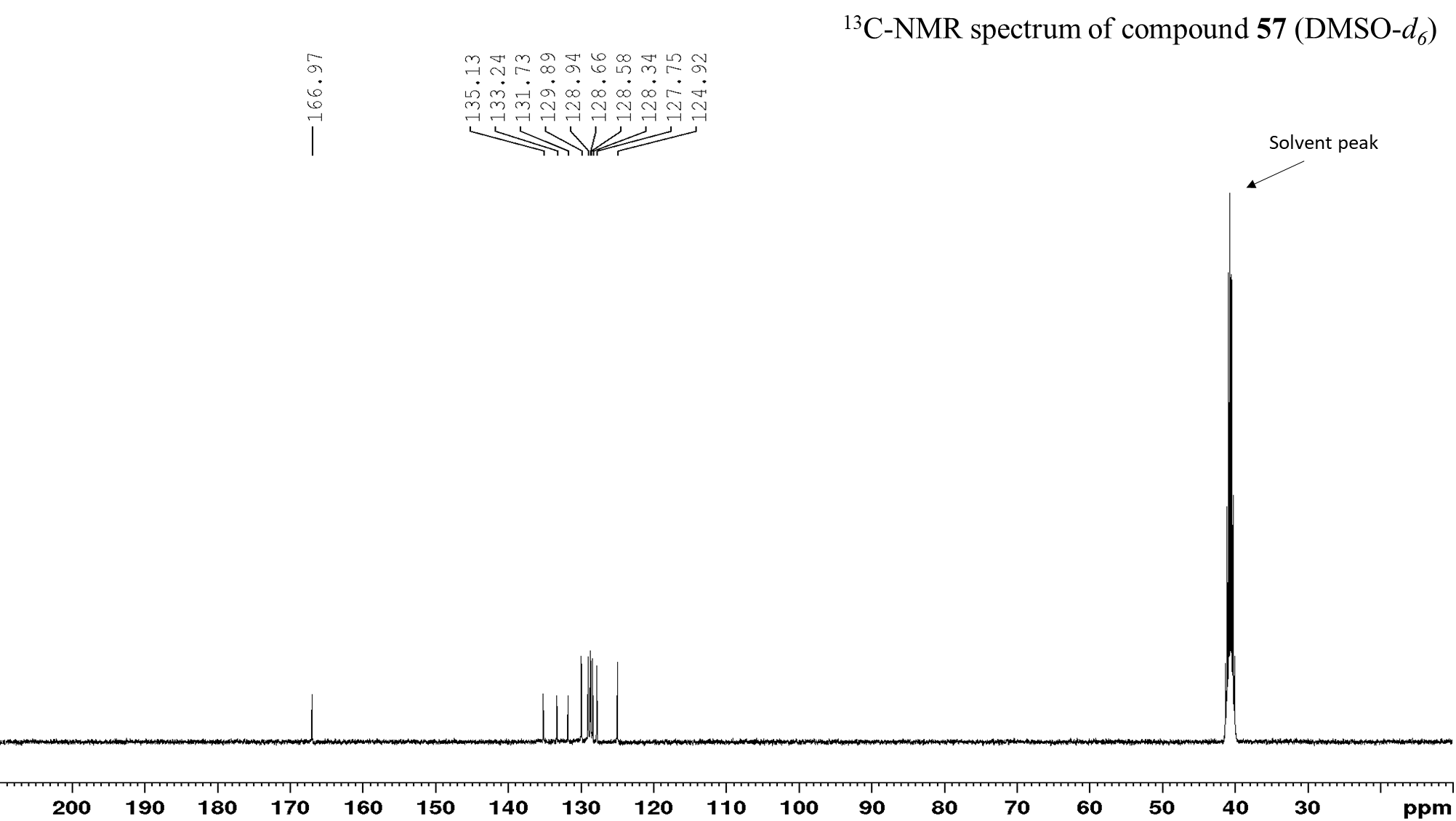


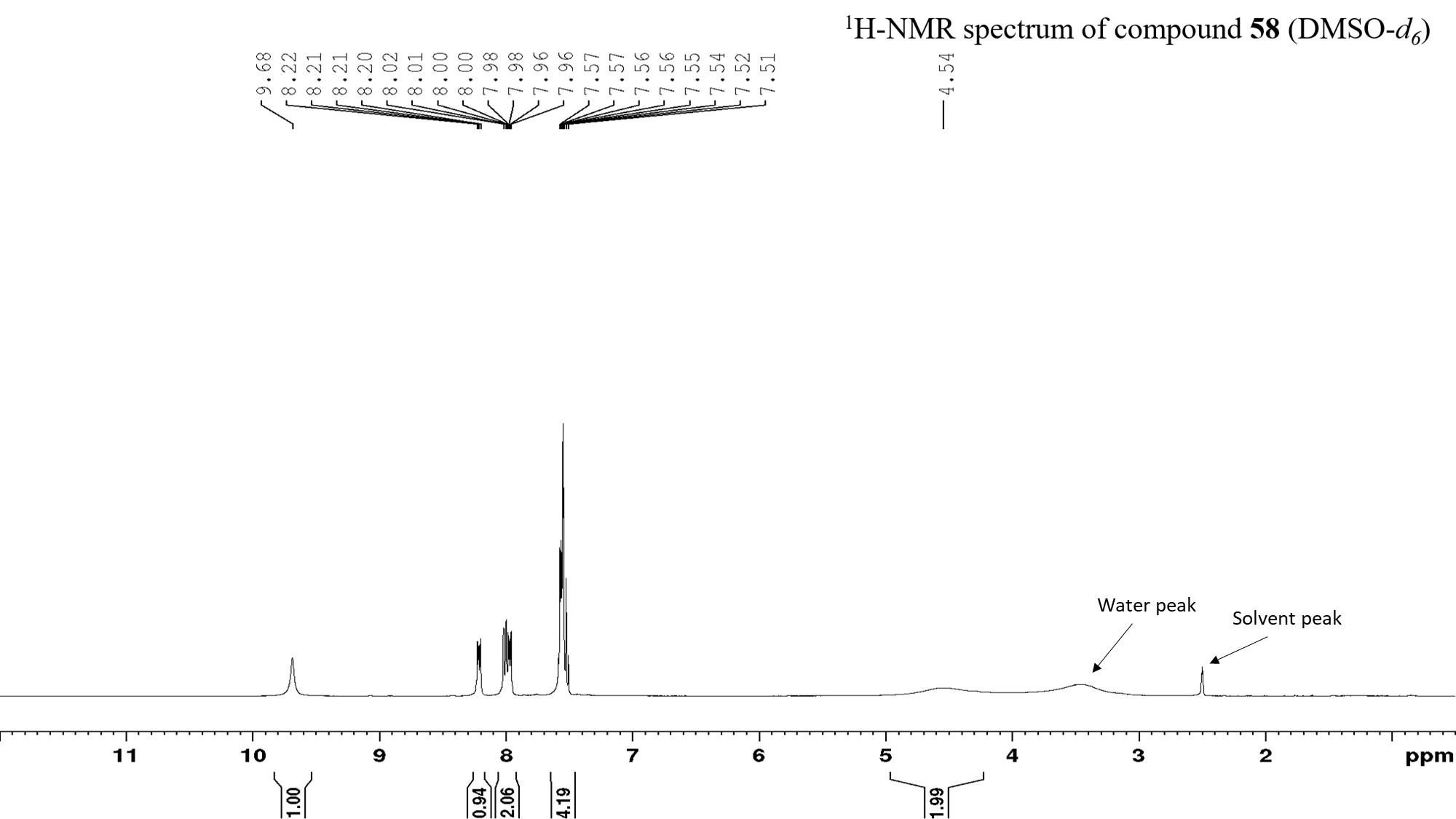


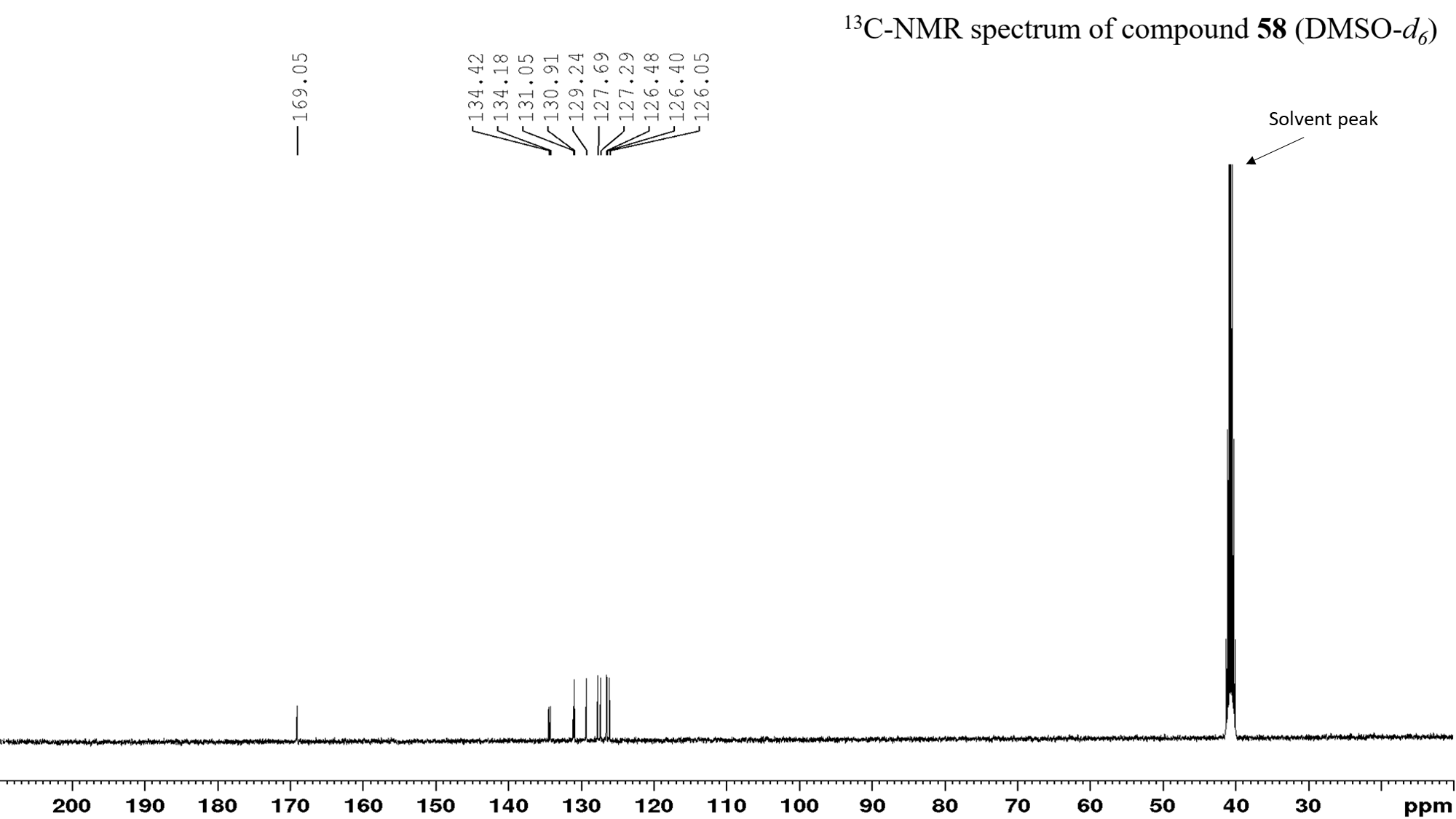


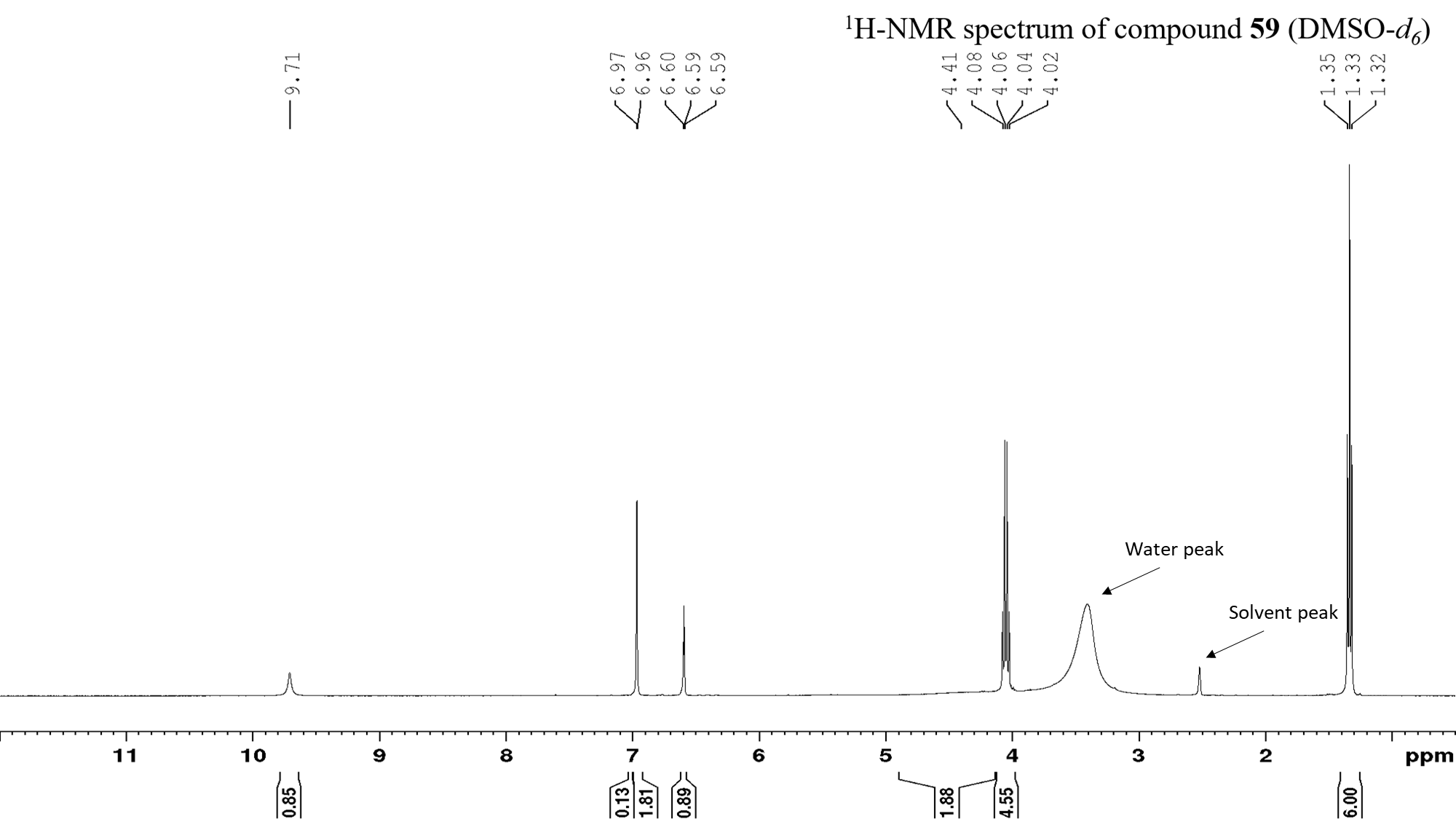


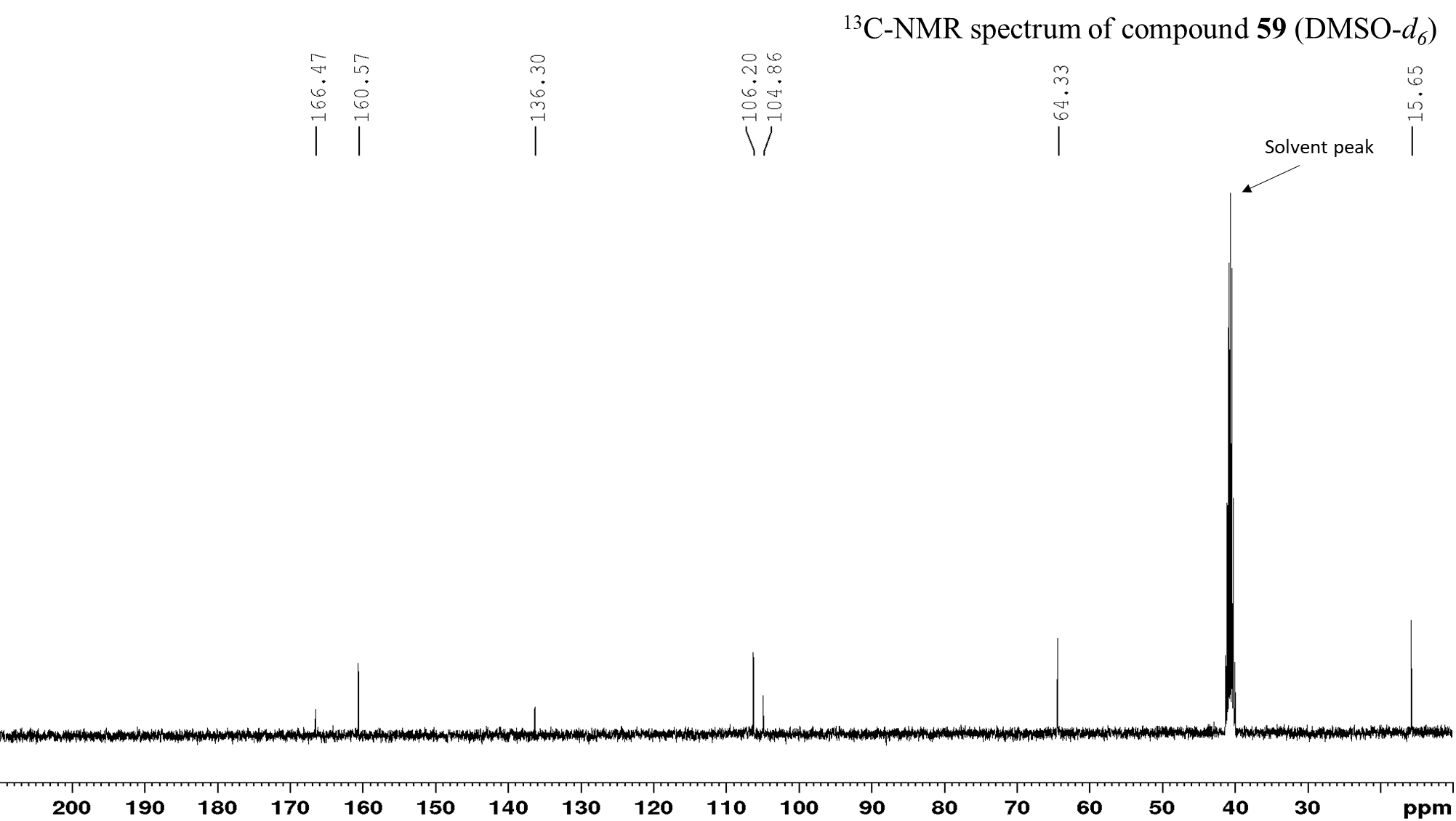


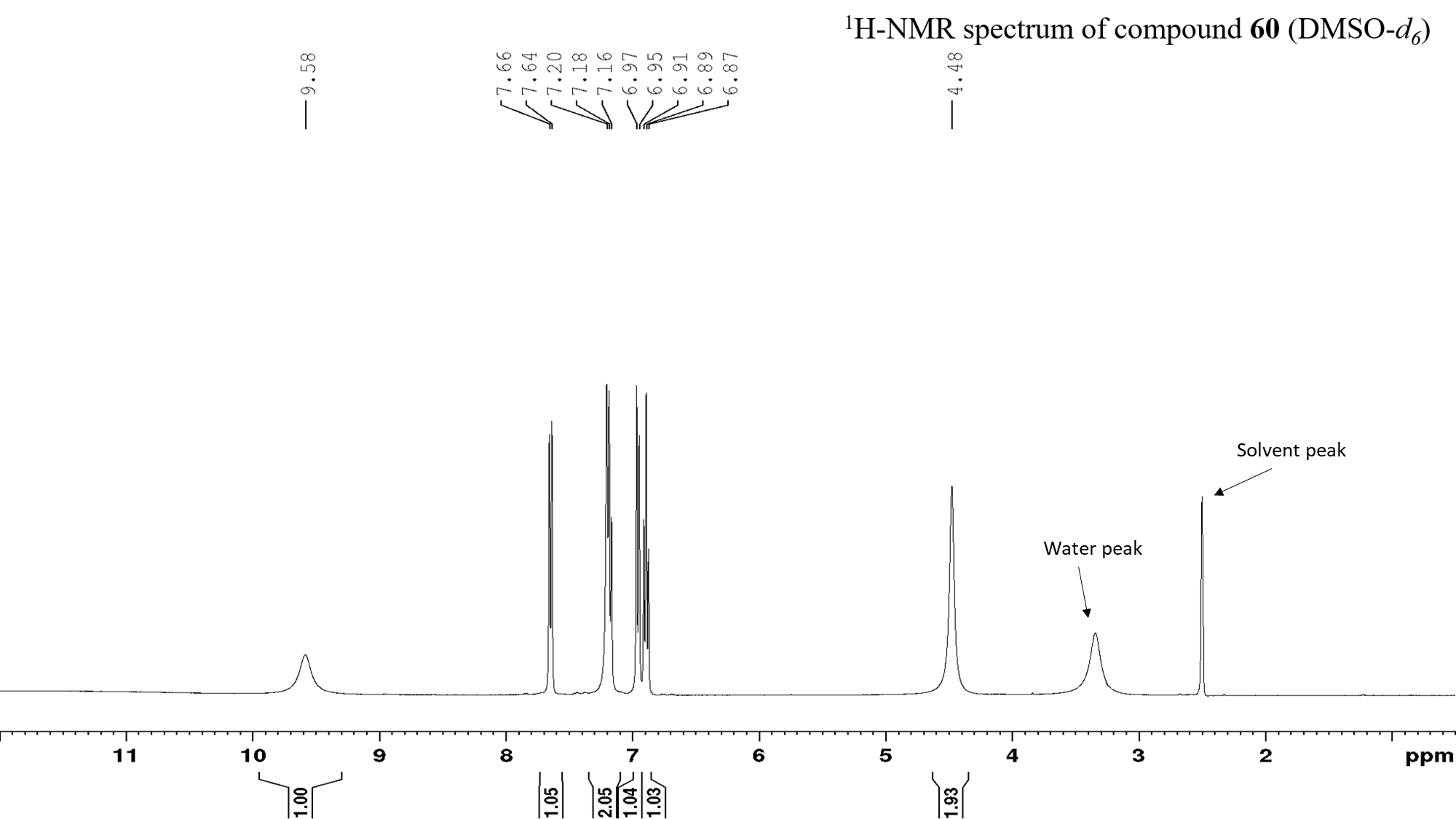


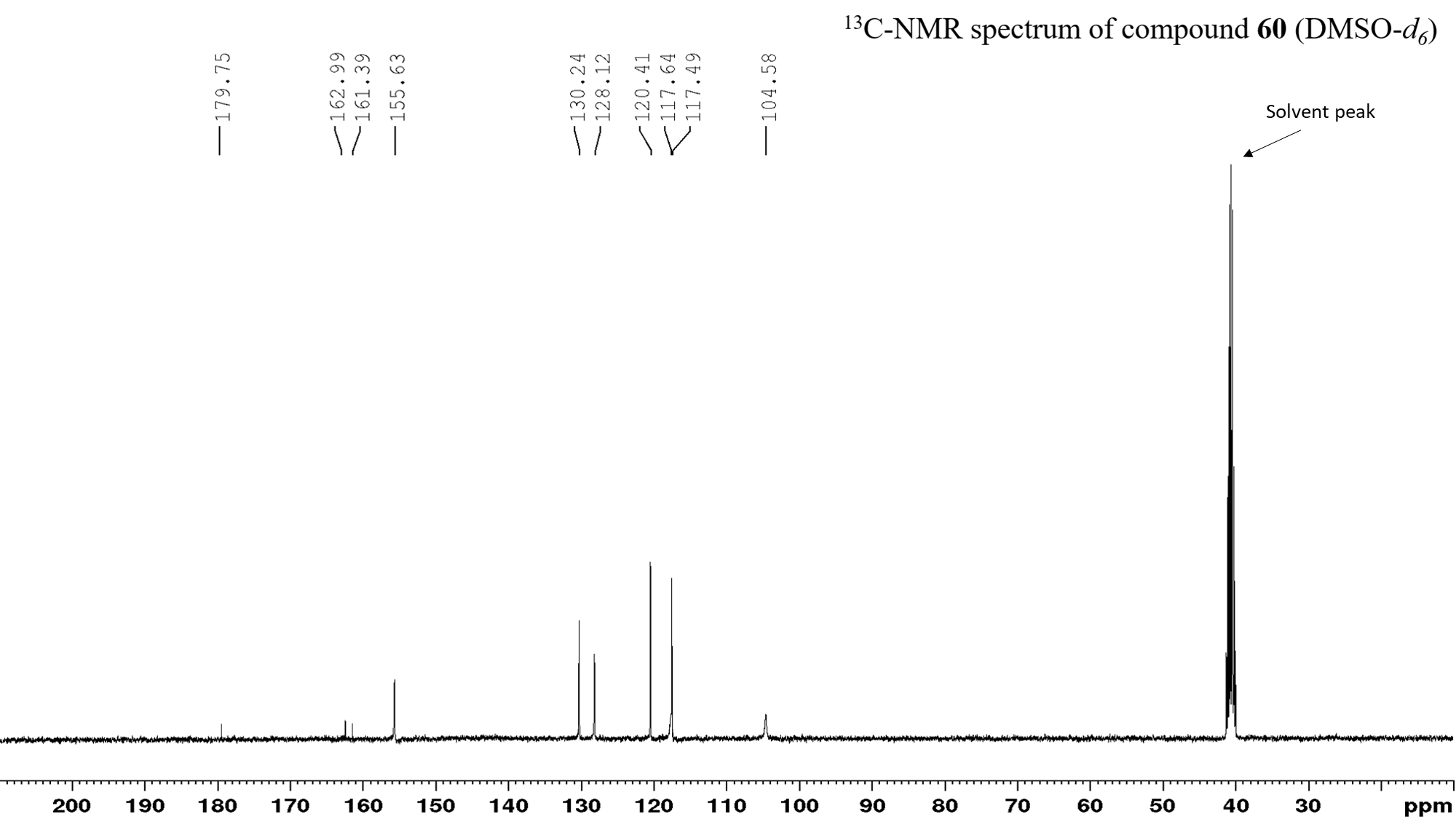


9,5


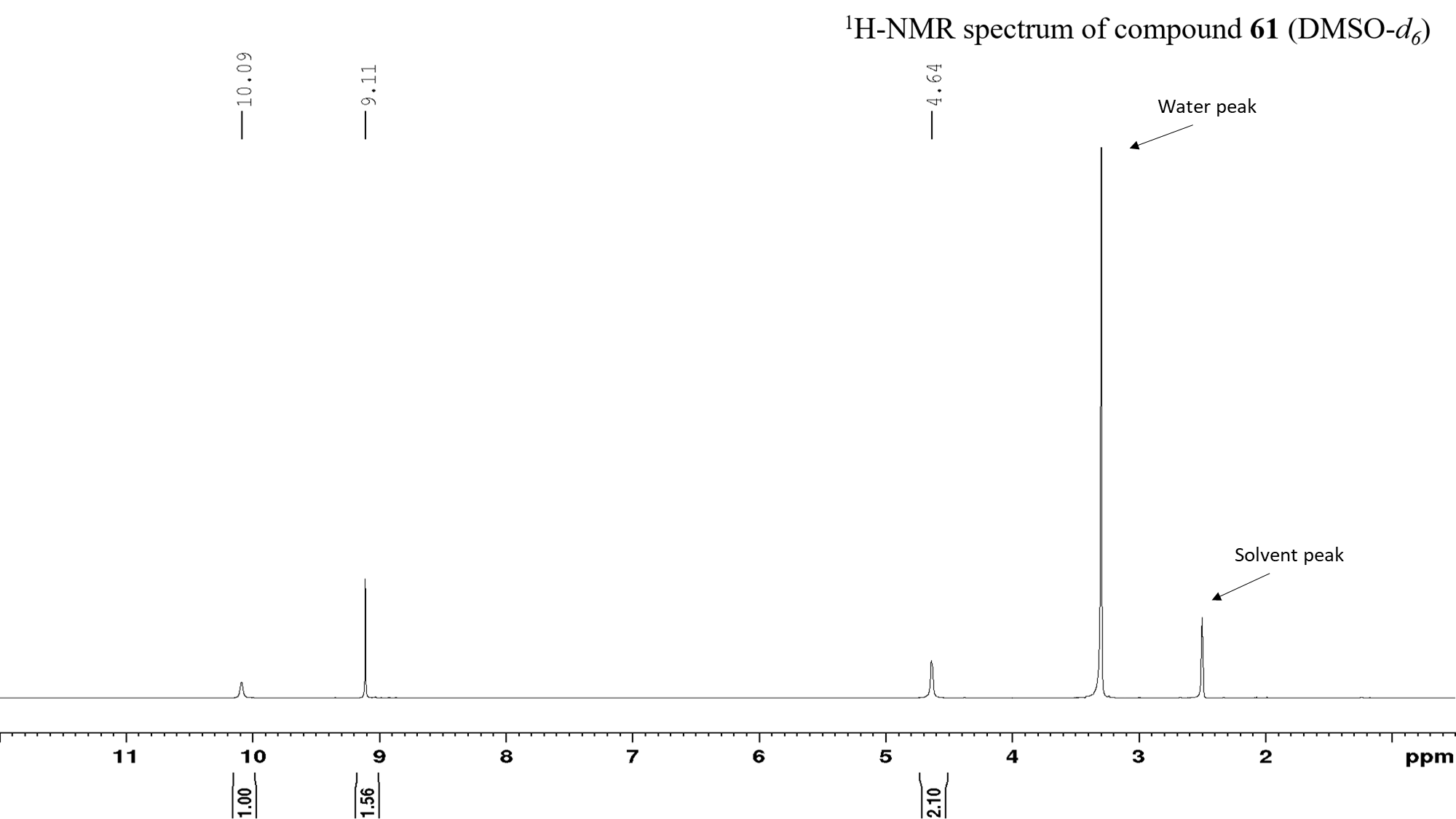


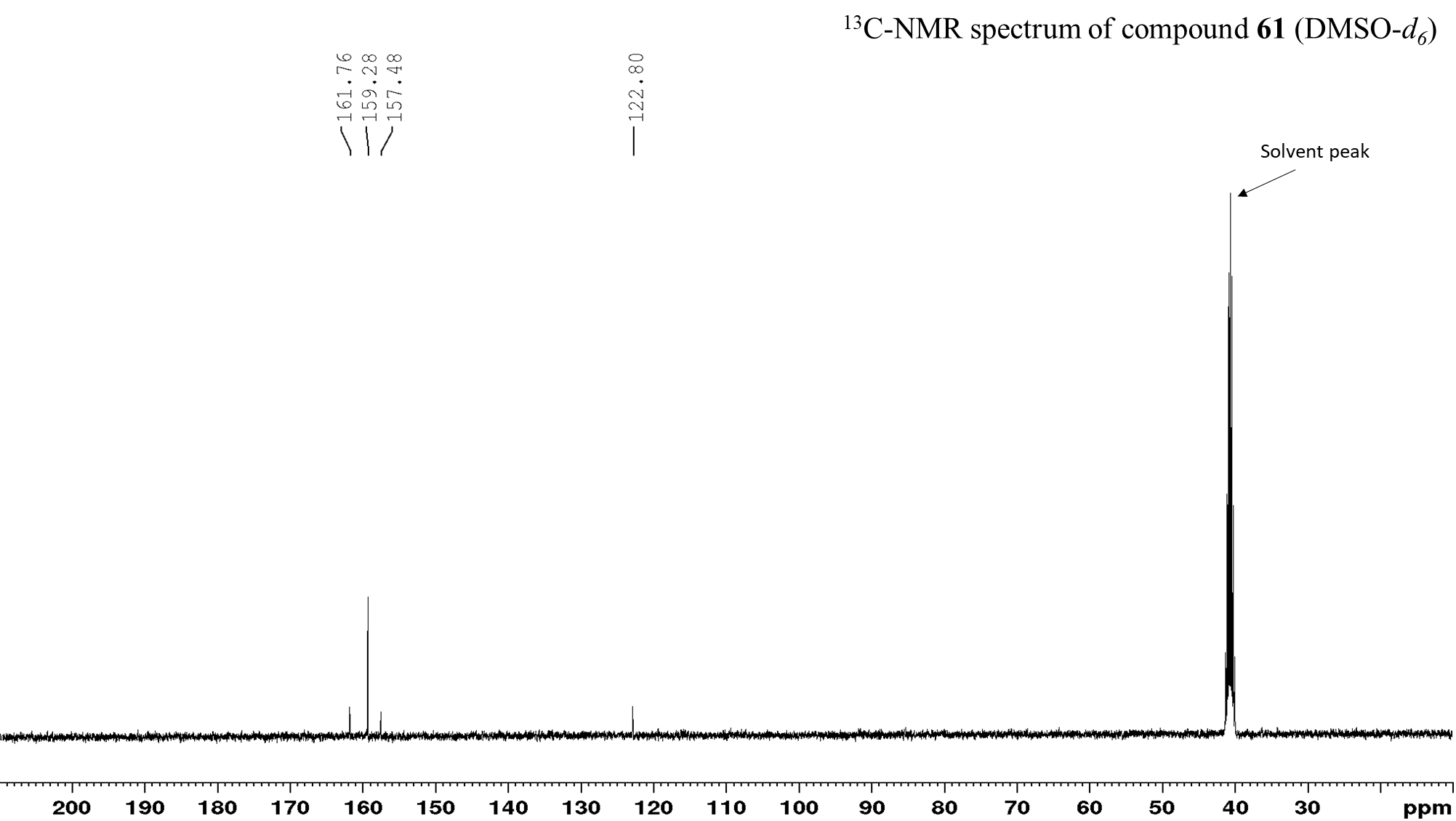


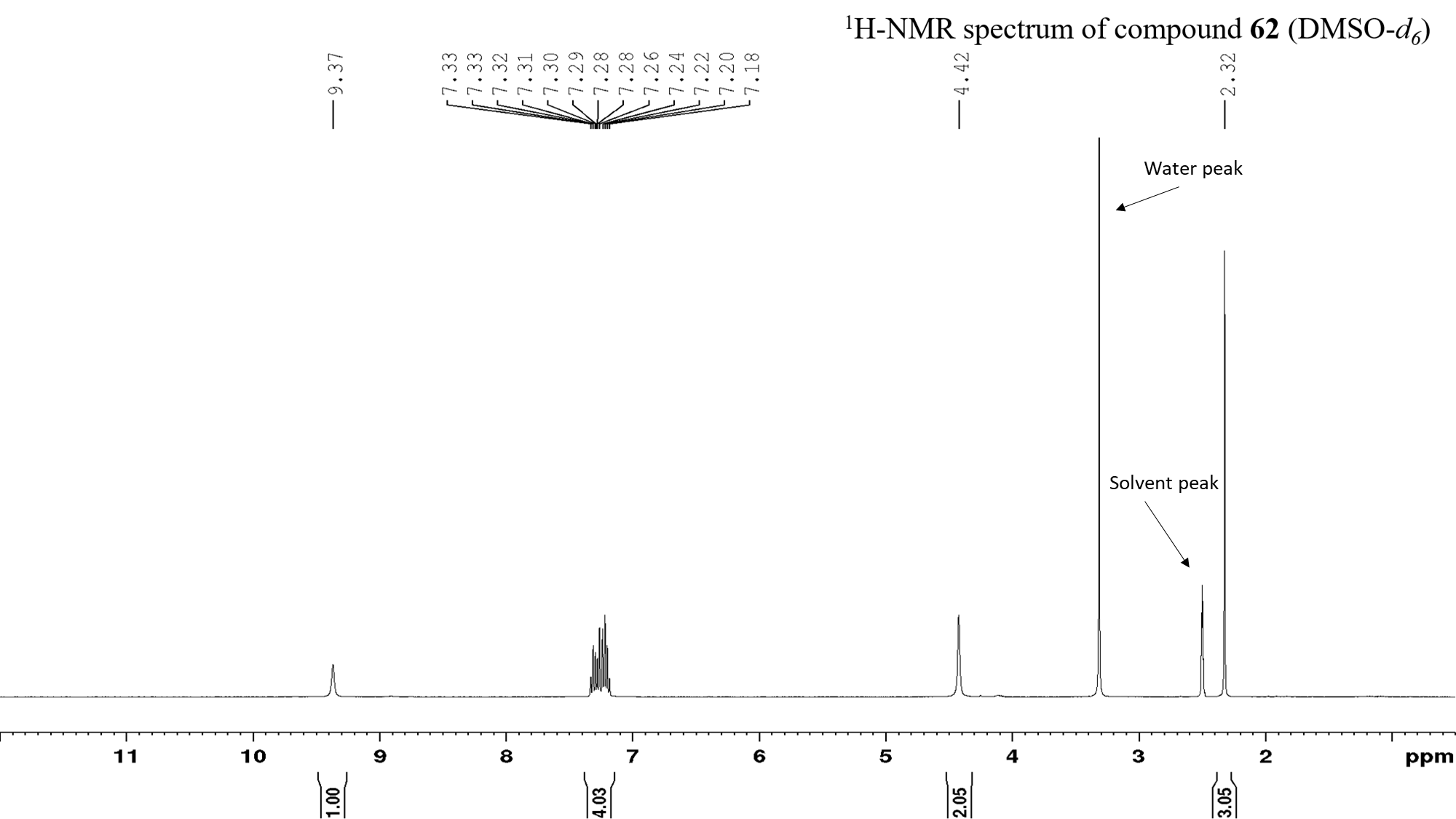


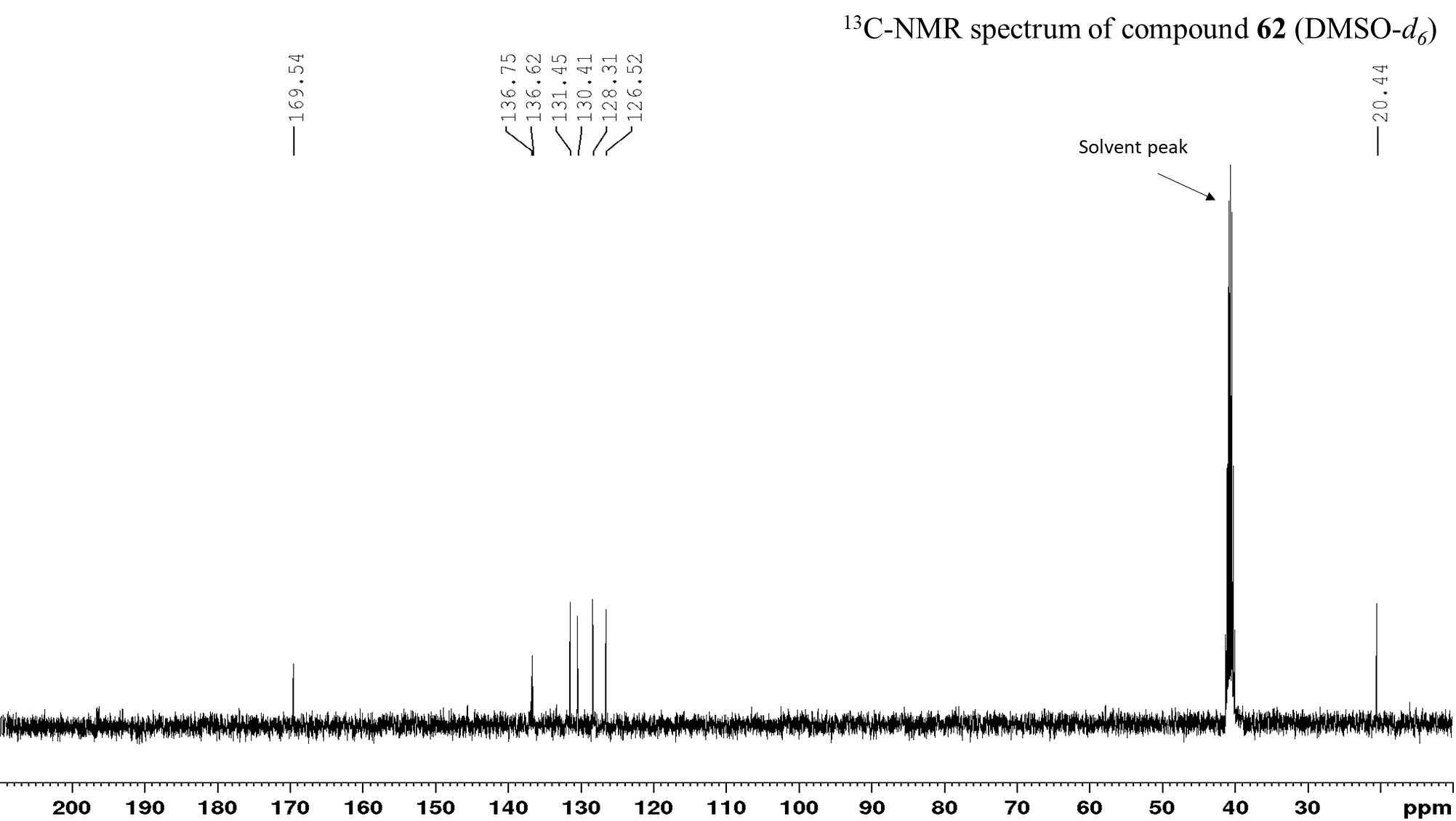


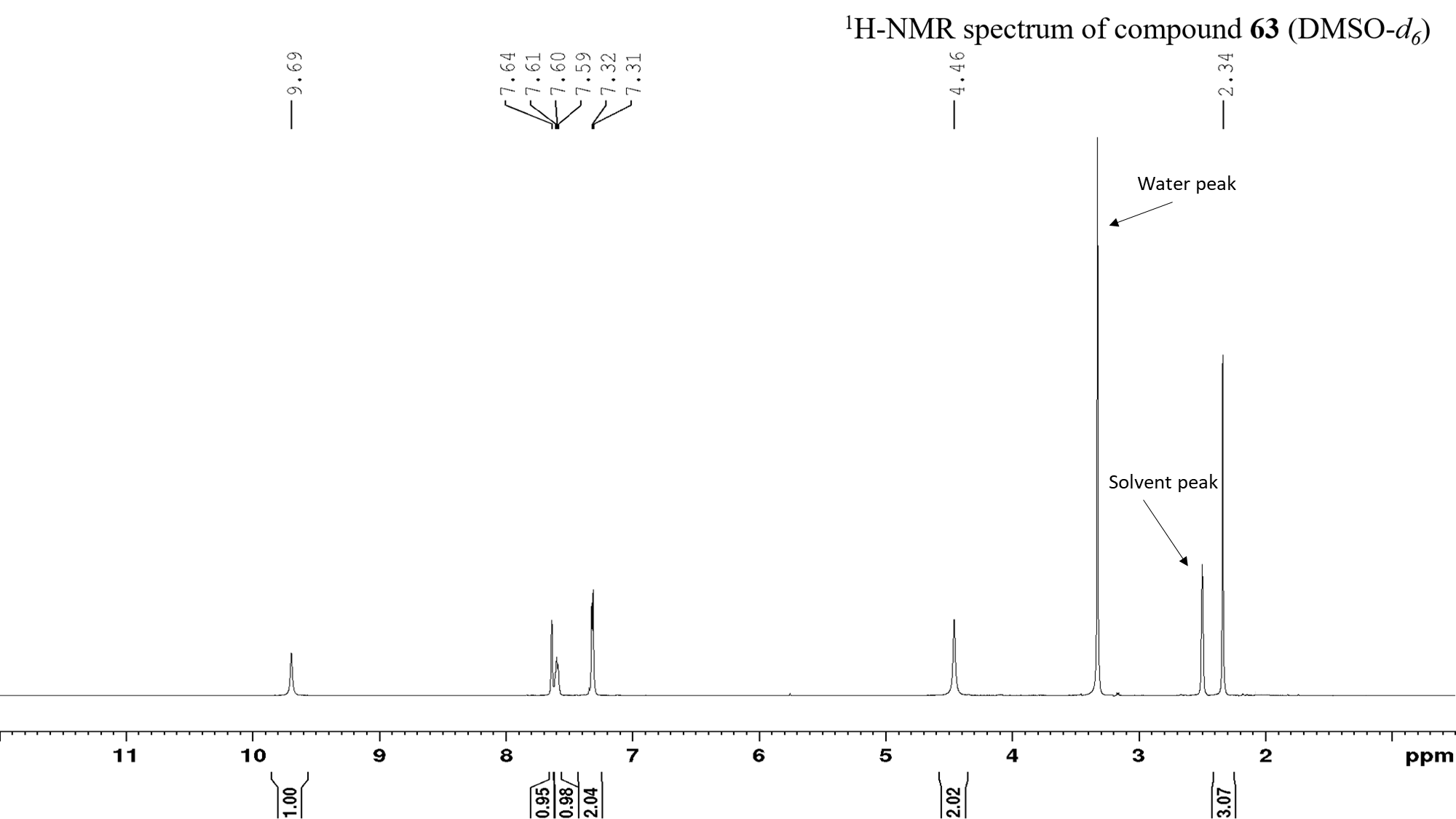


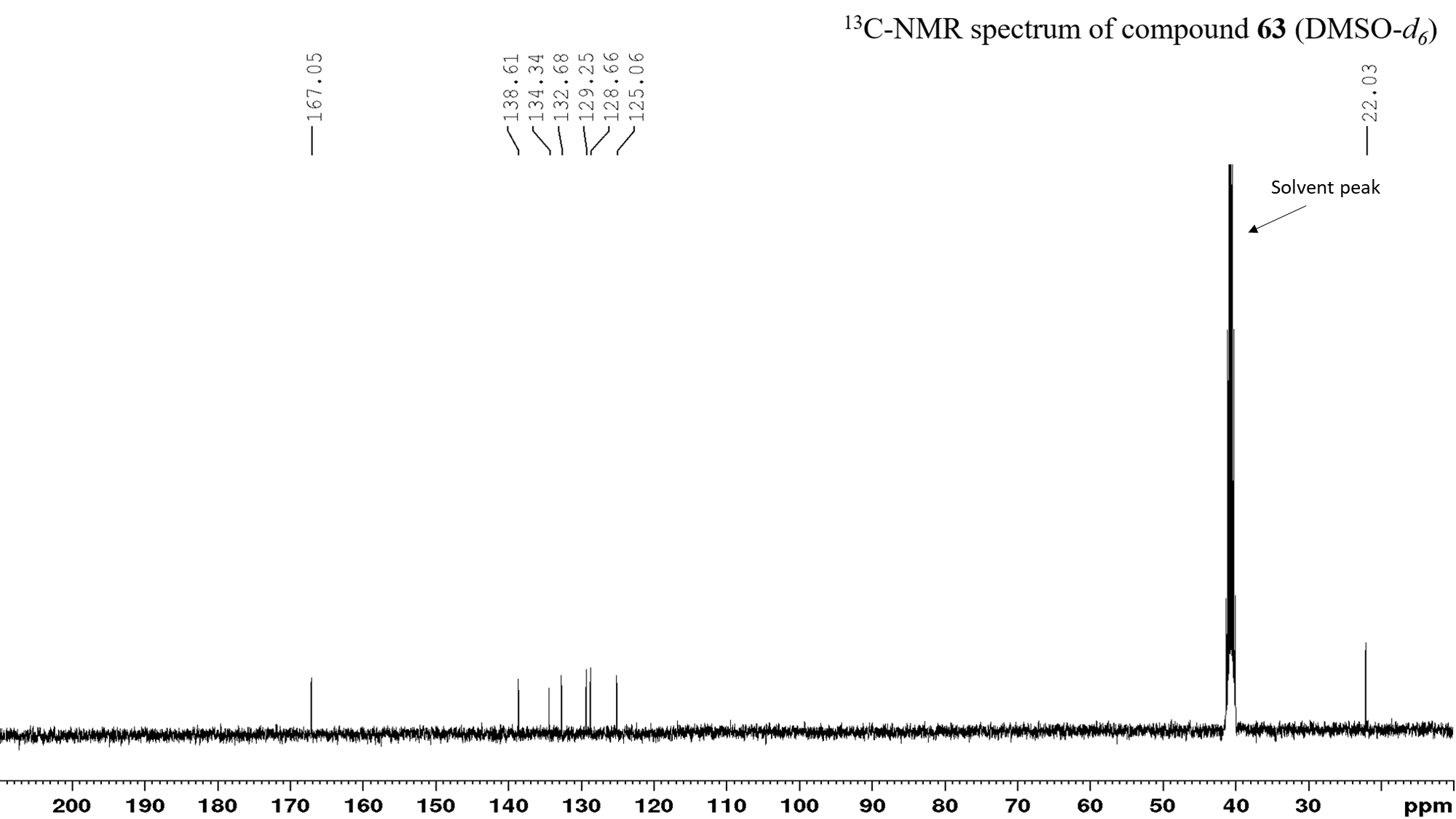


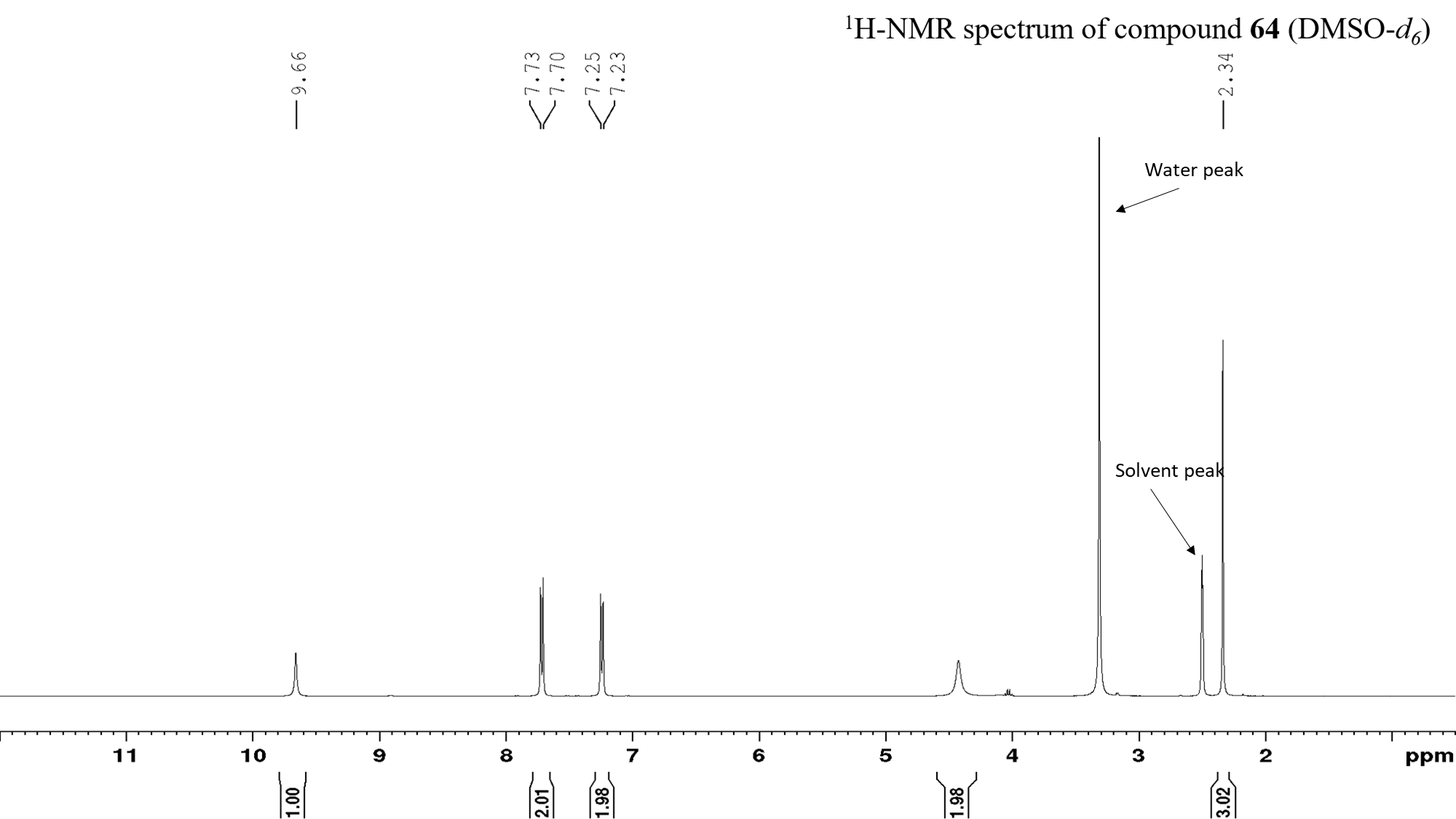


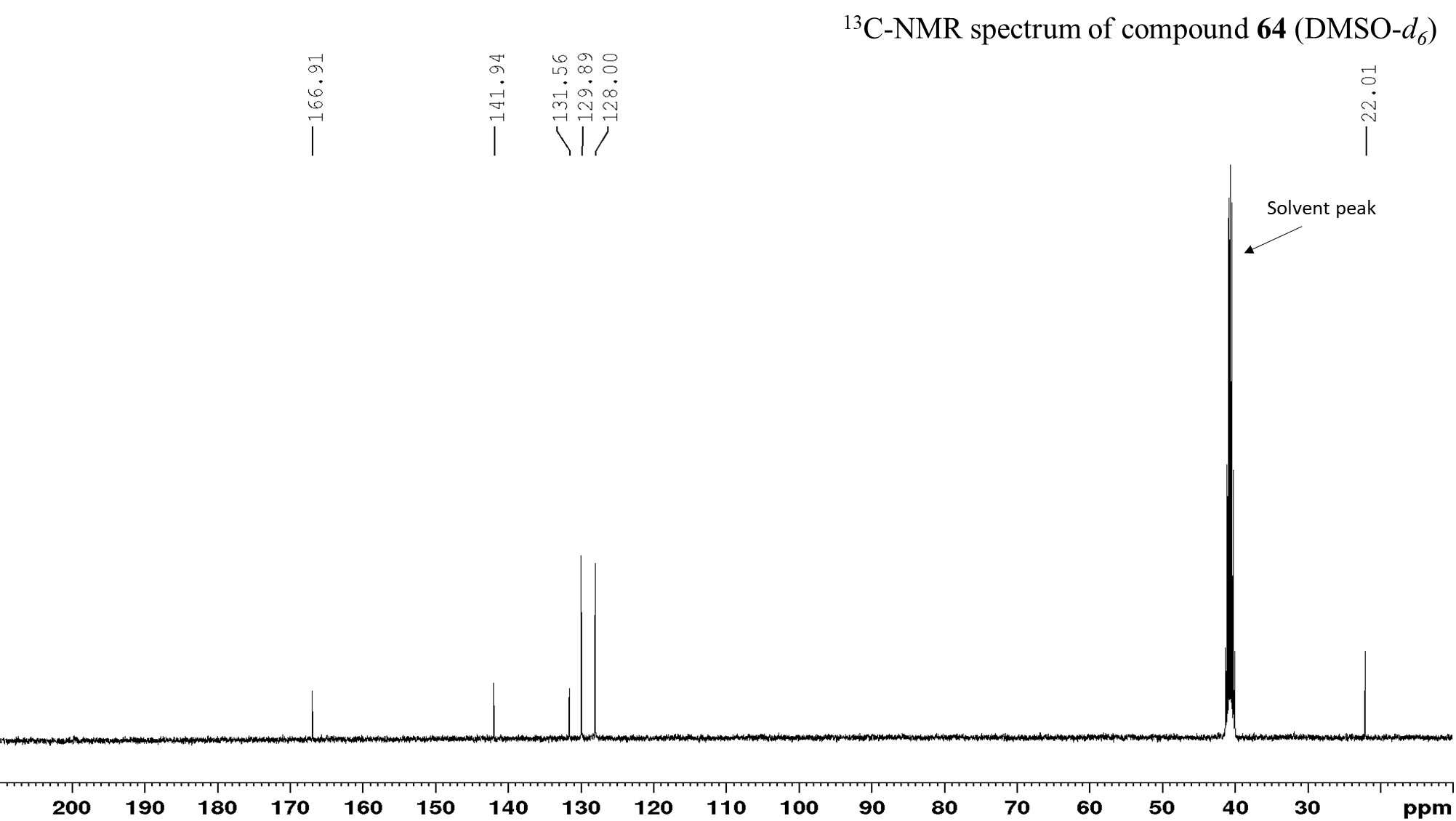


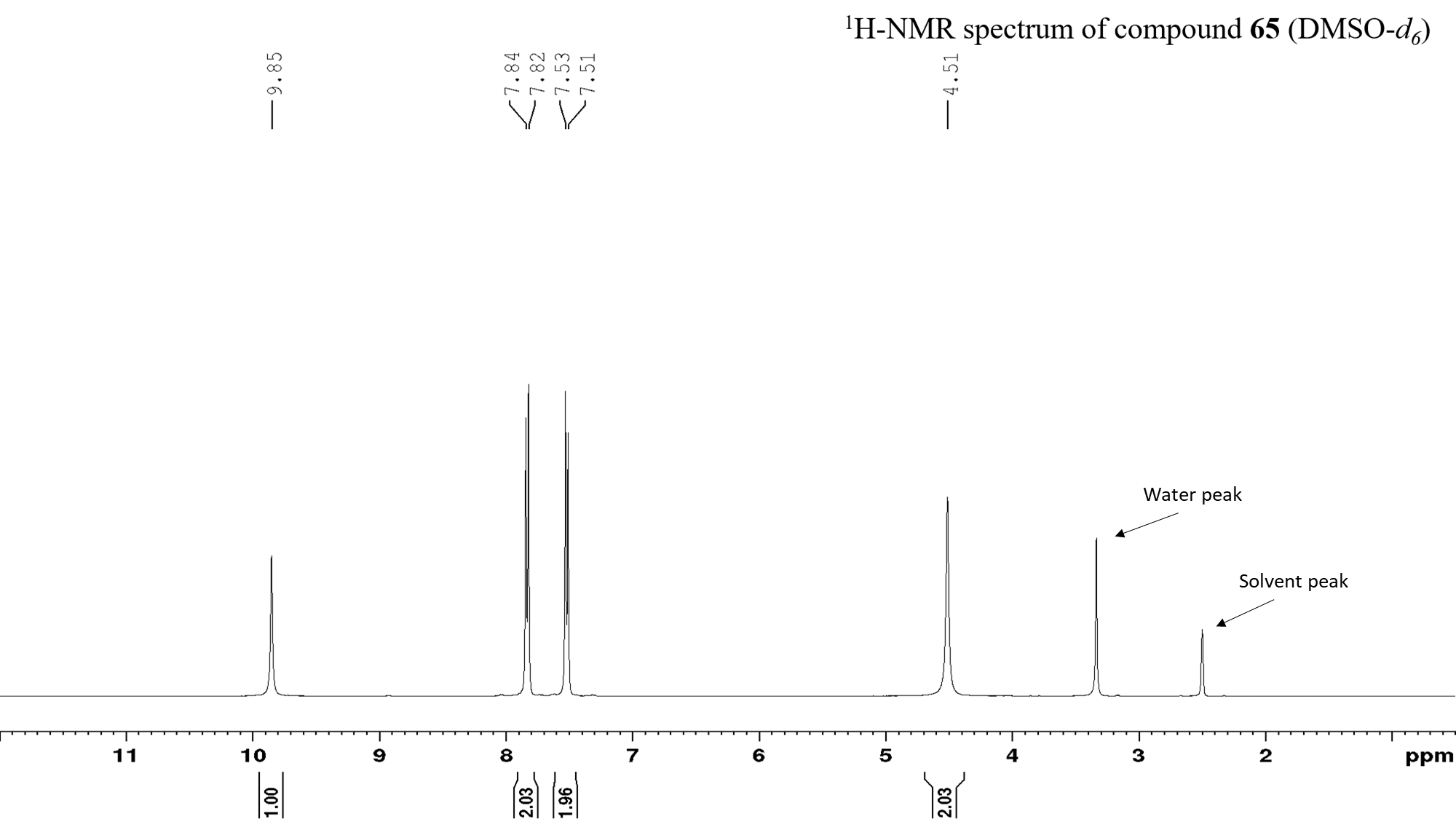


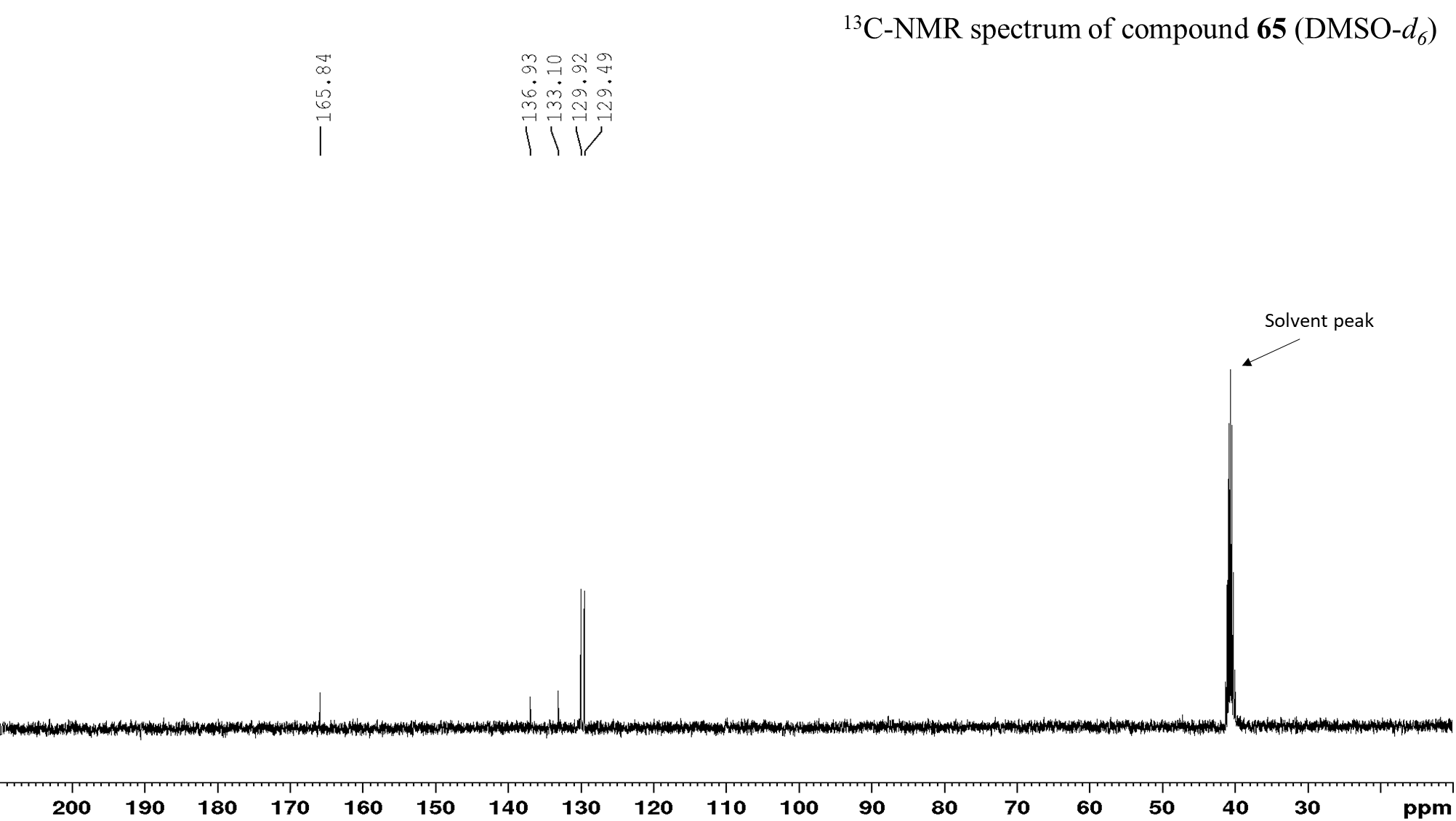


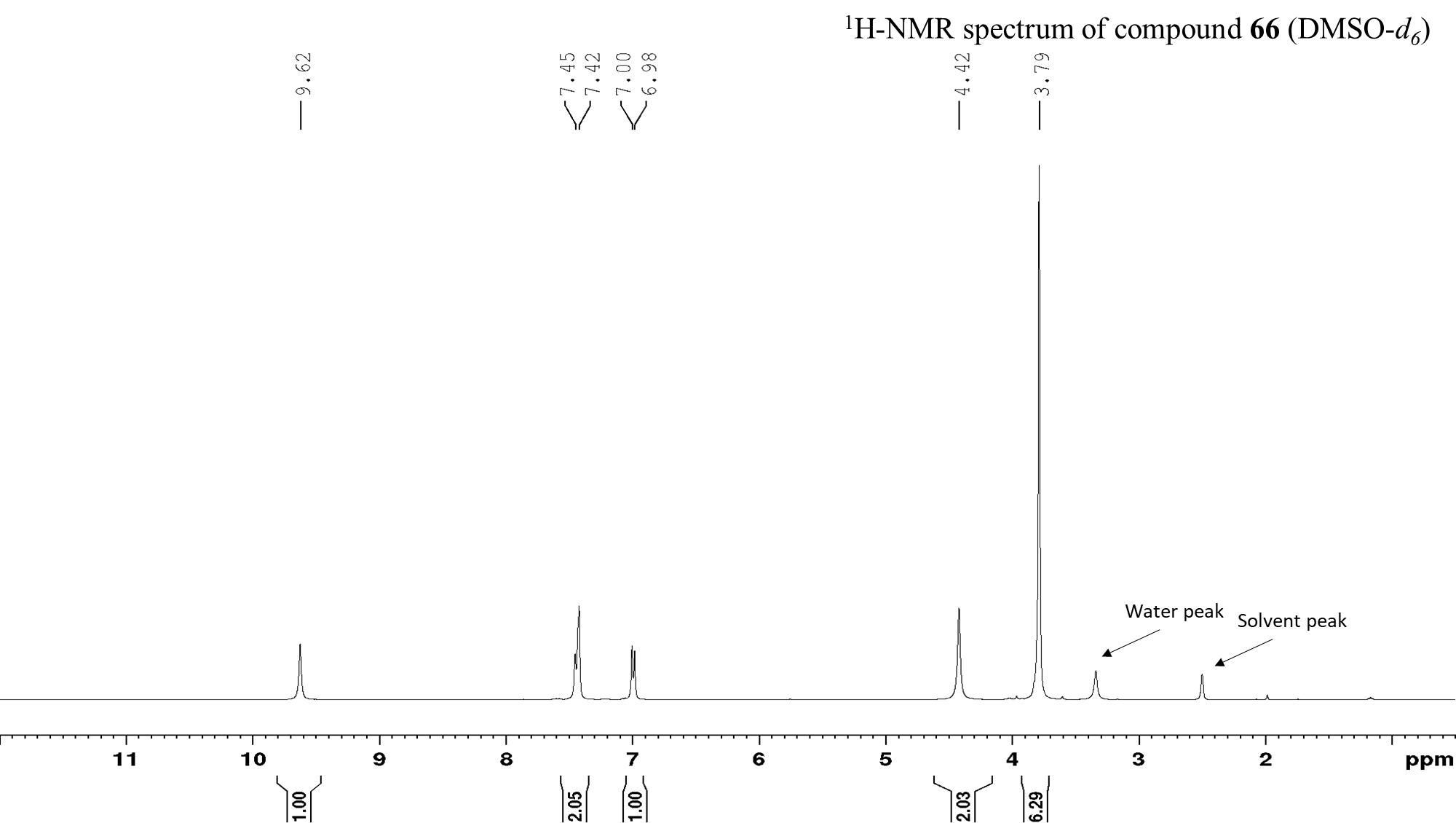


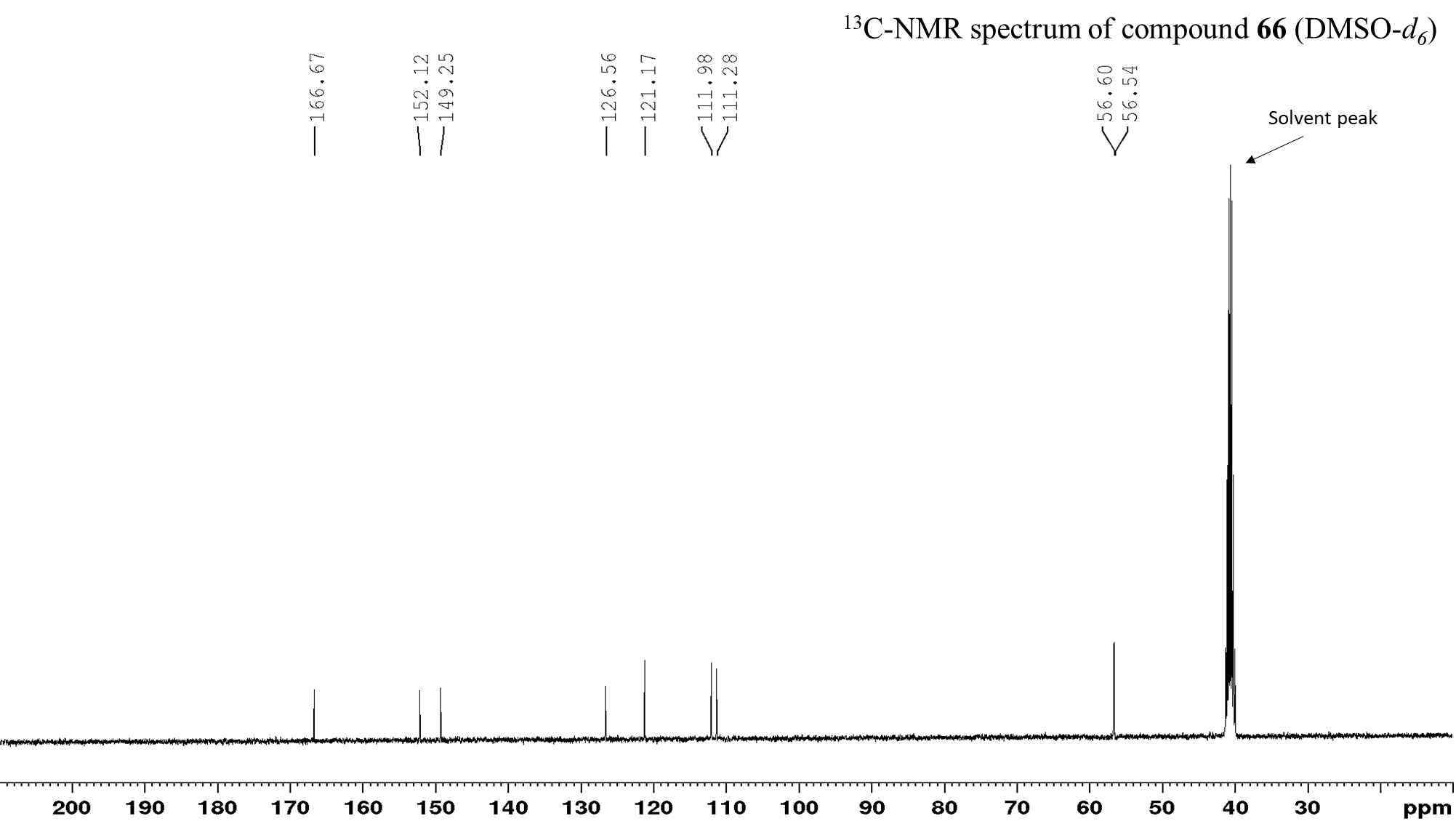


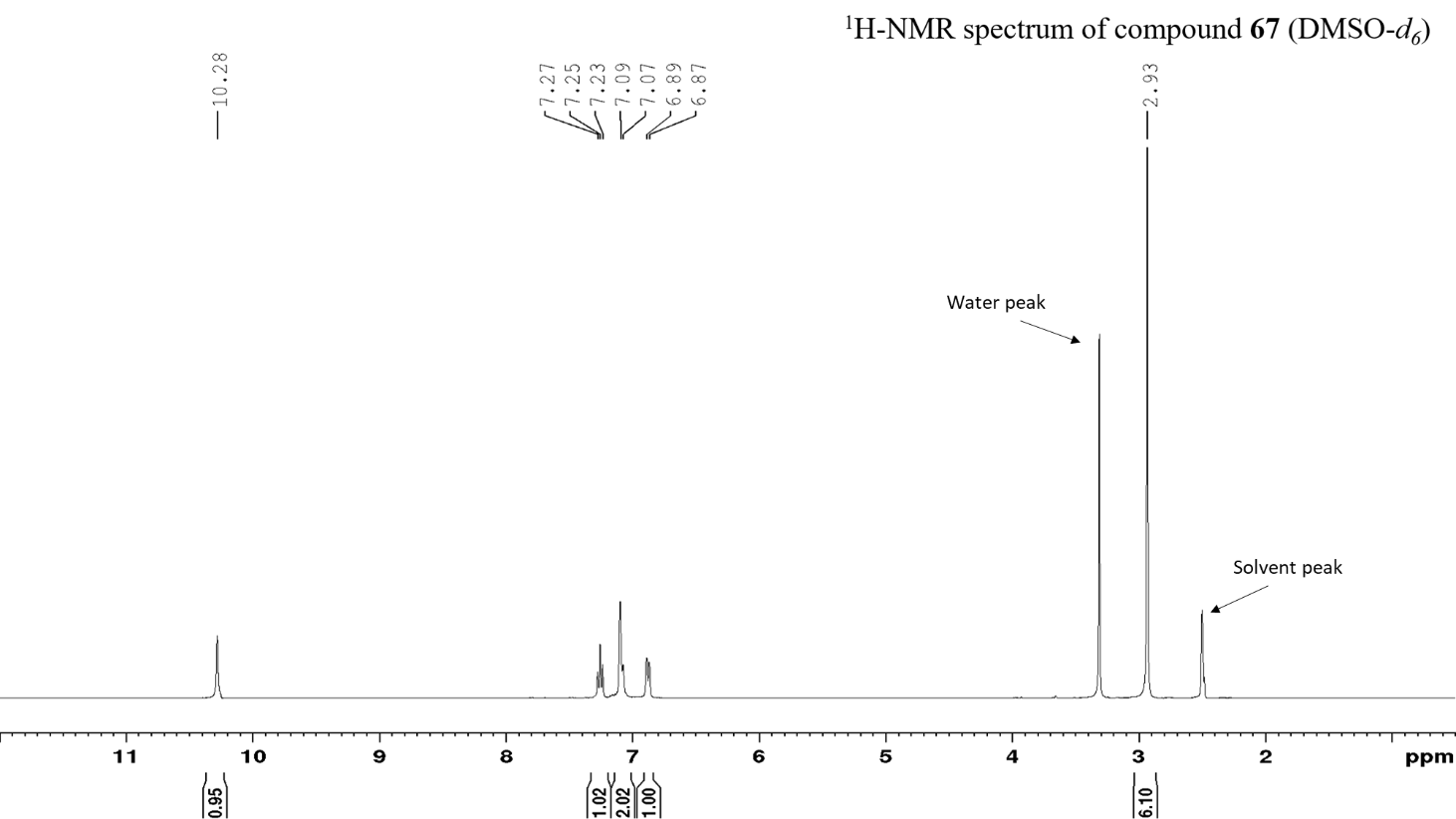


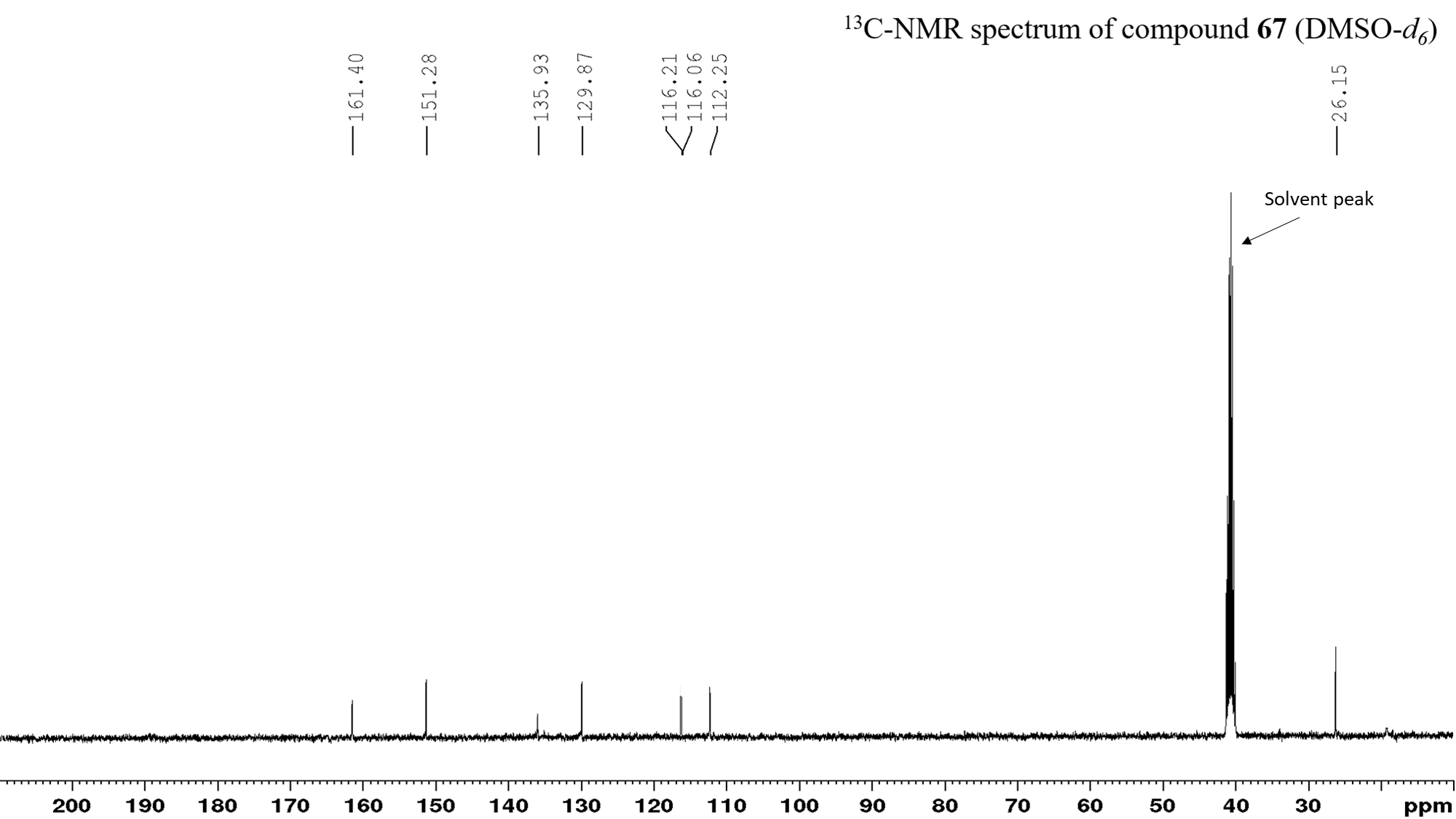


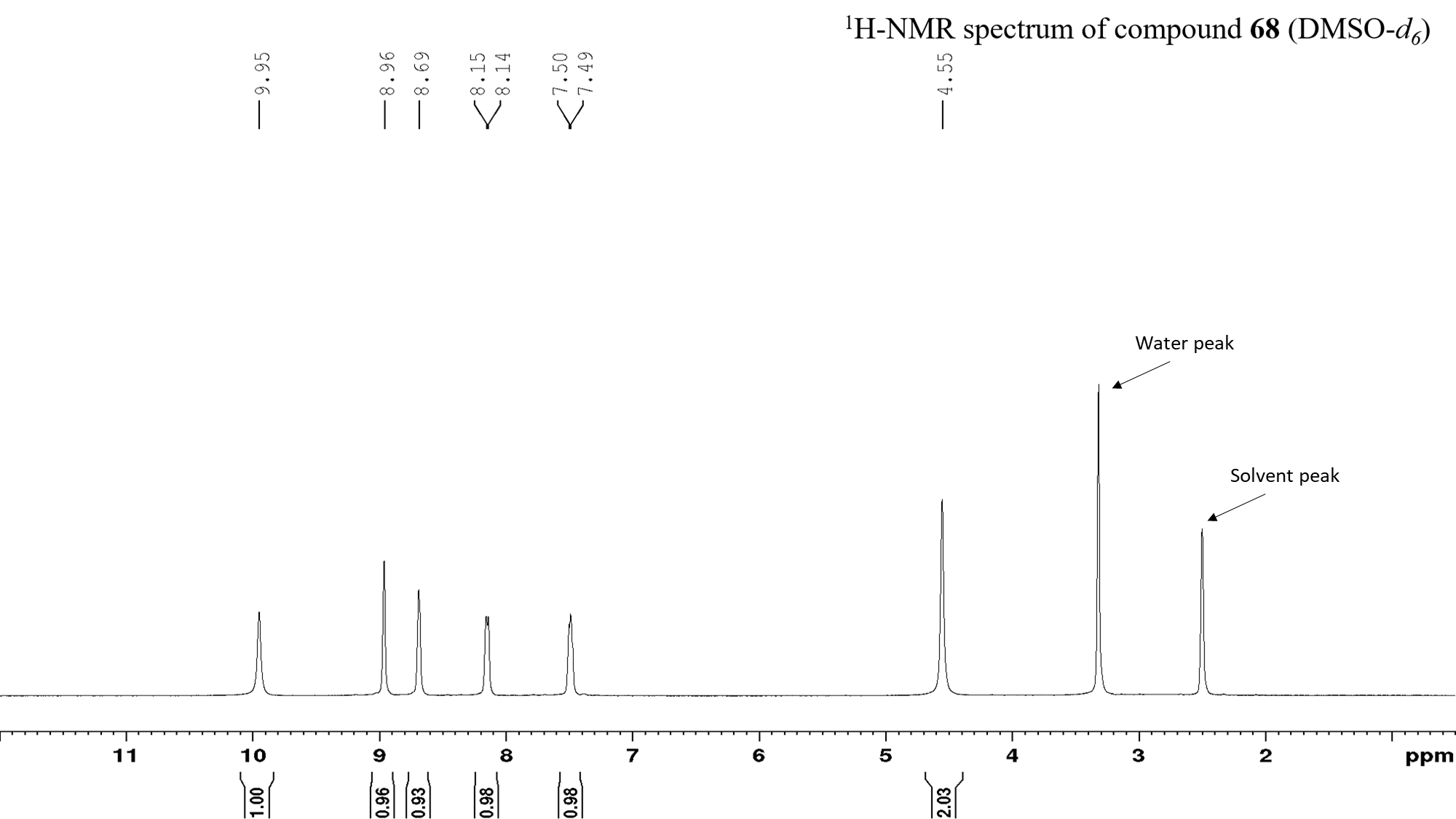


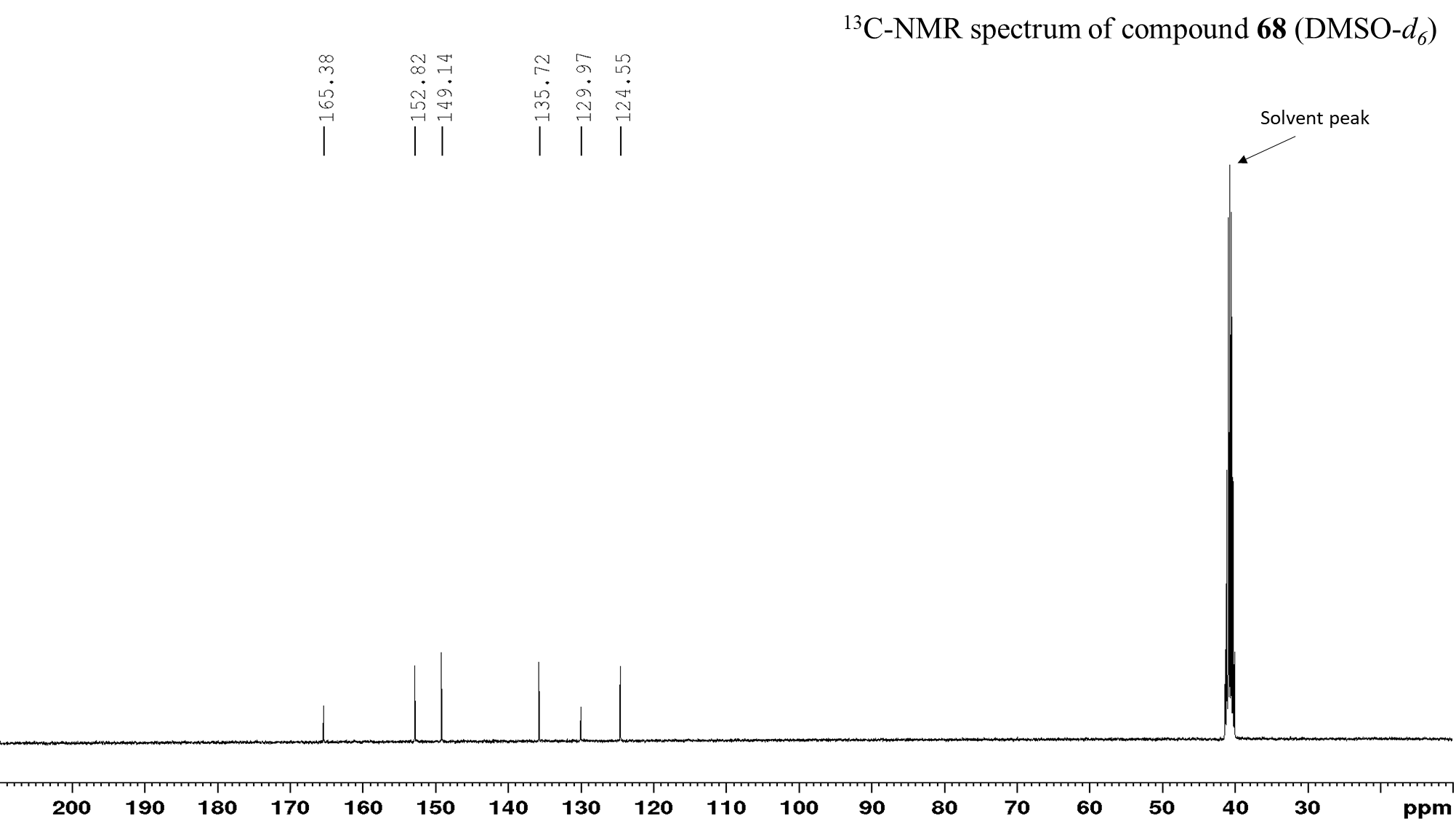


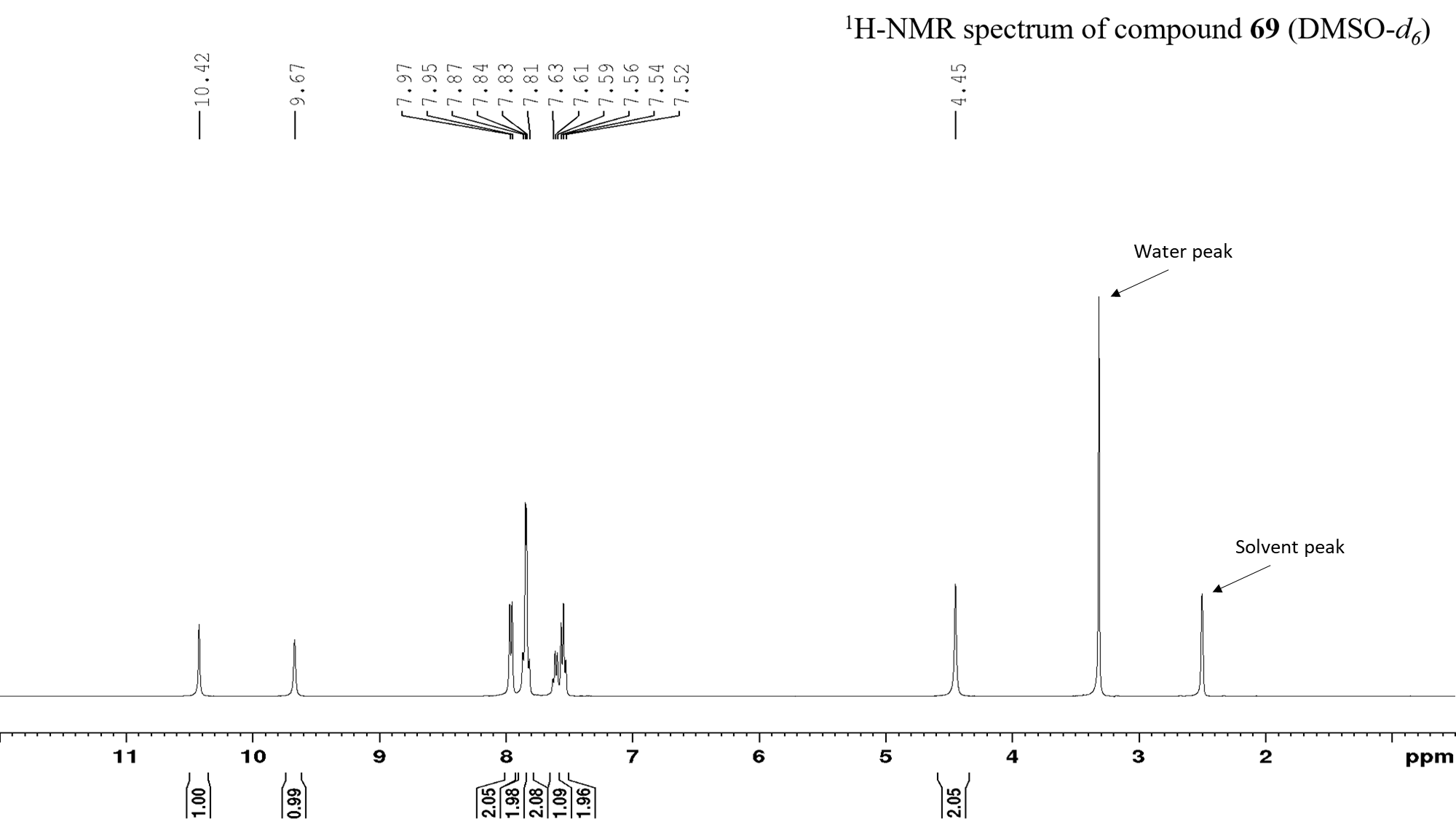


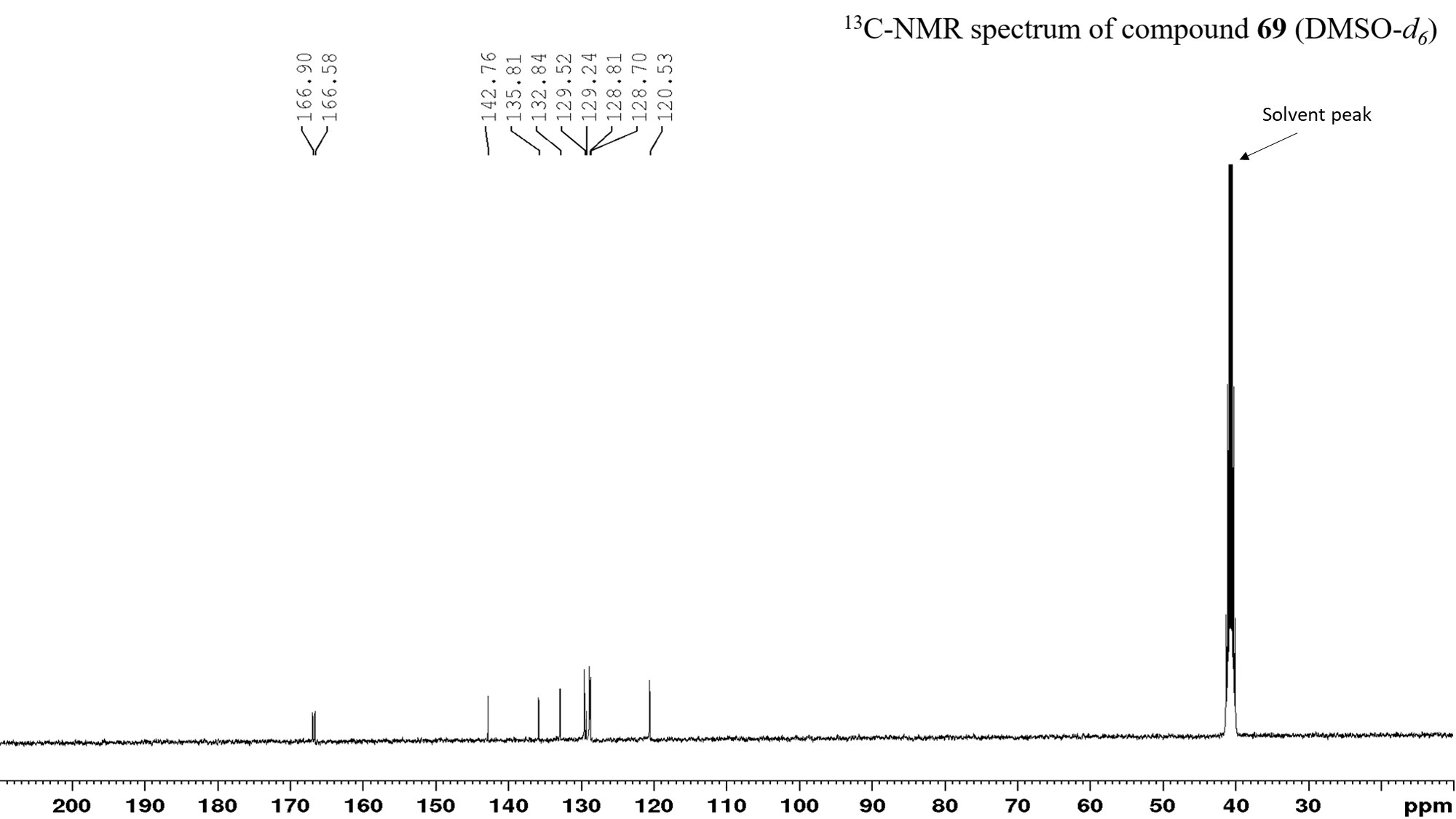


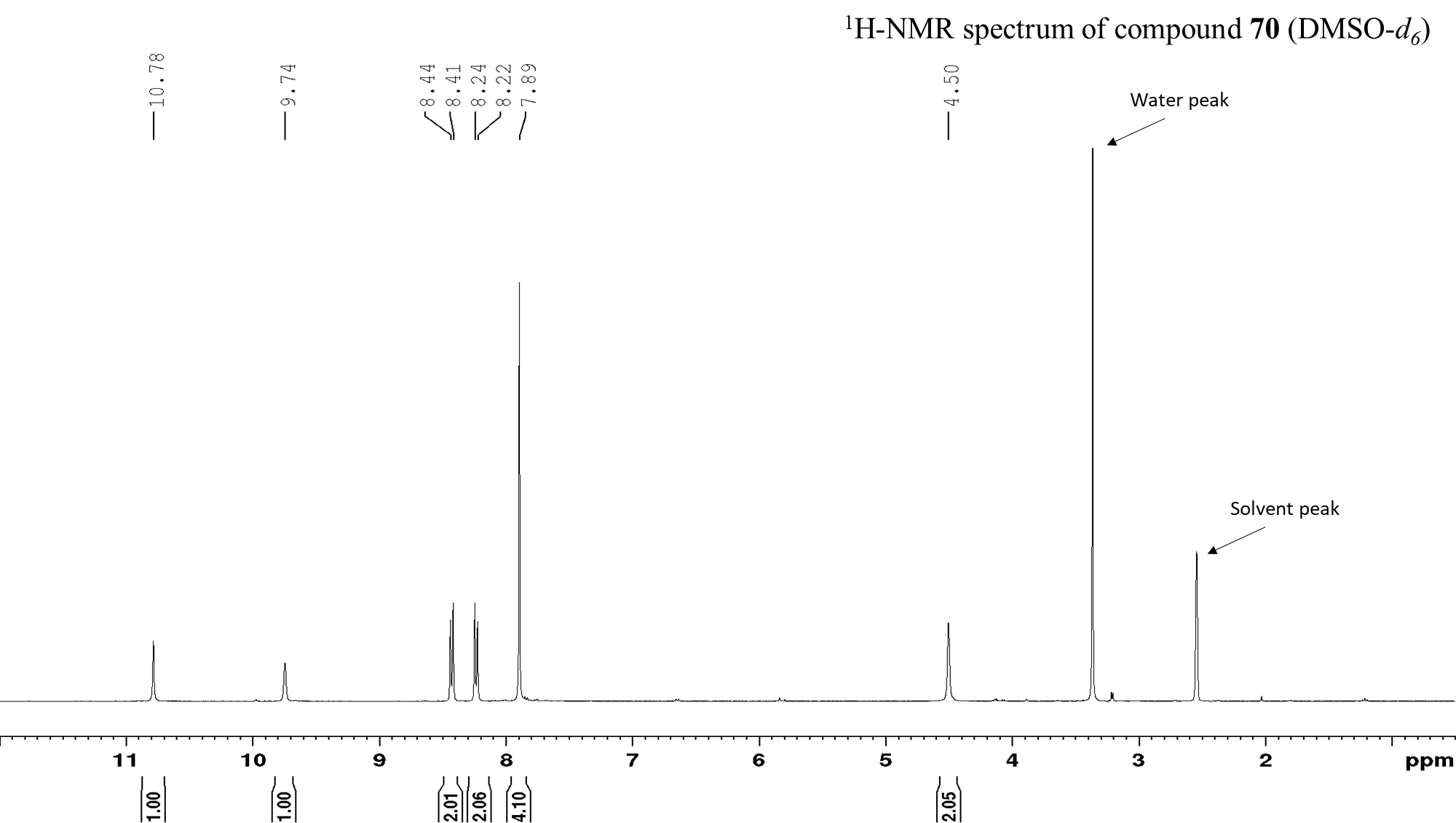


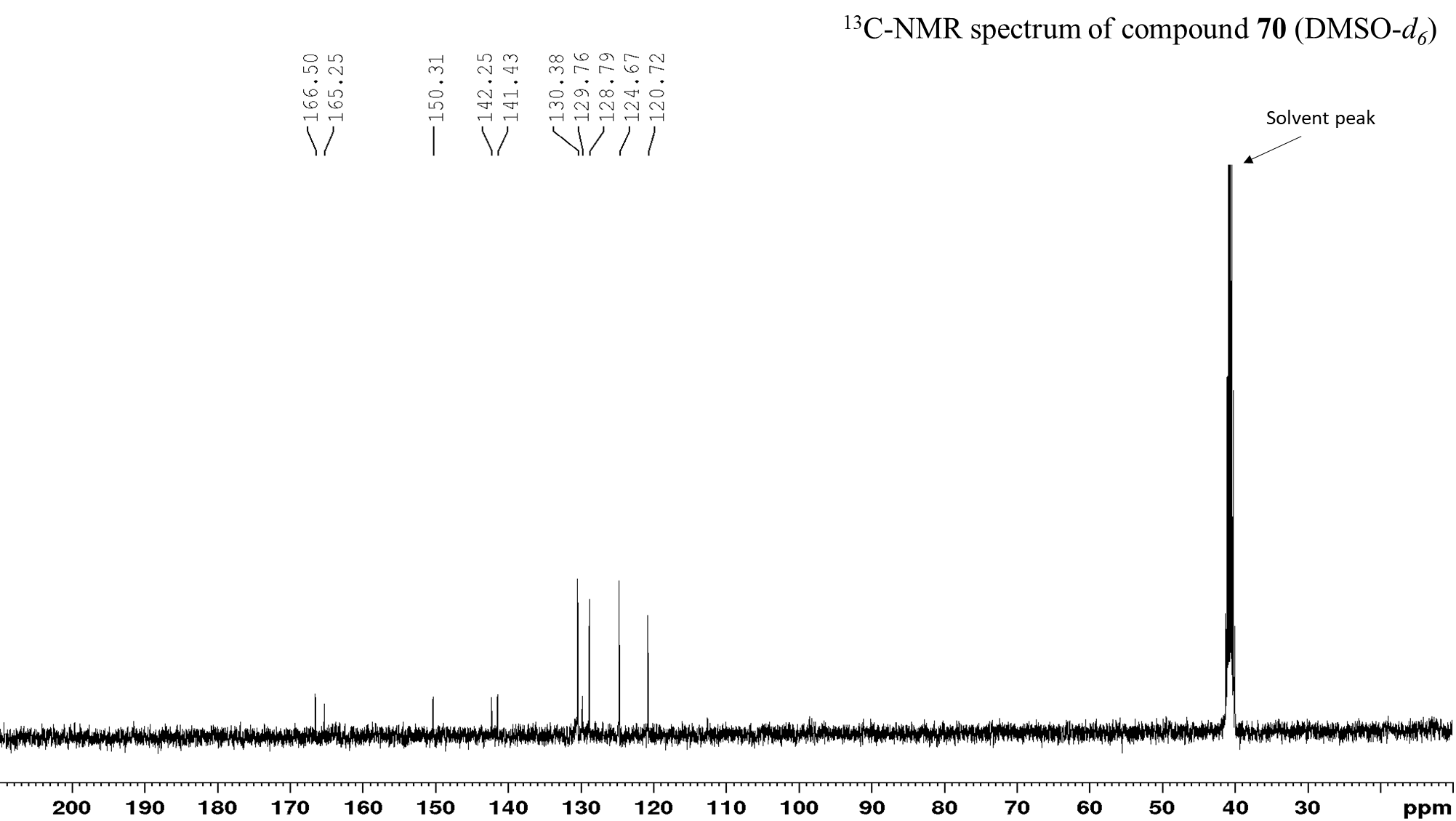


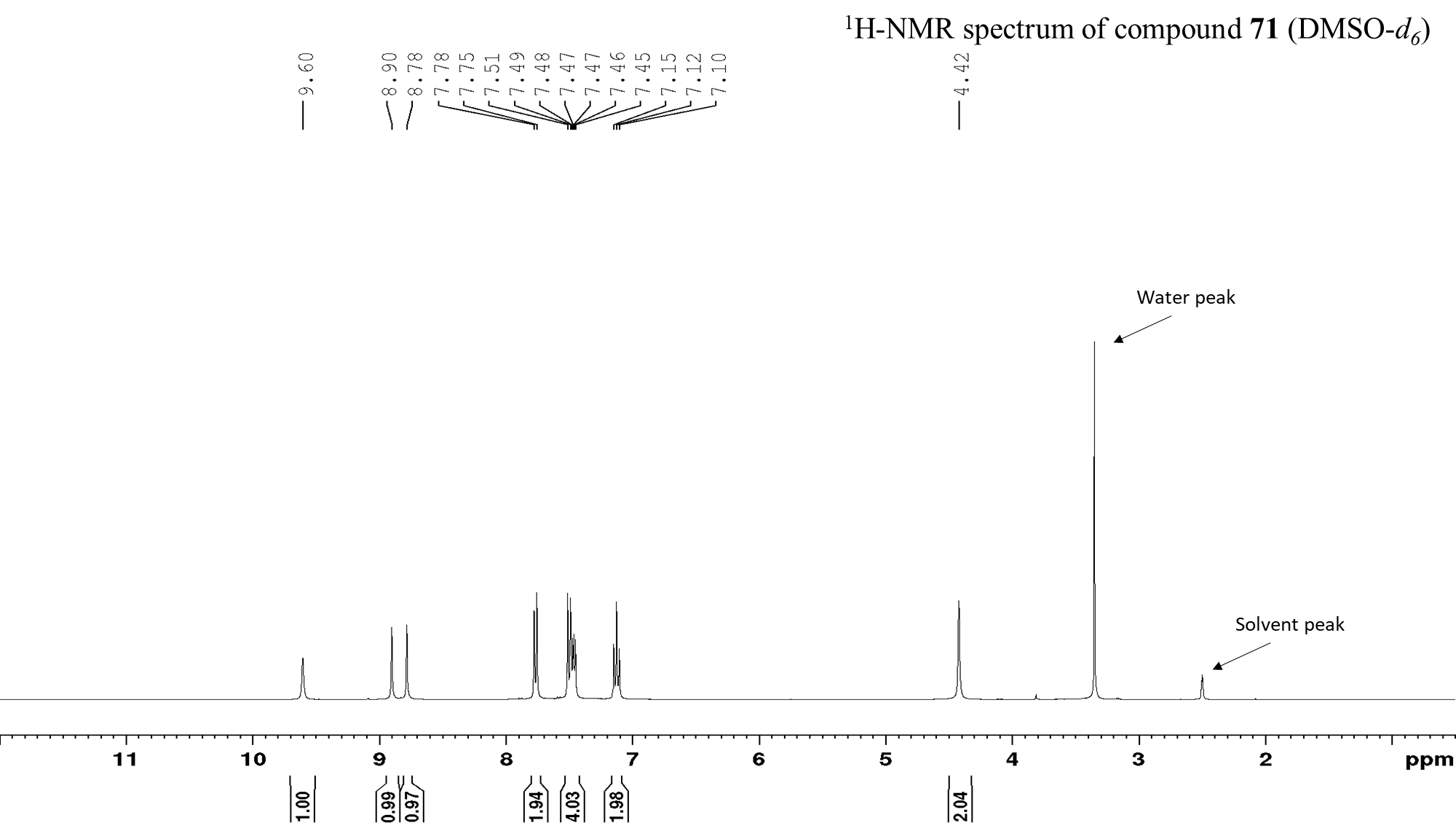


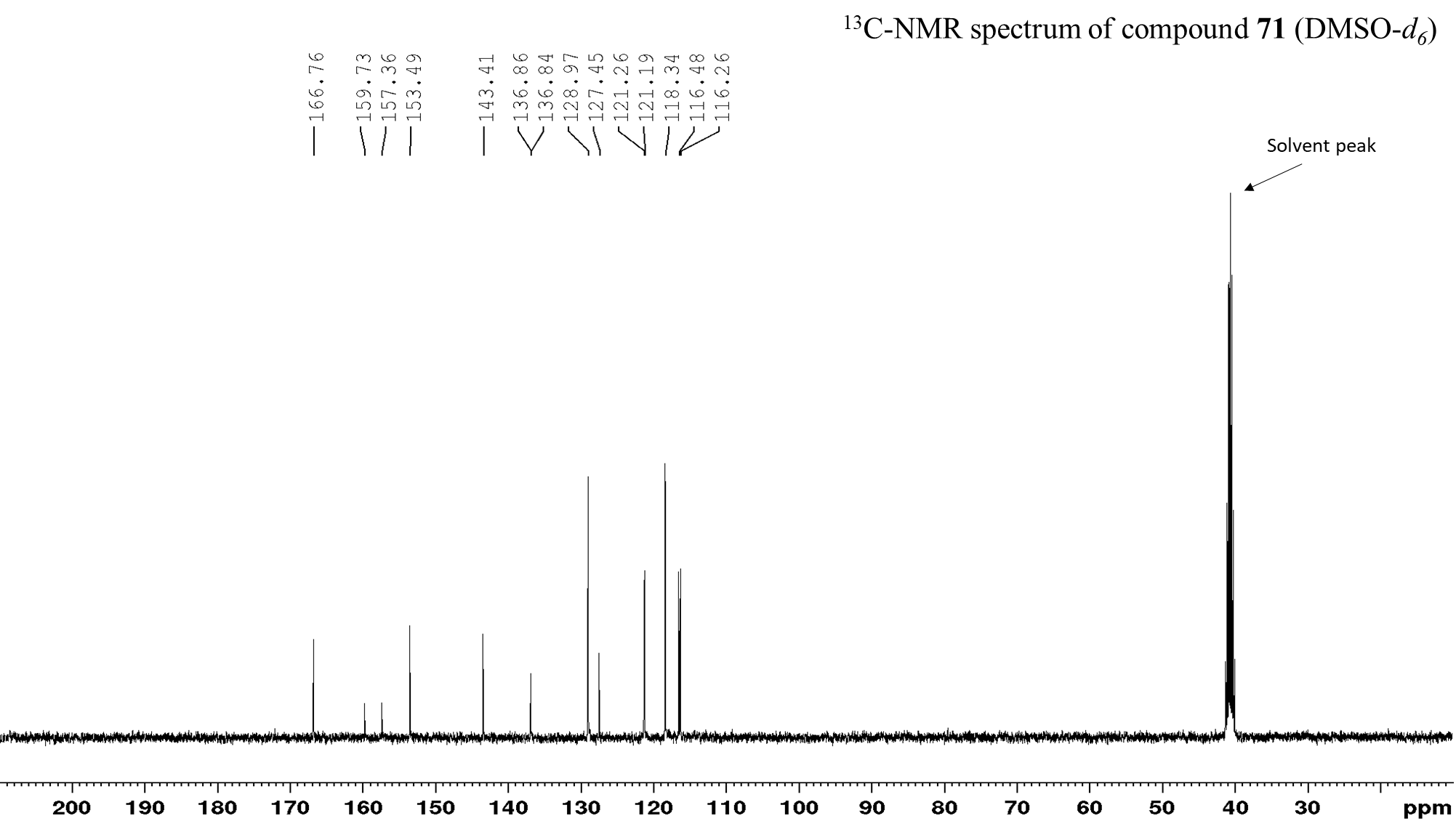


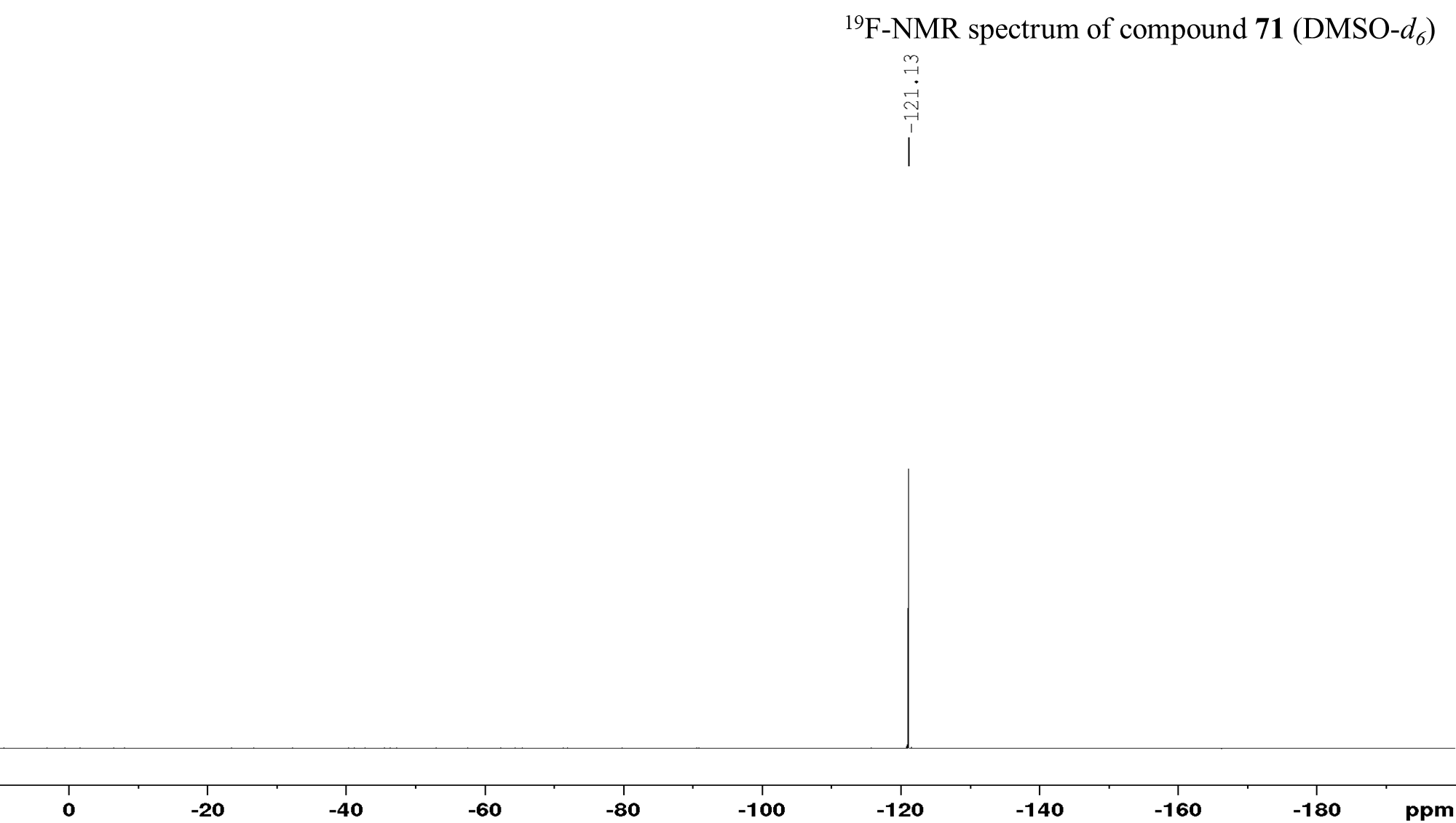


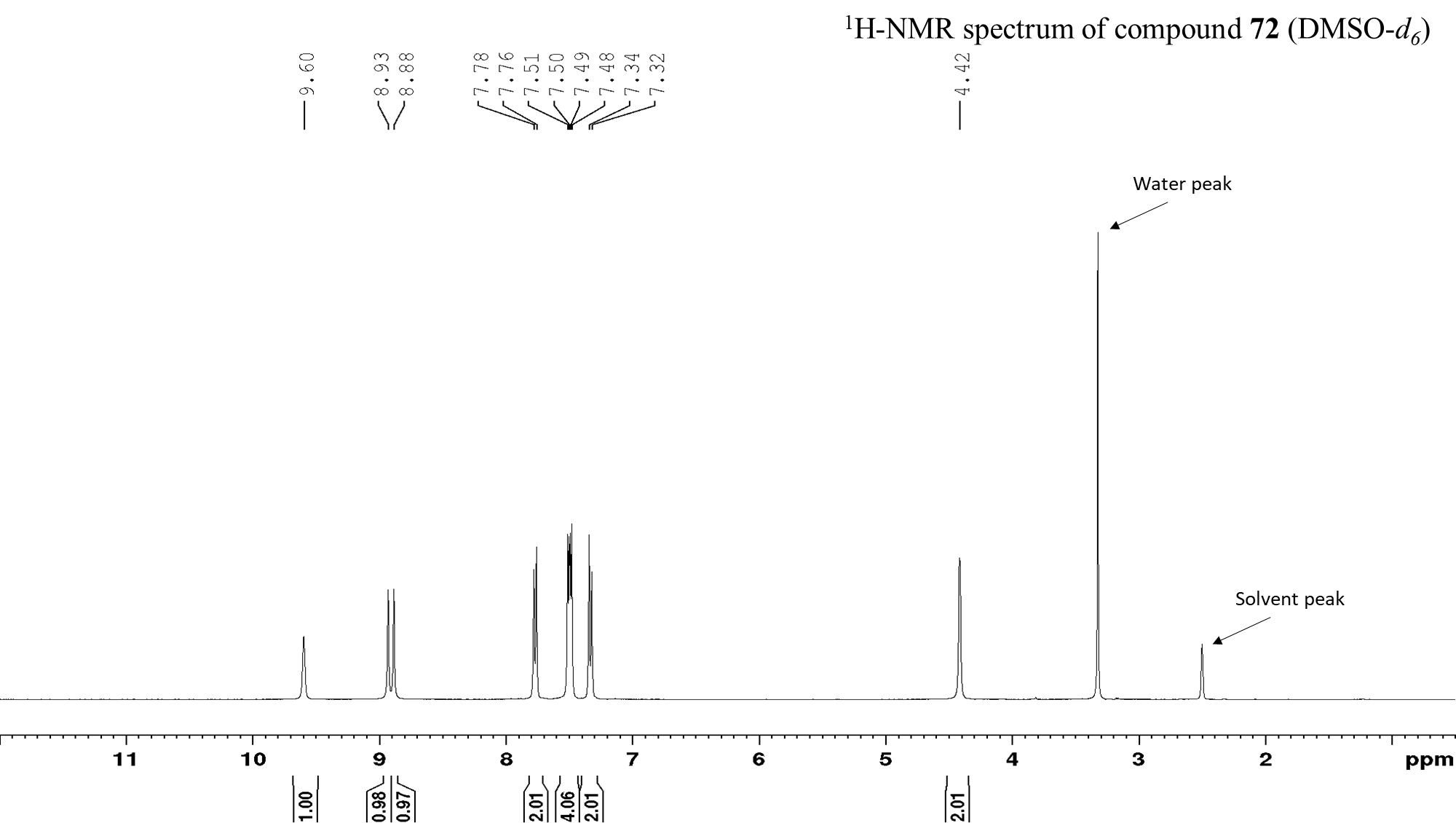


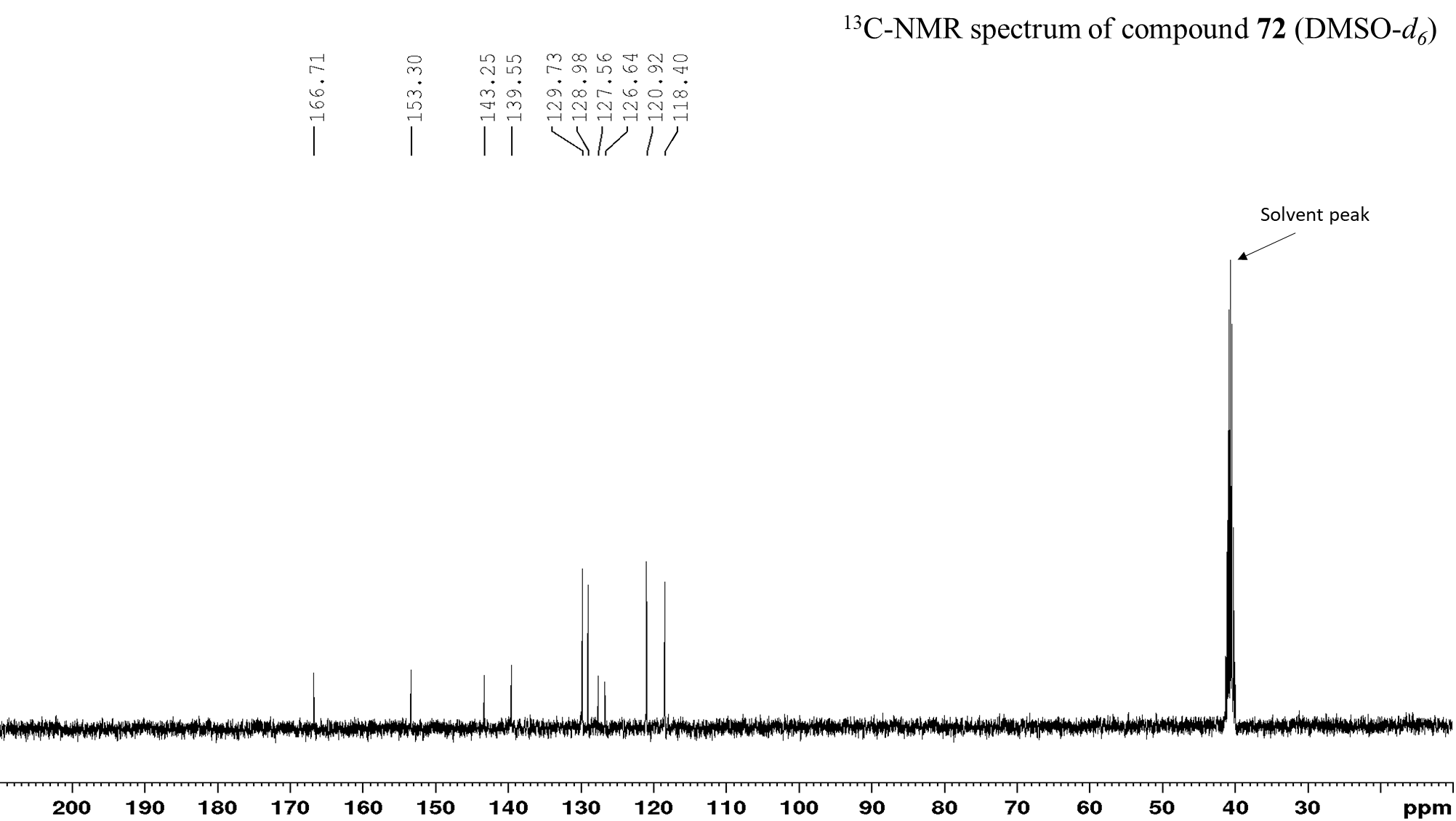


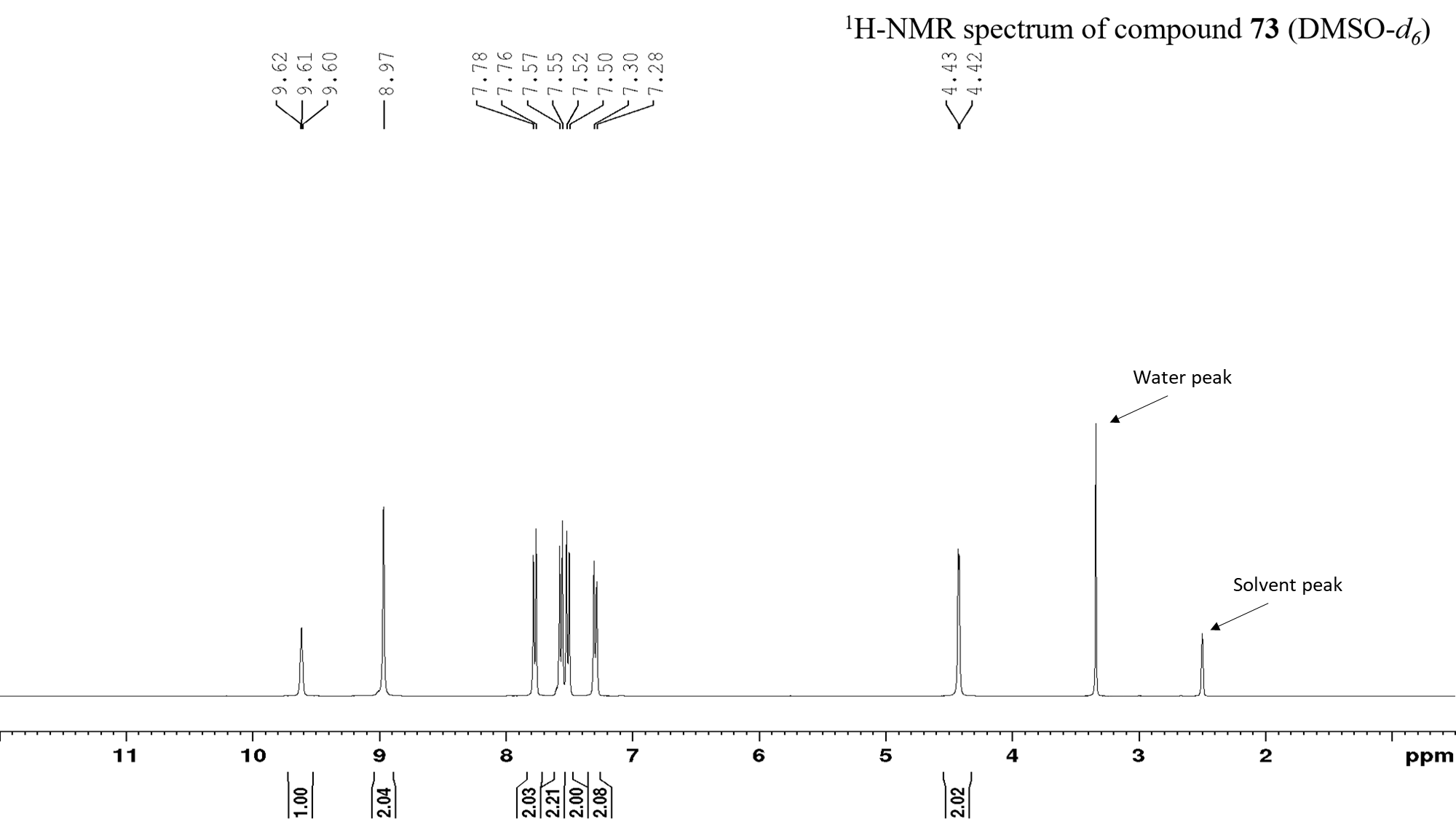


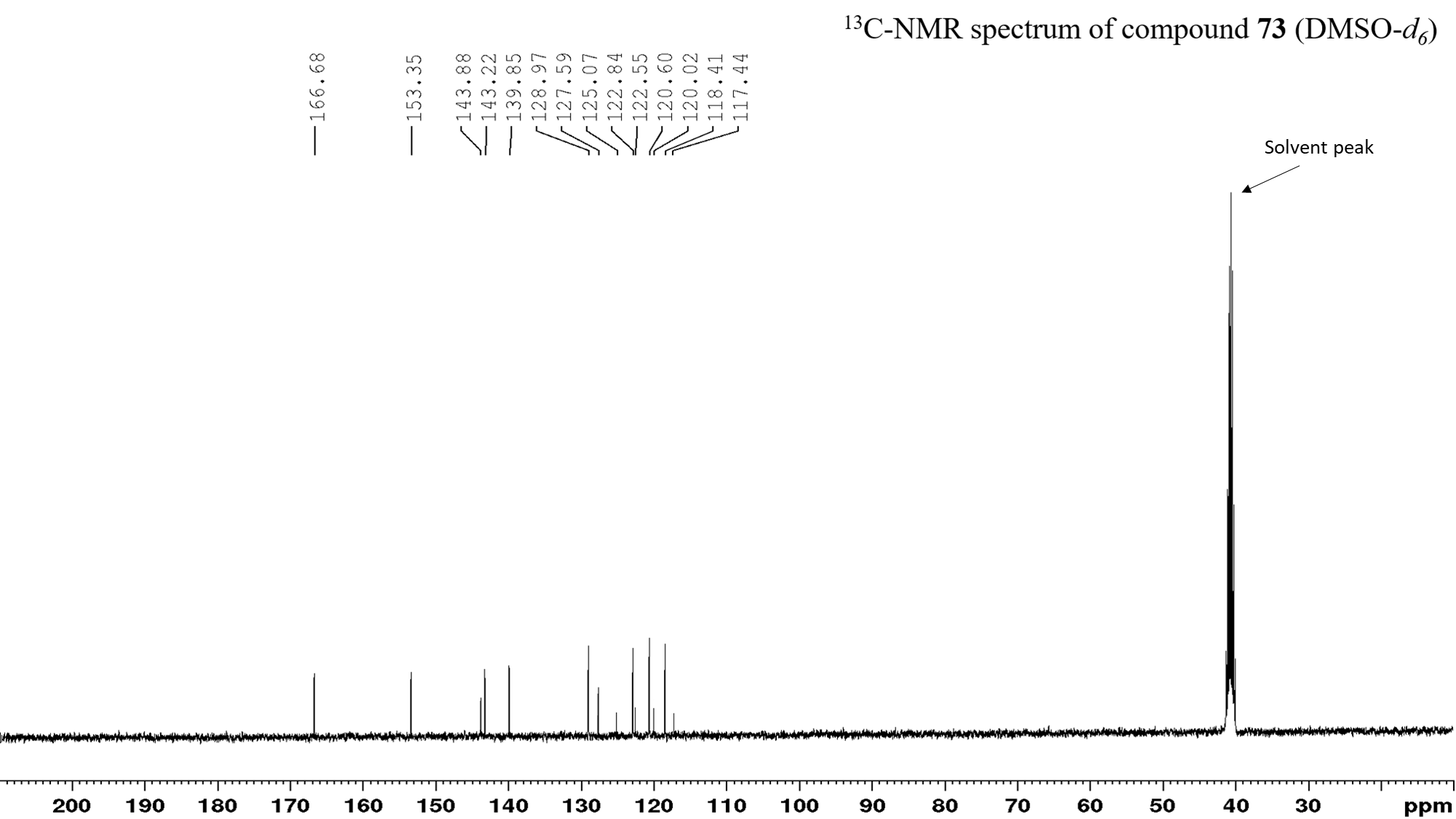


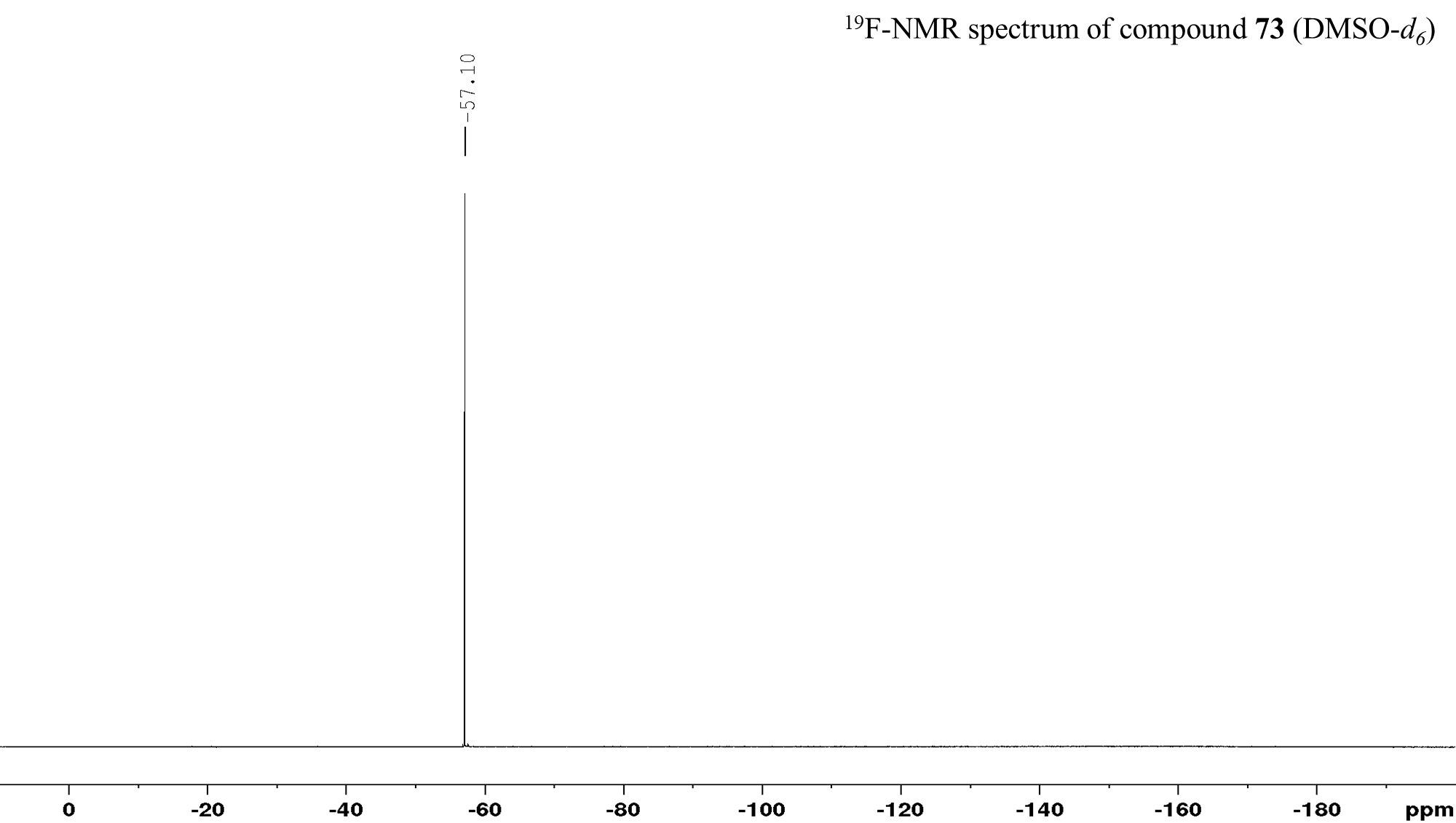


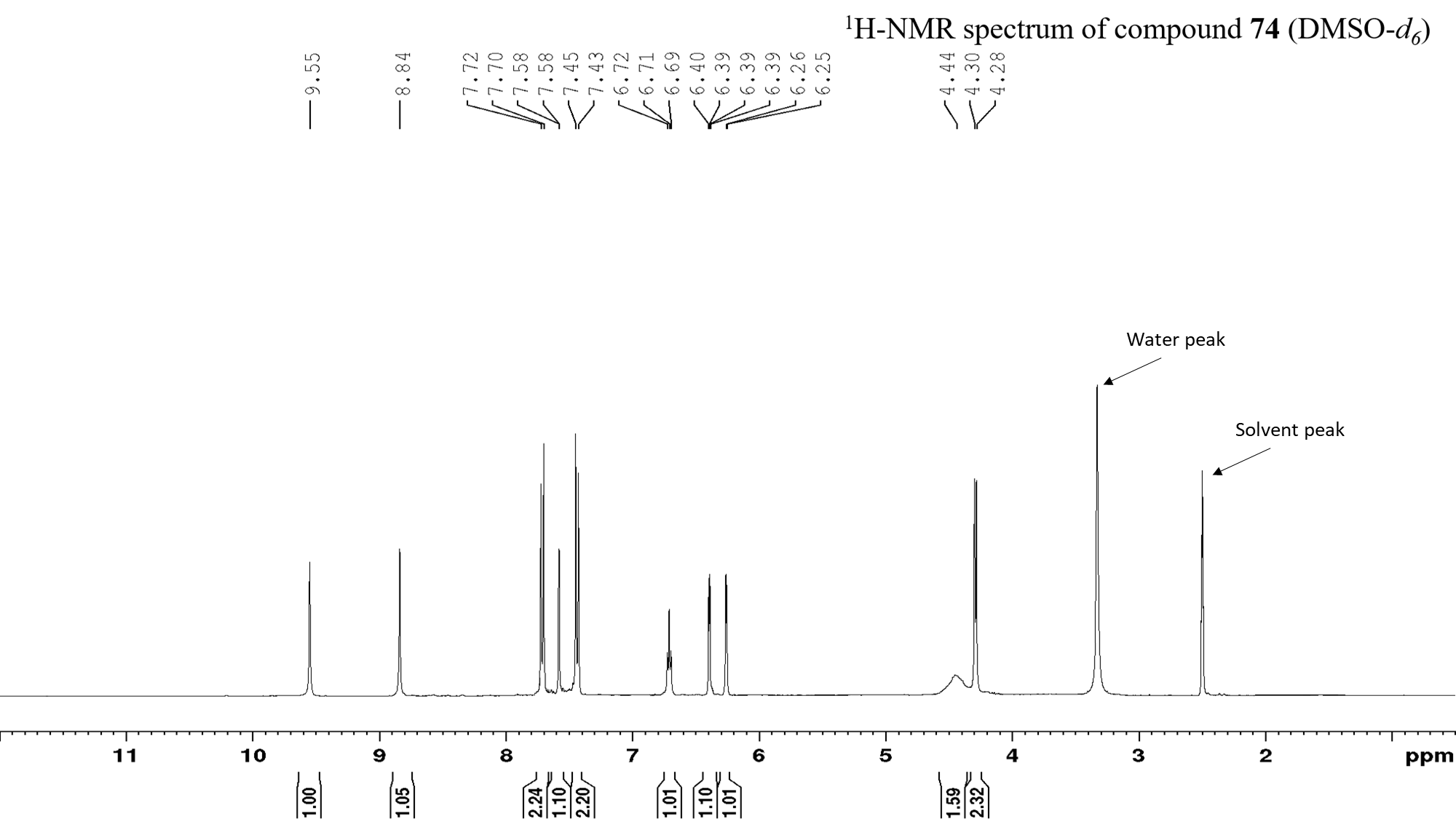


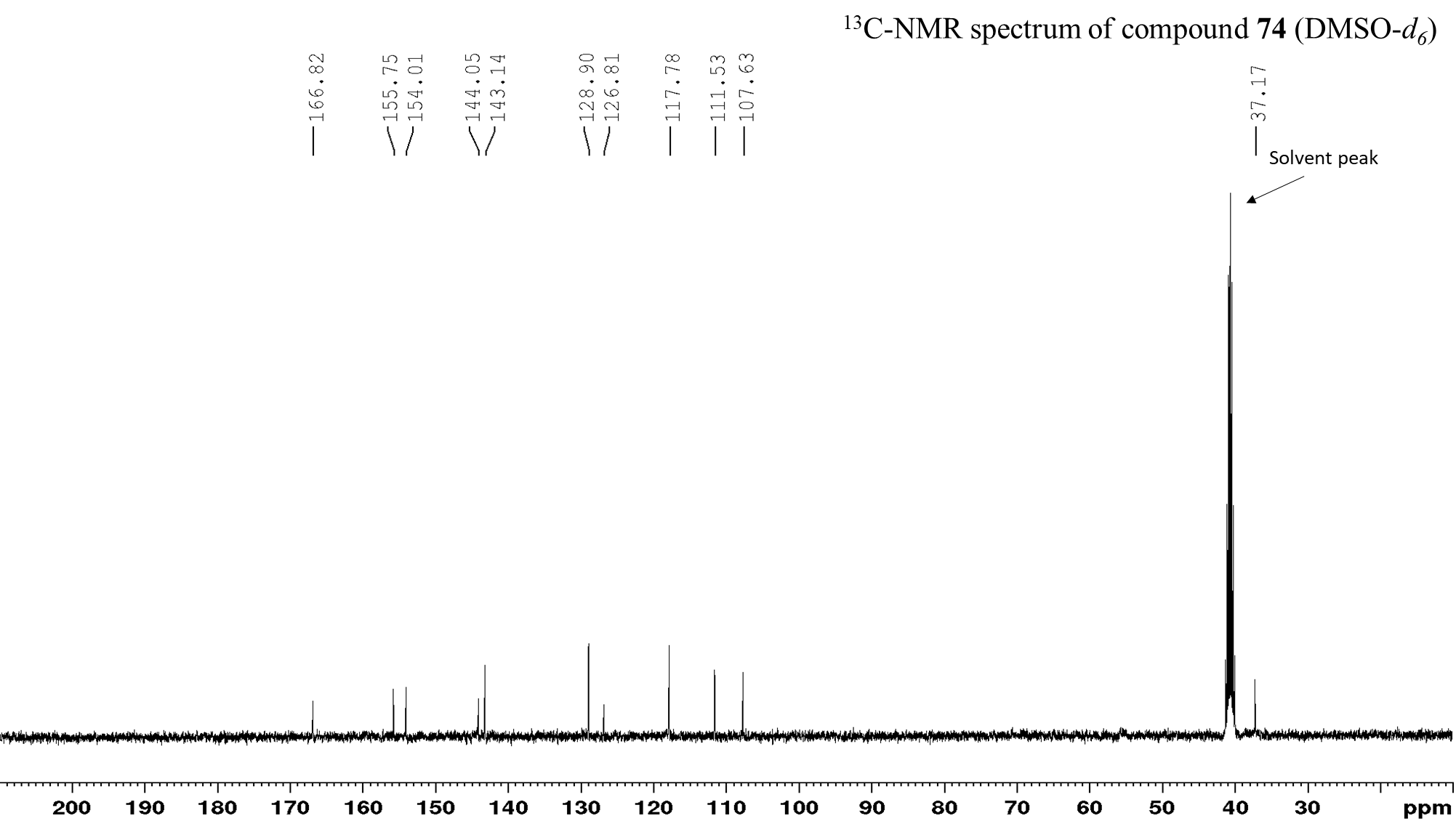


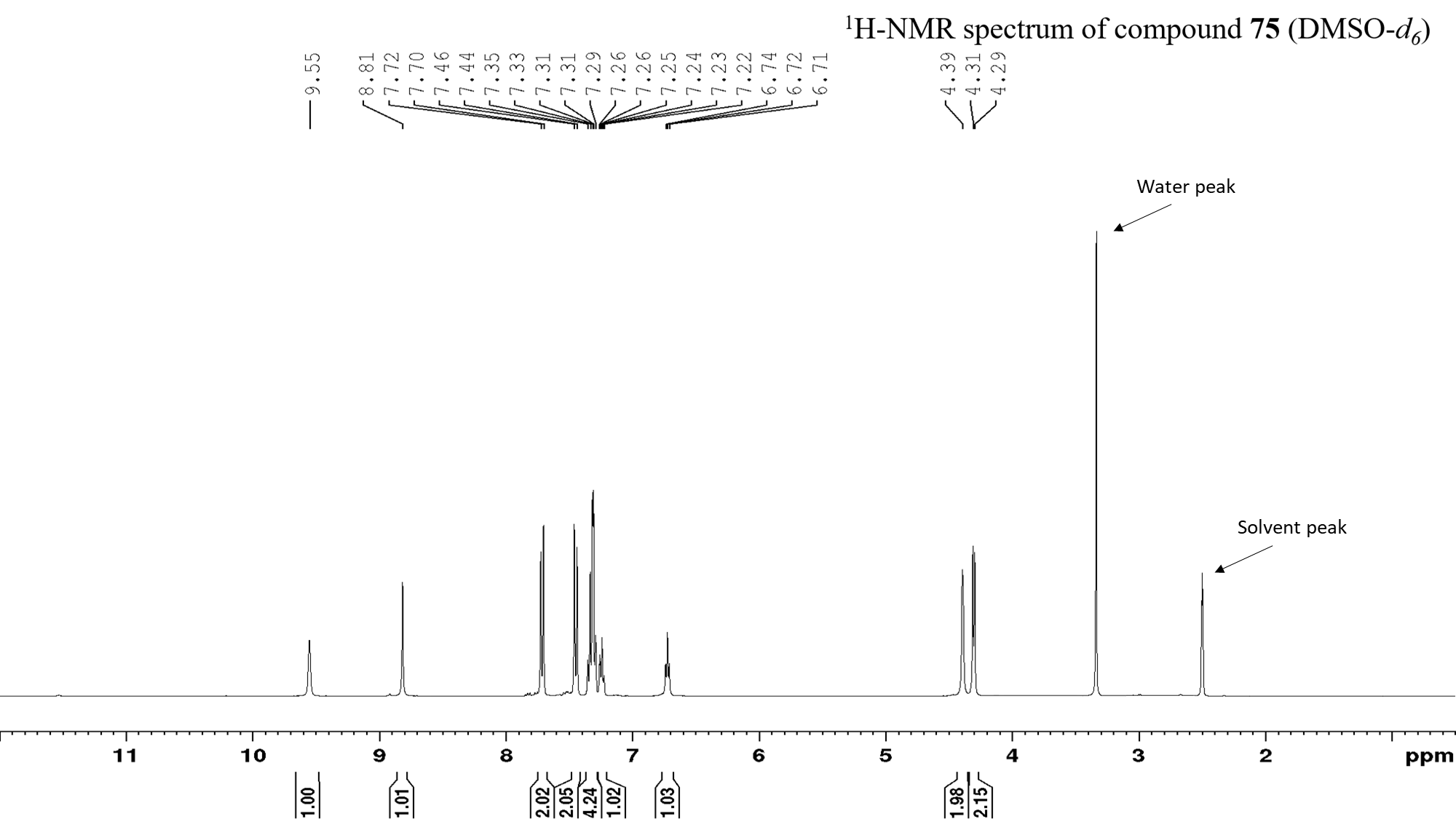


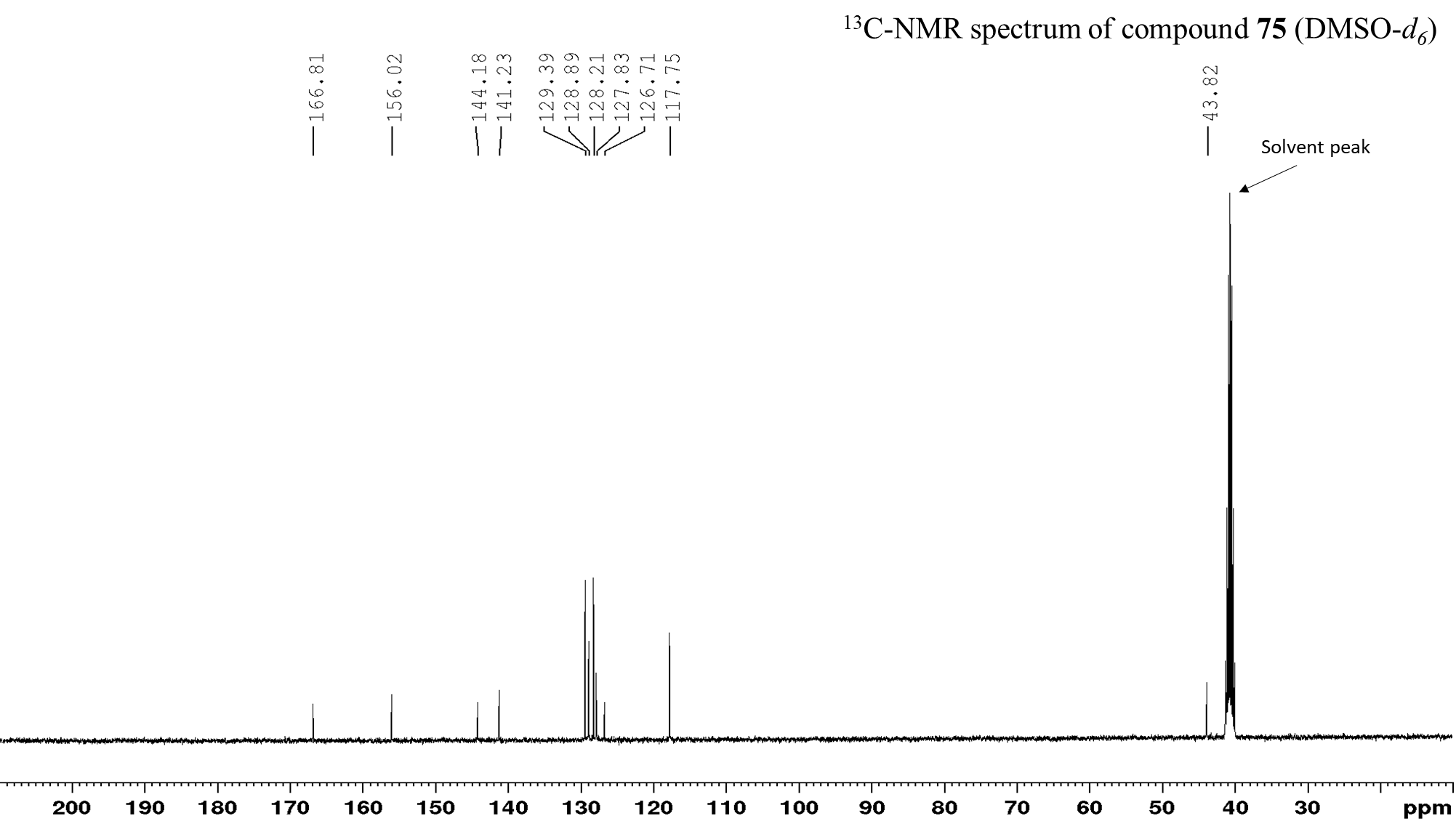


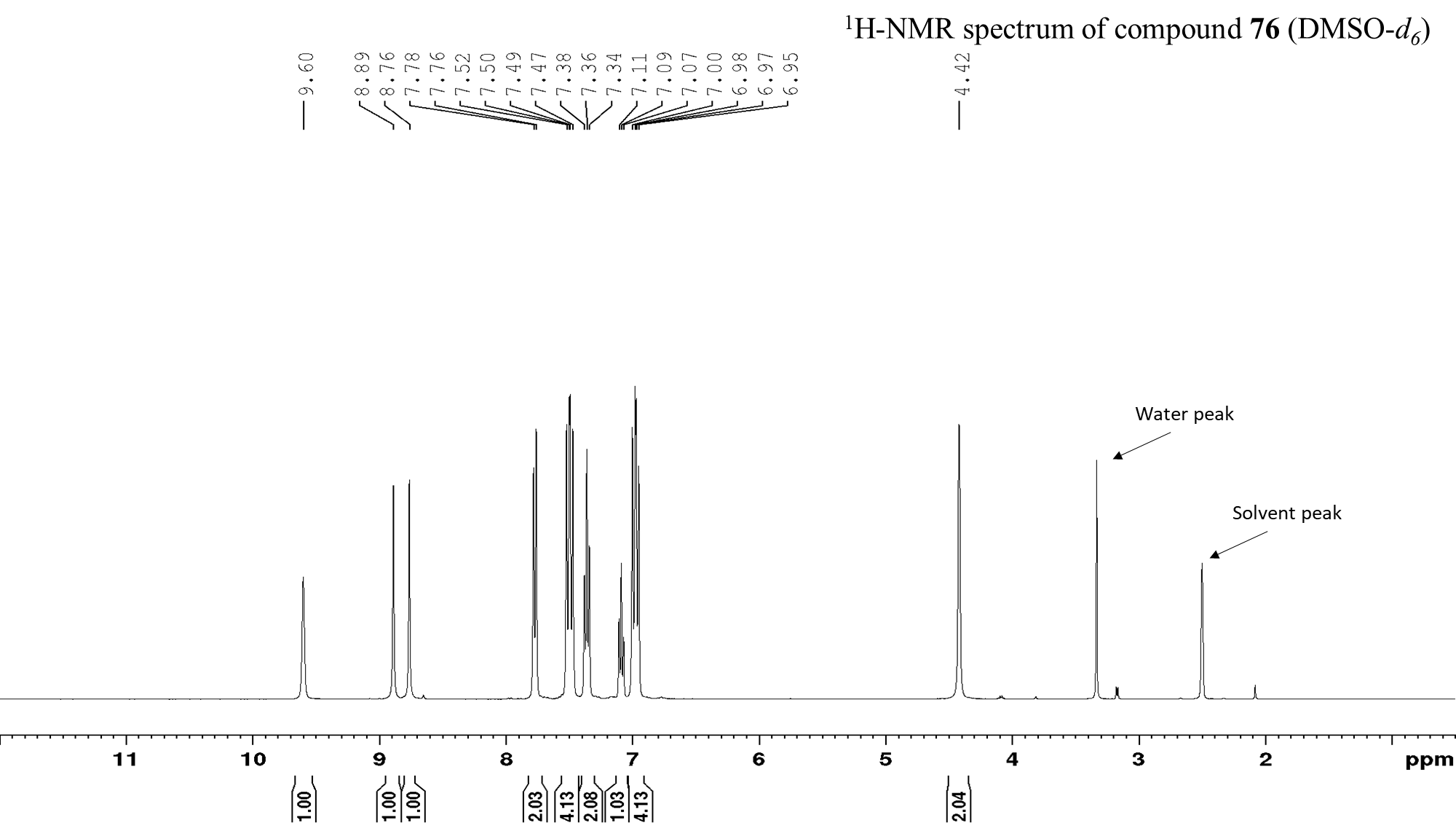


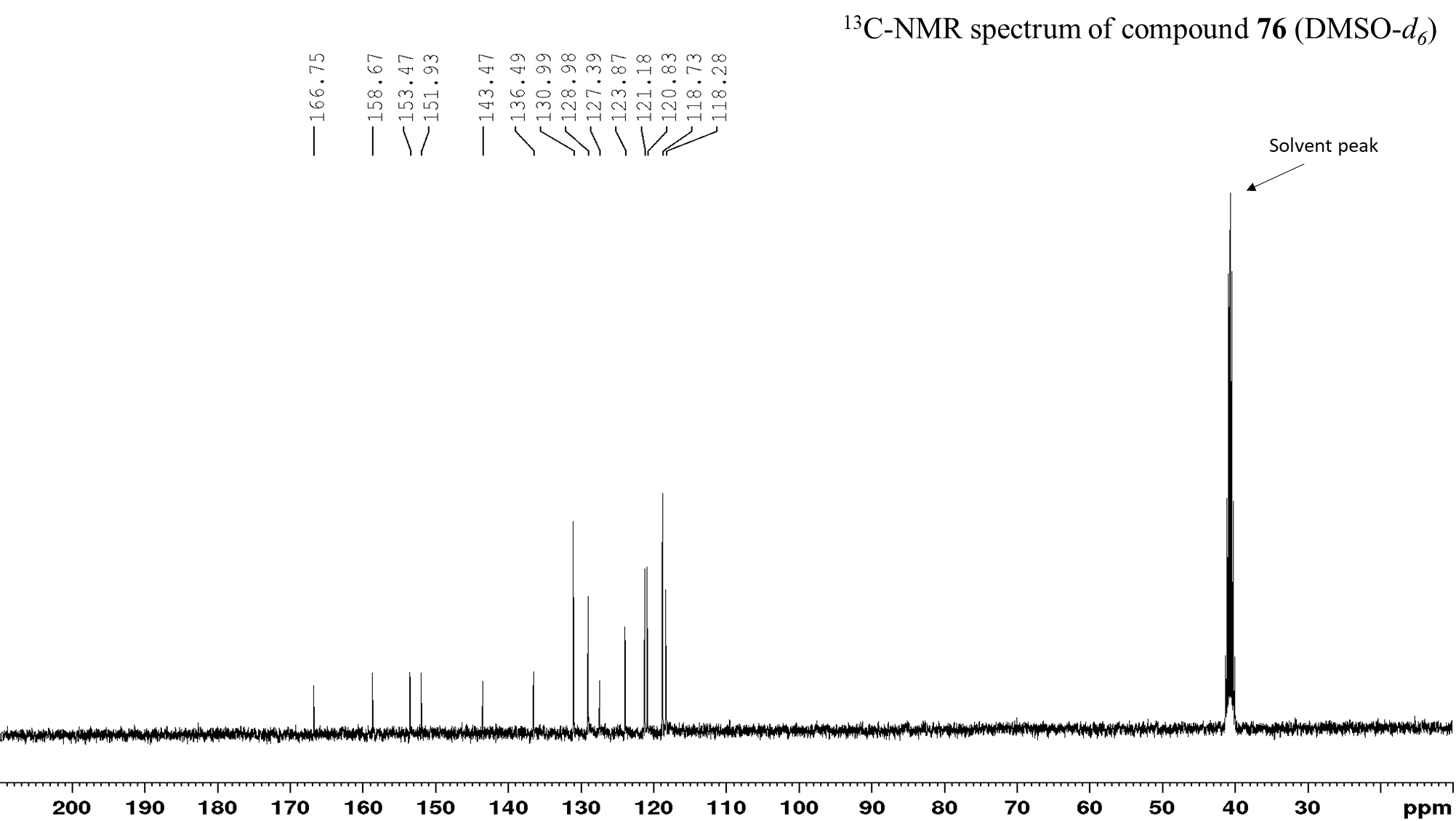


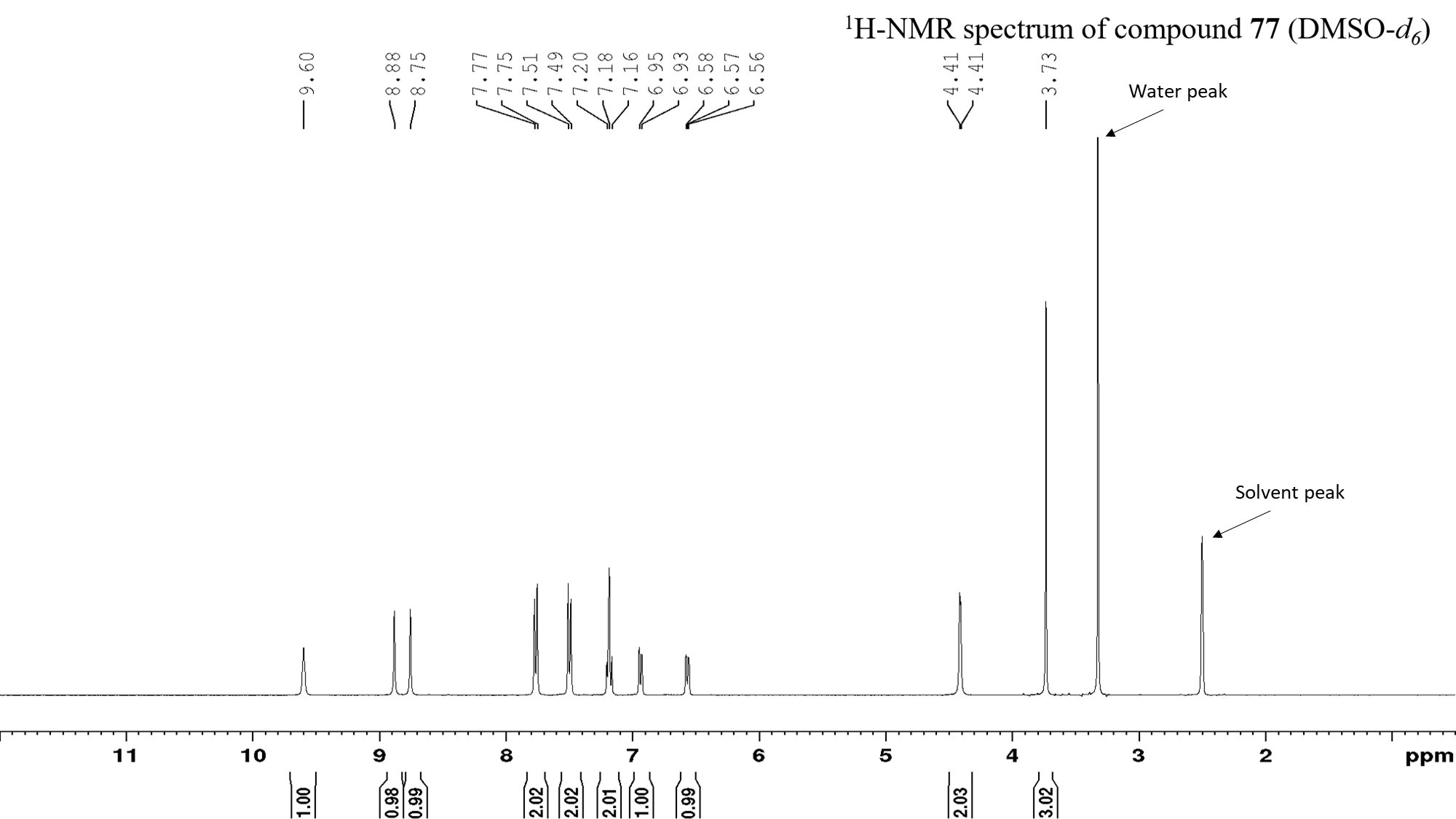


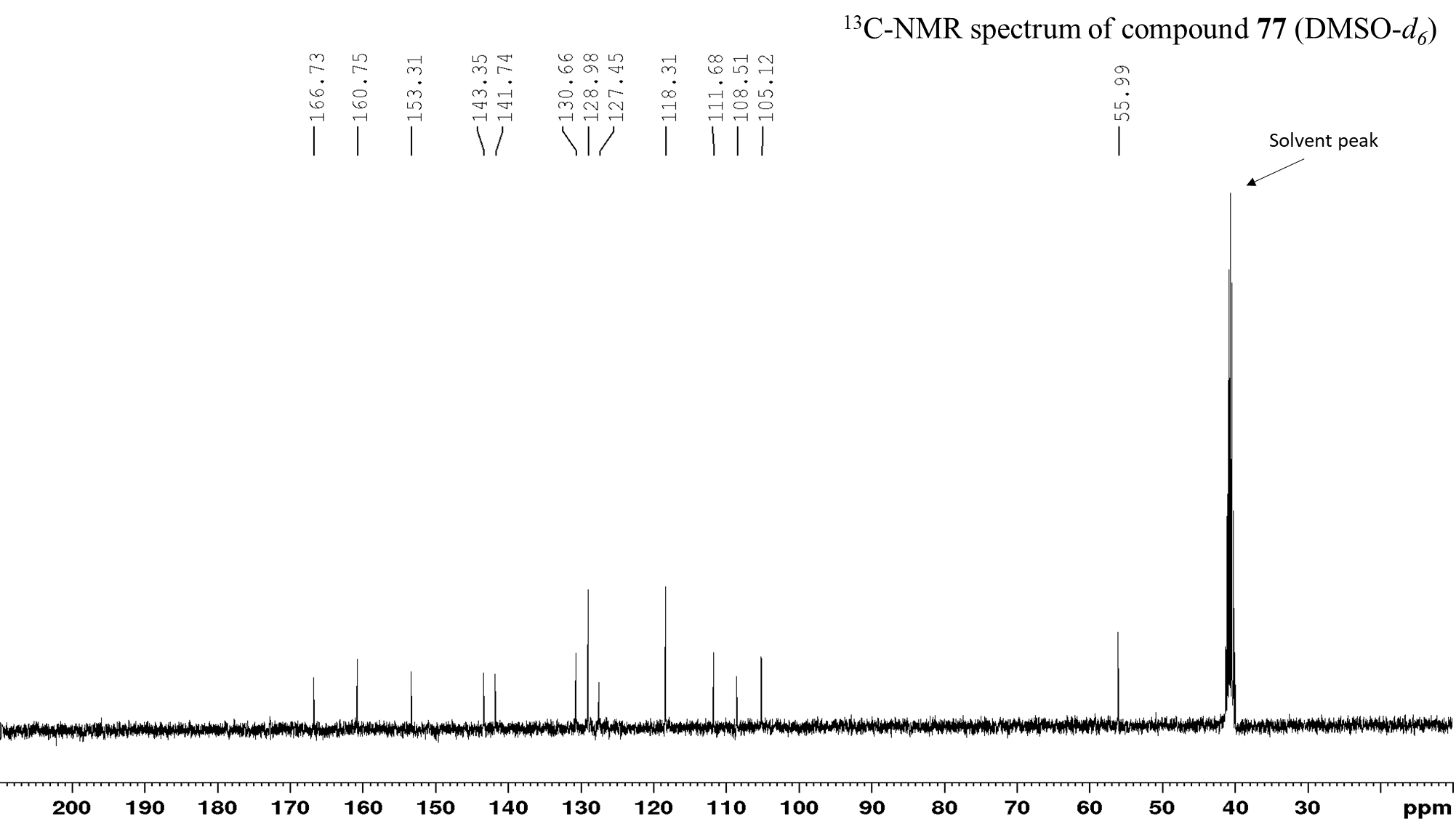


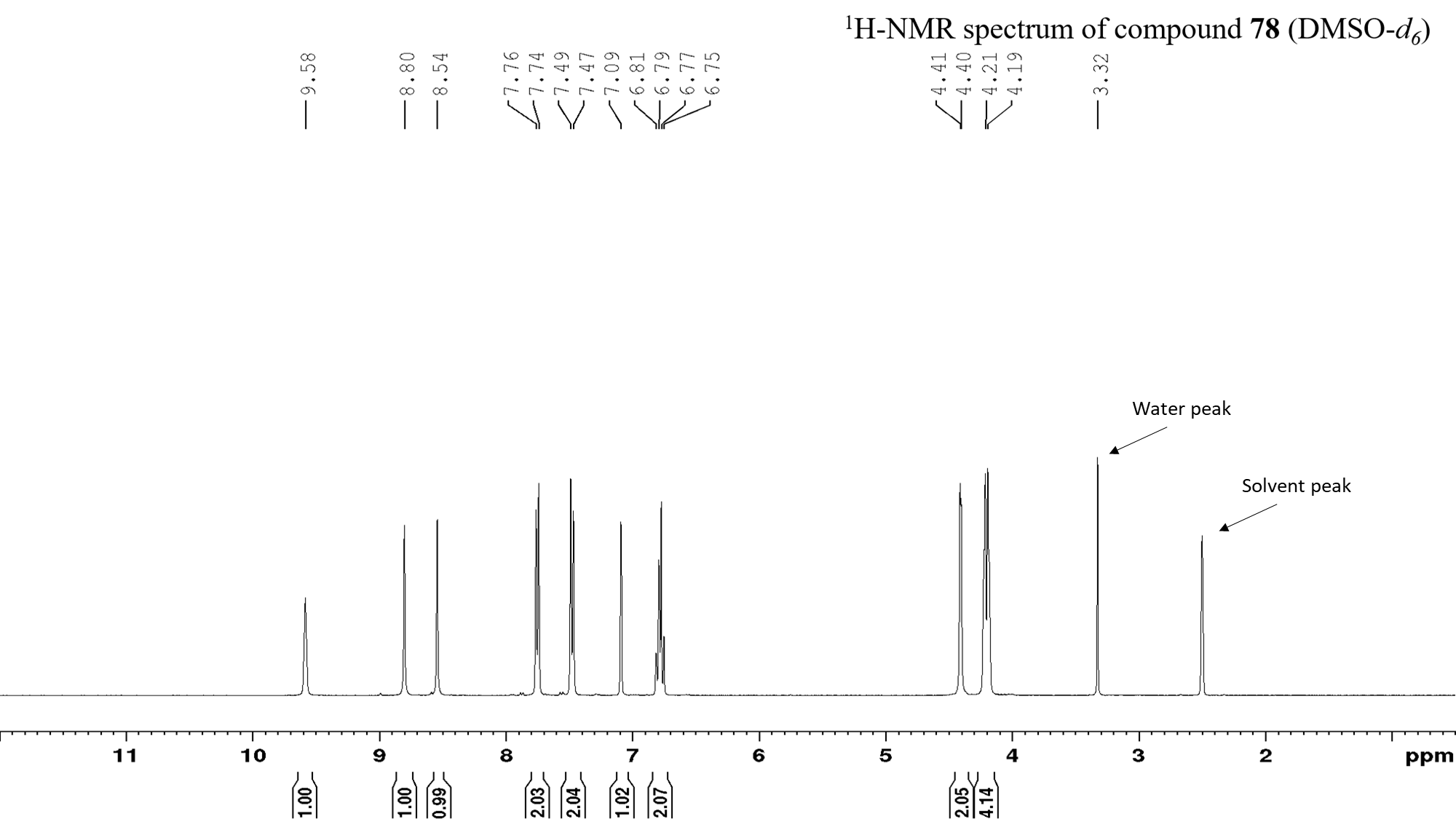


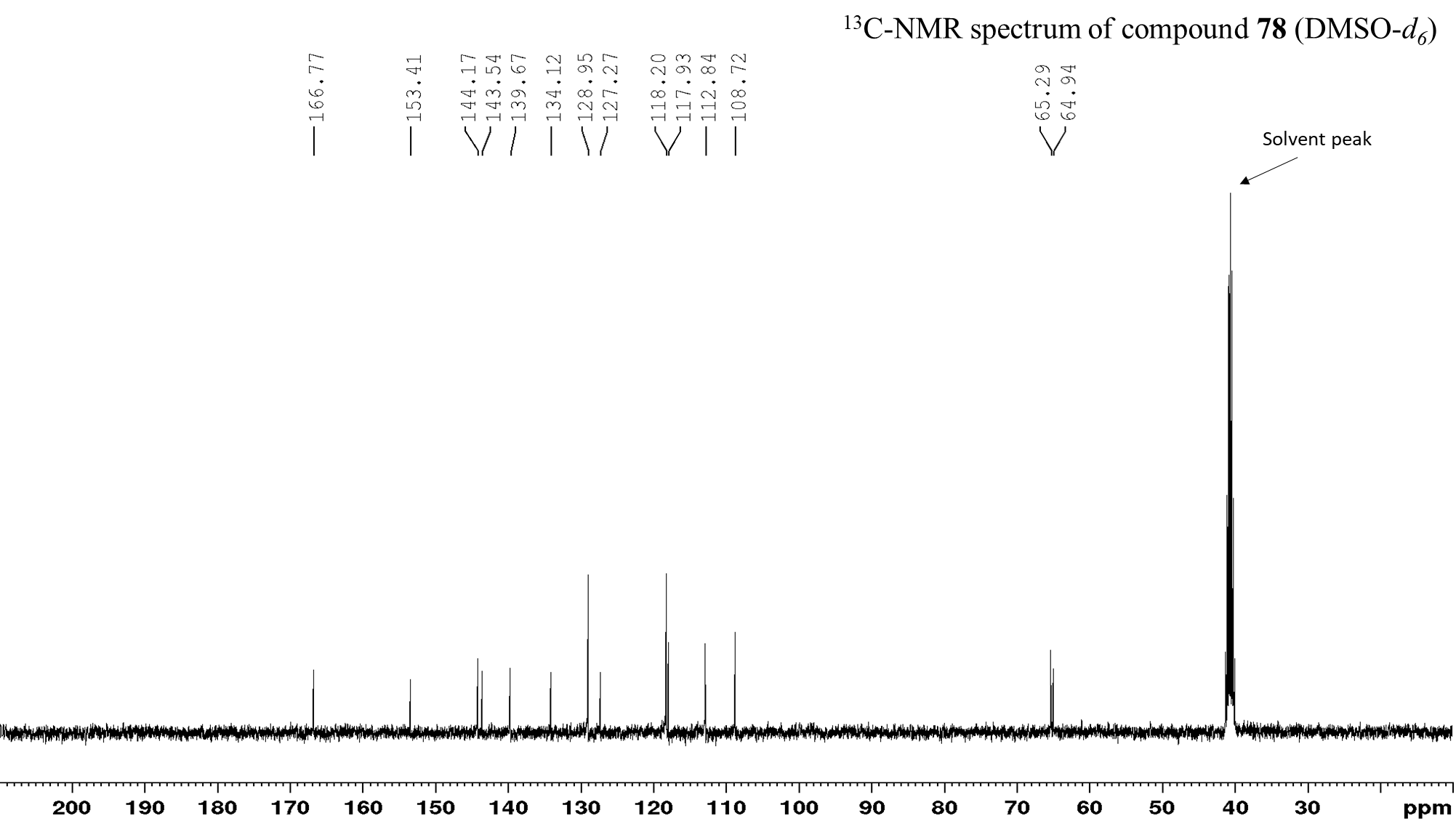


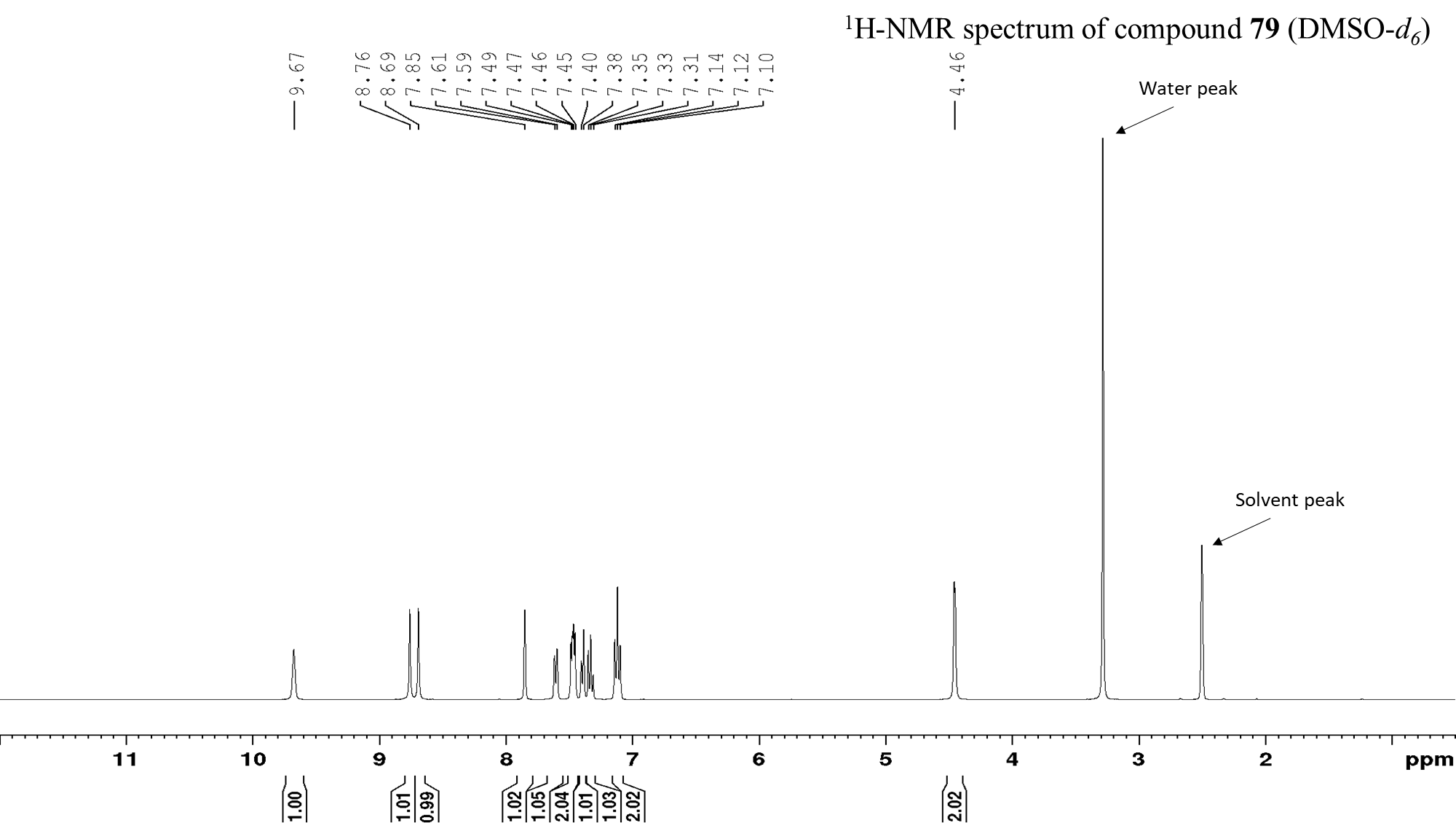


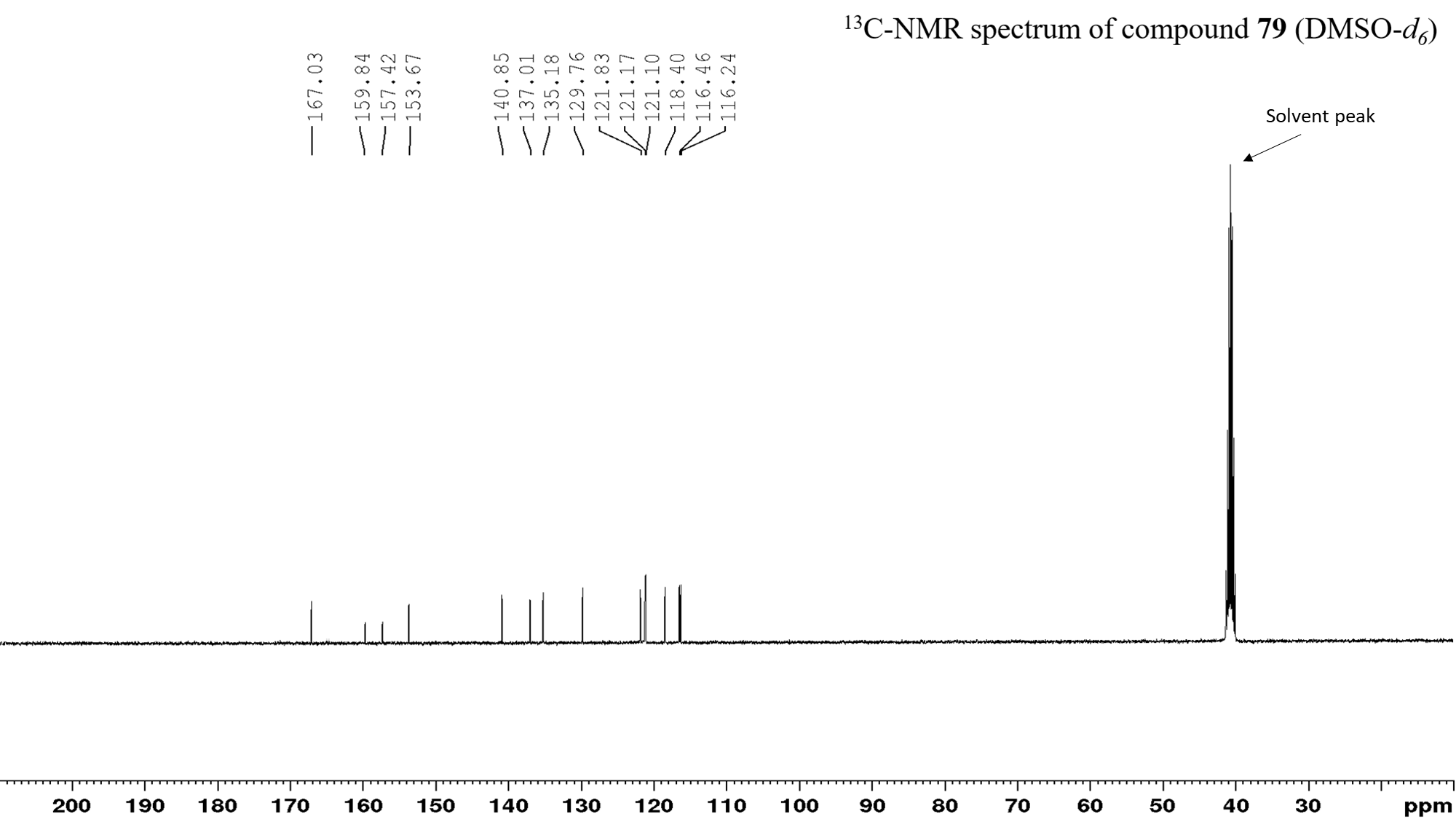


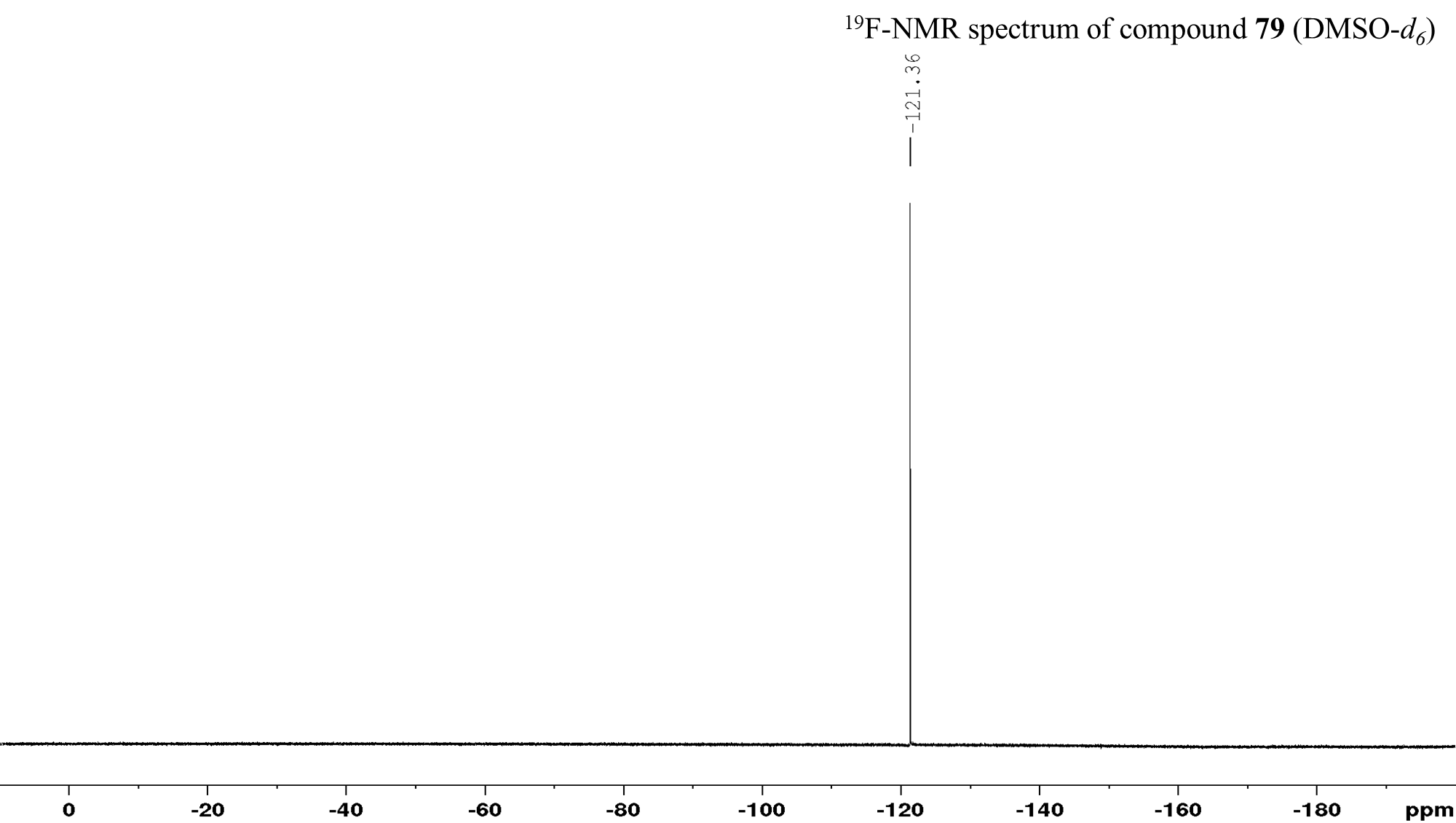


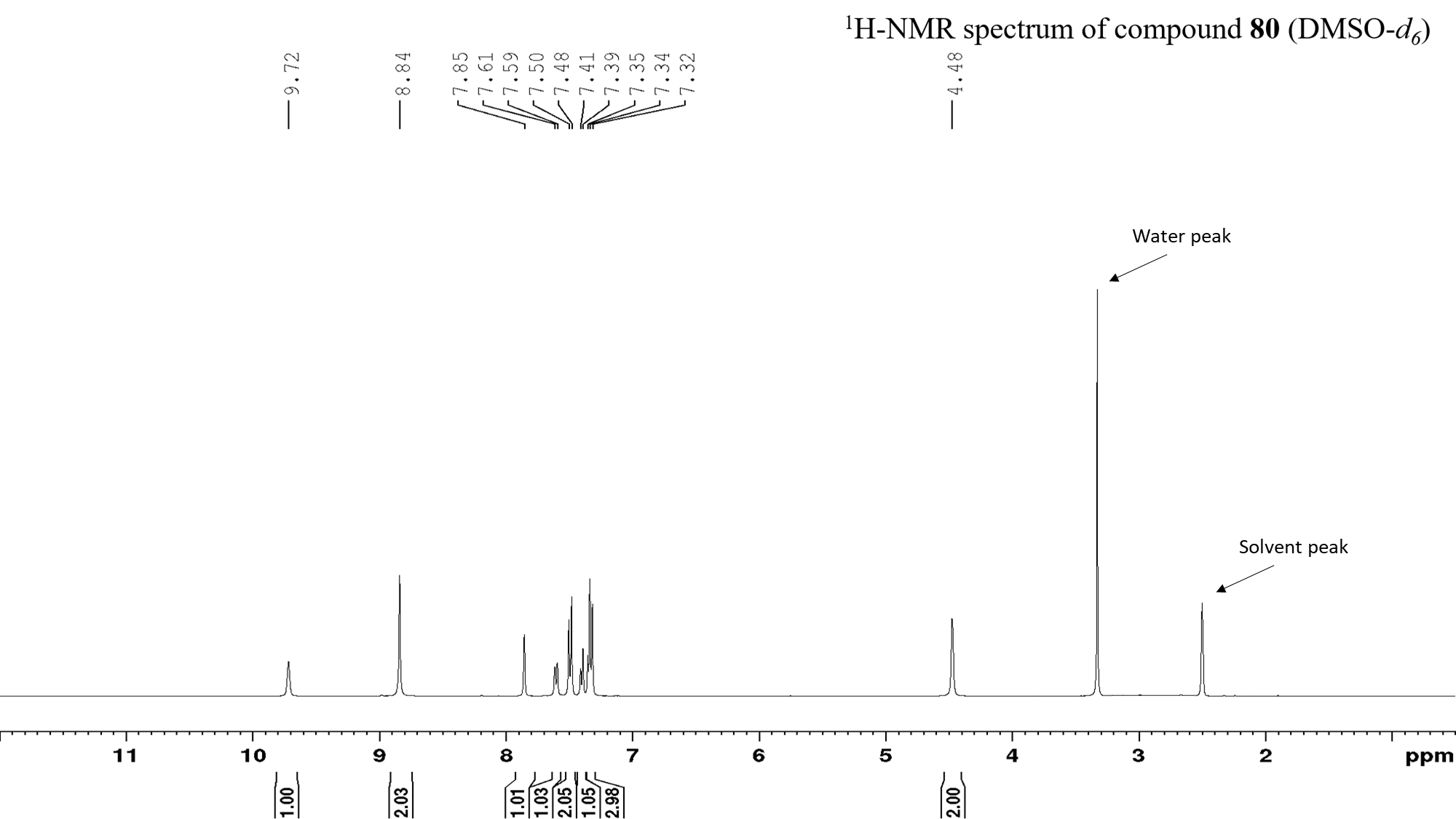


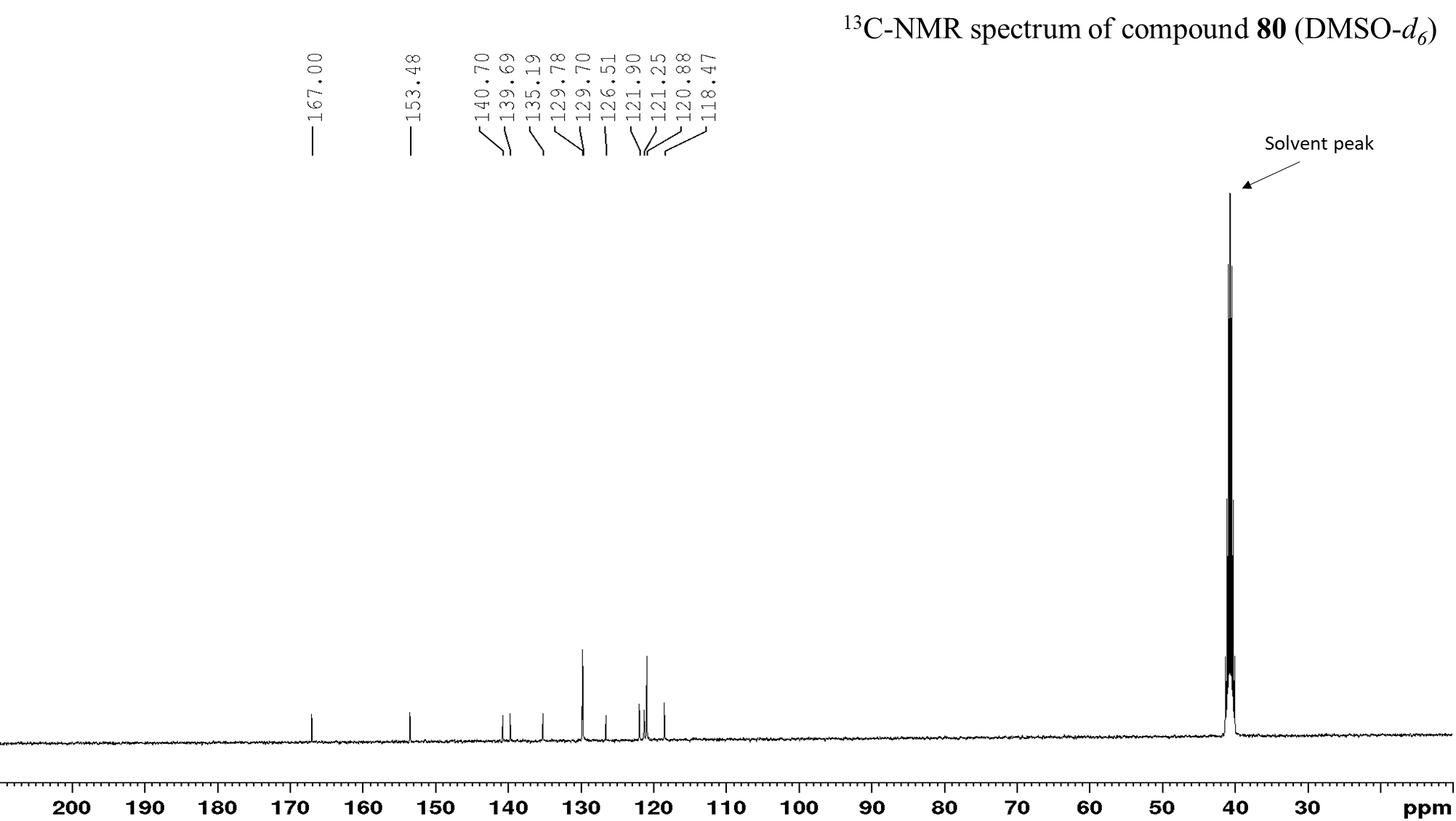


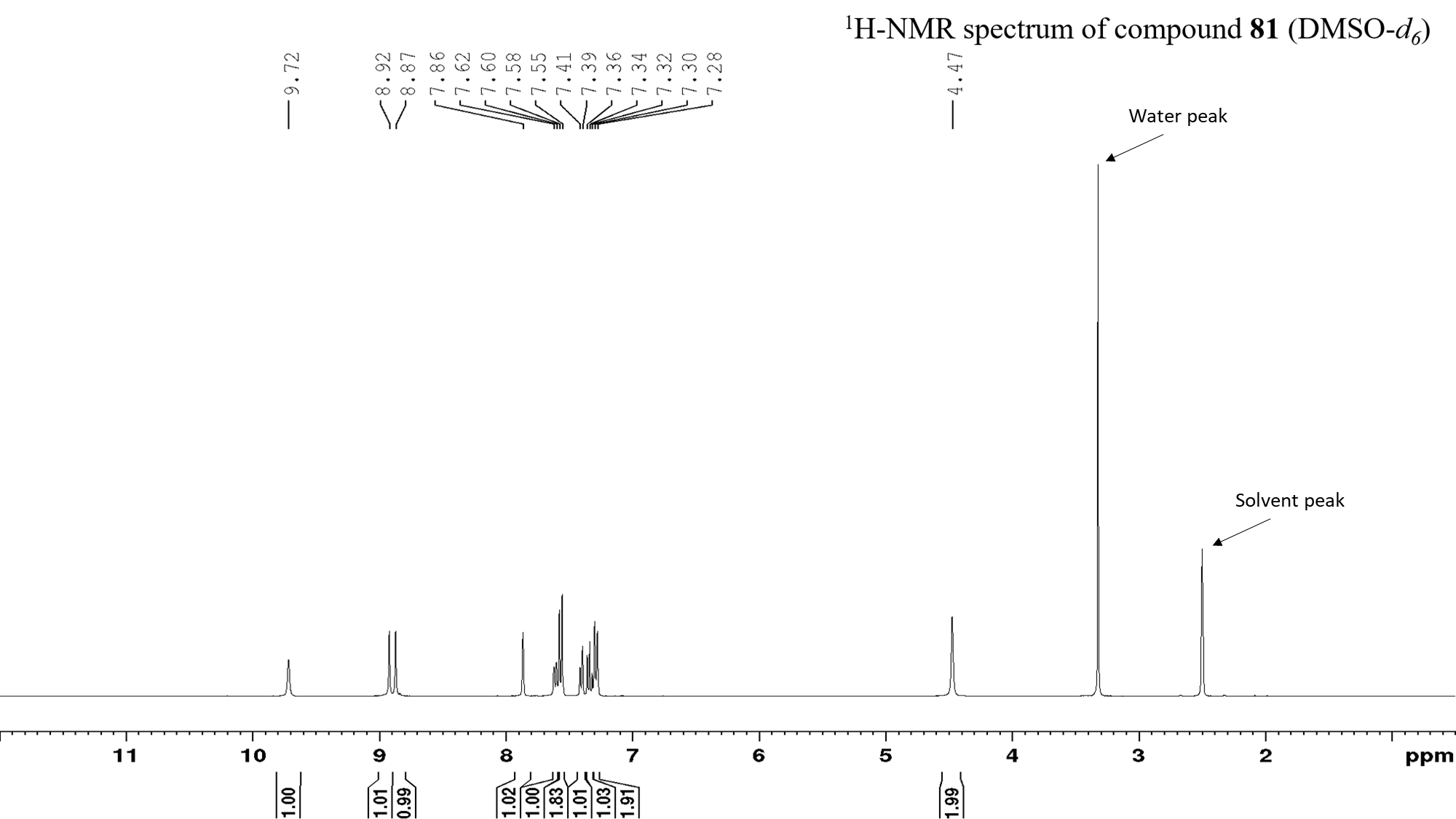


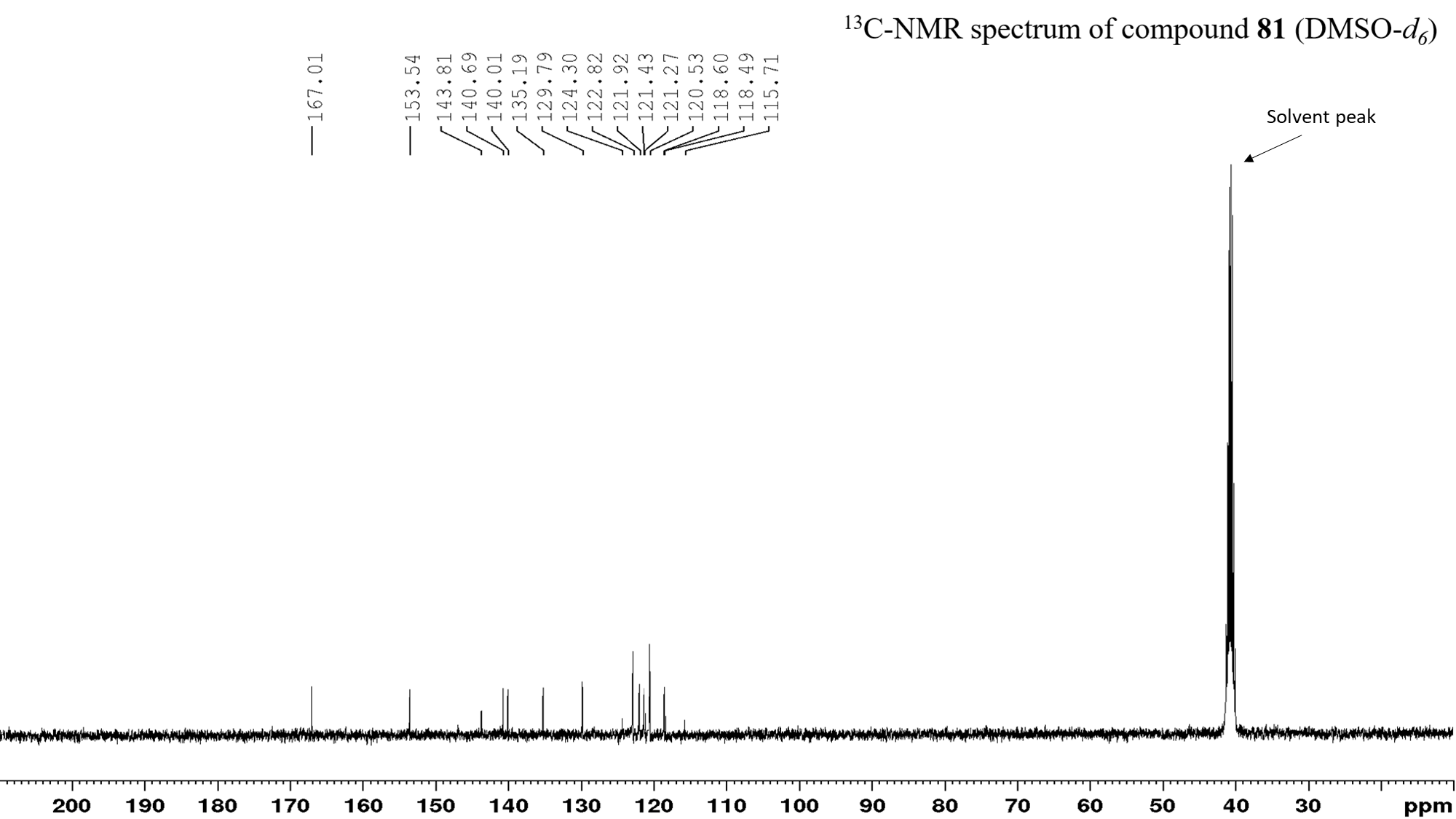


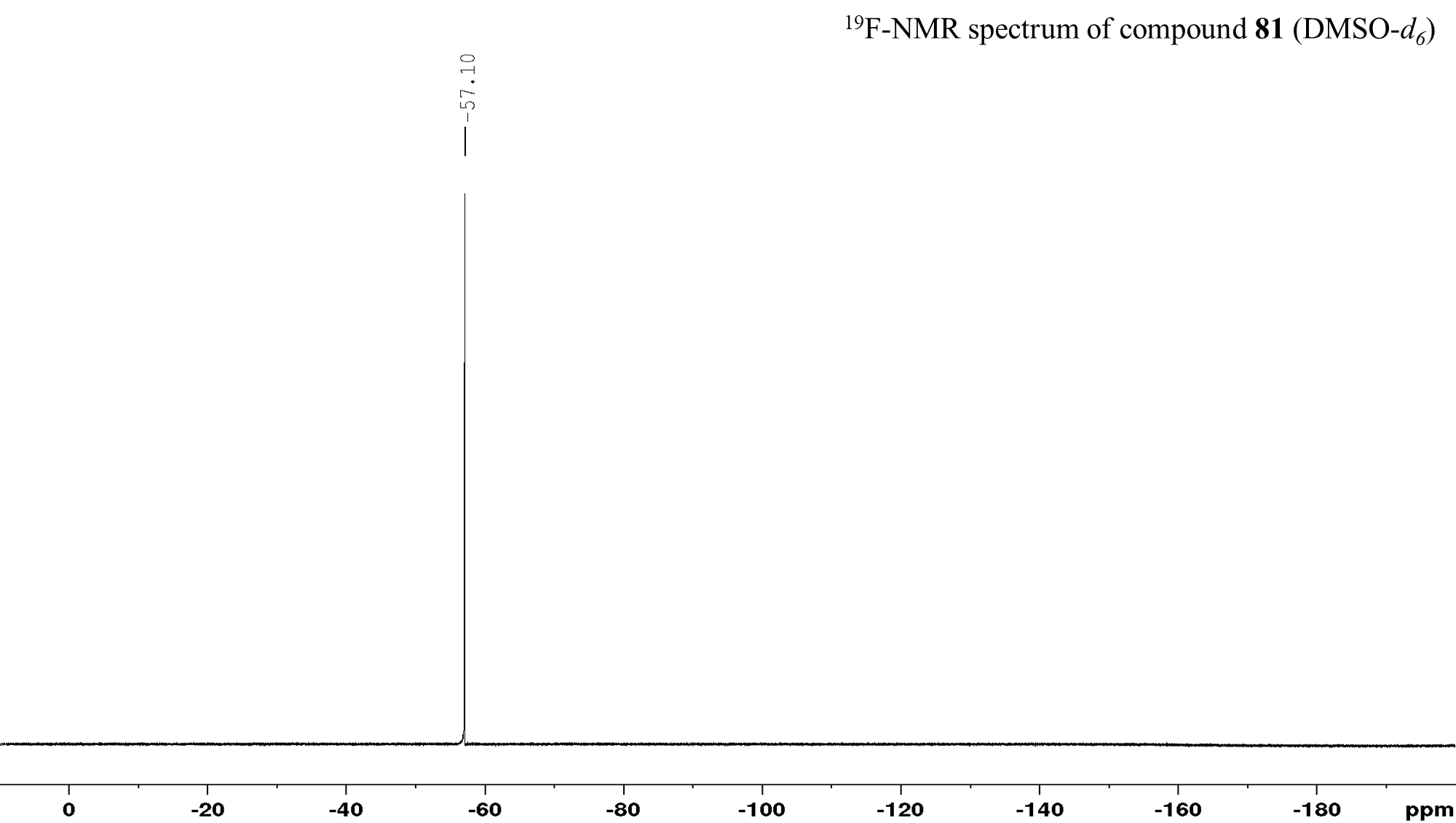


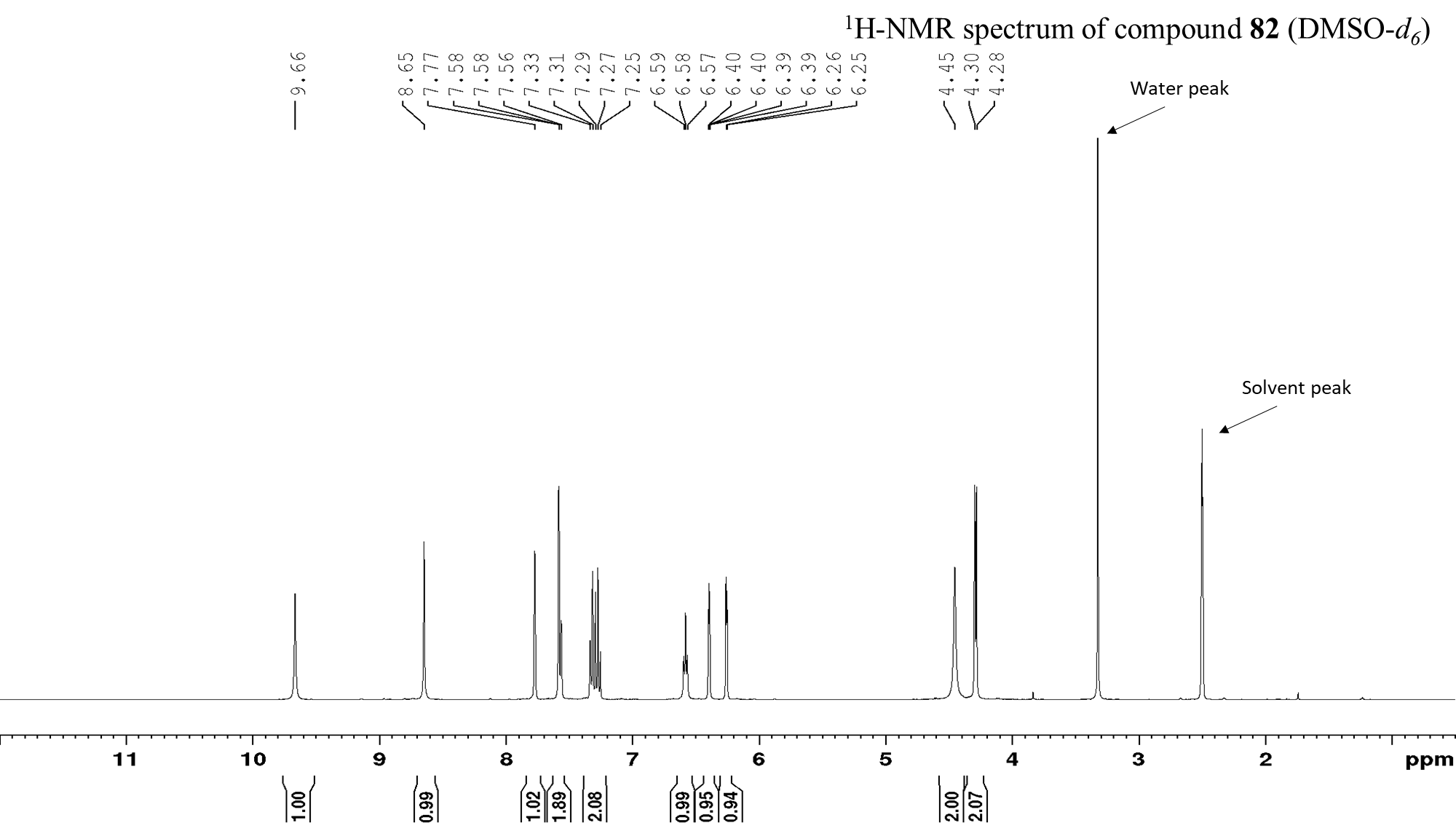


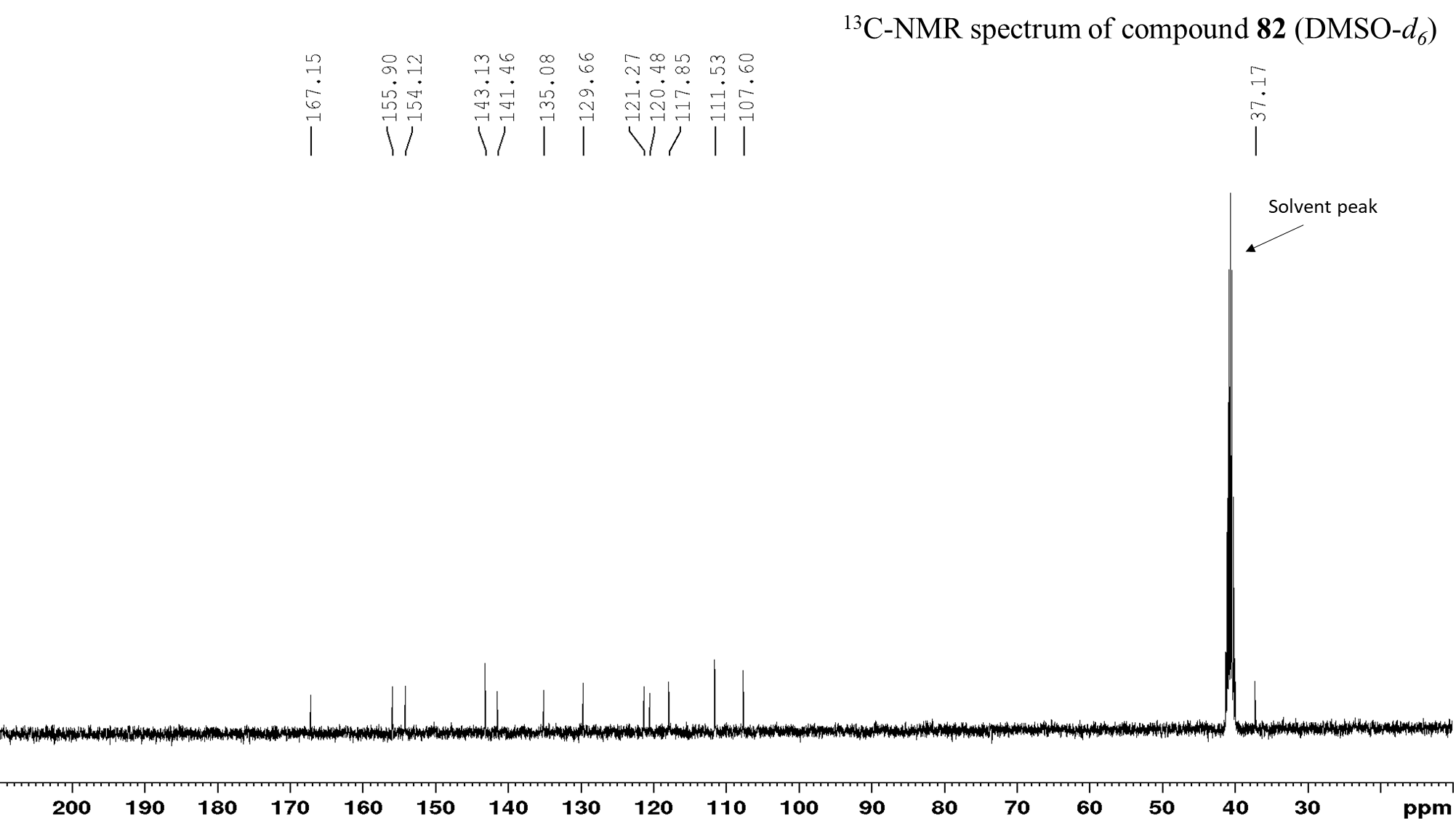


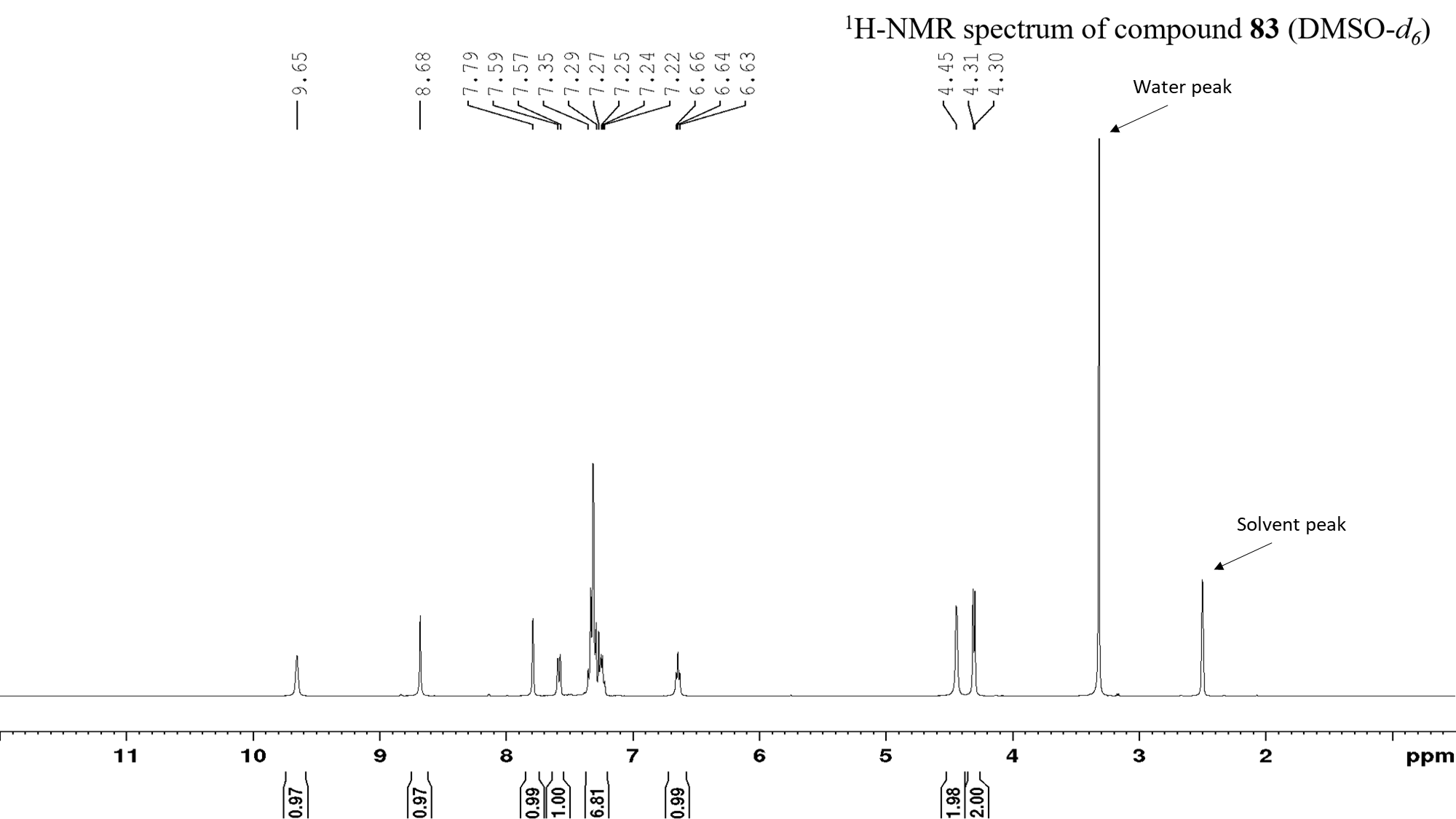


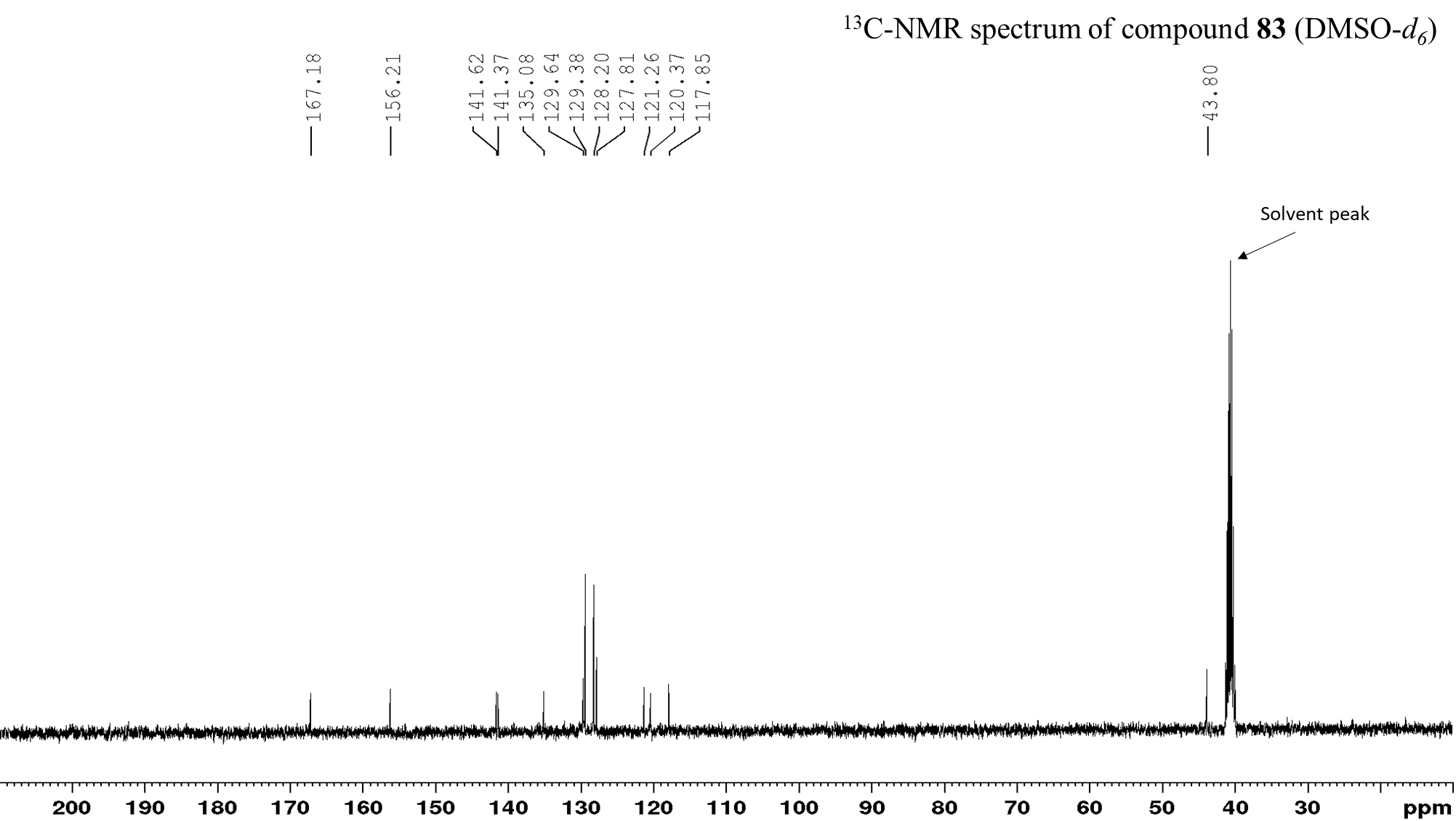


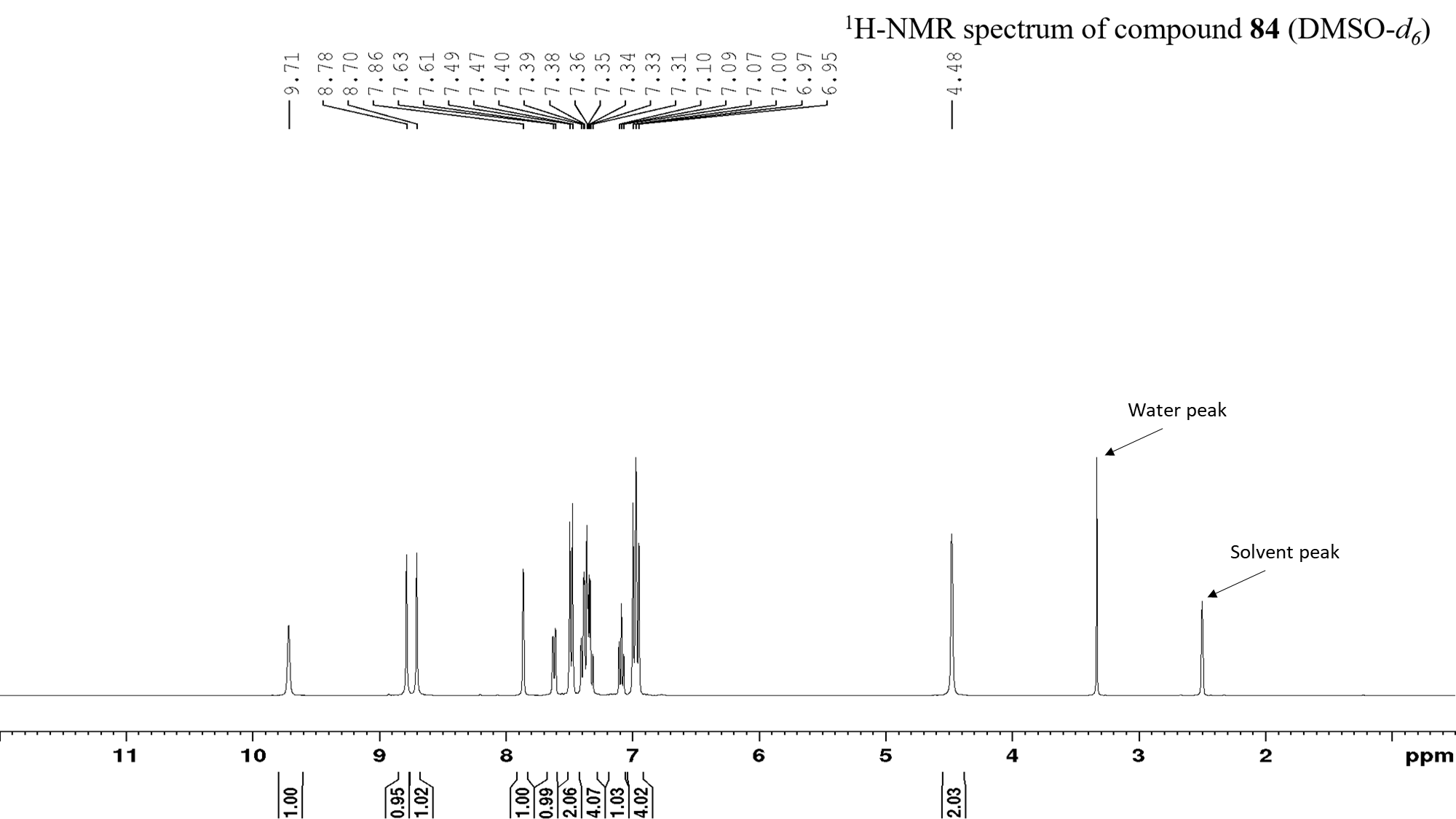


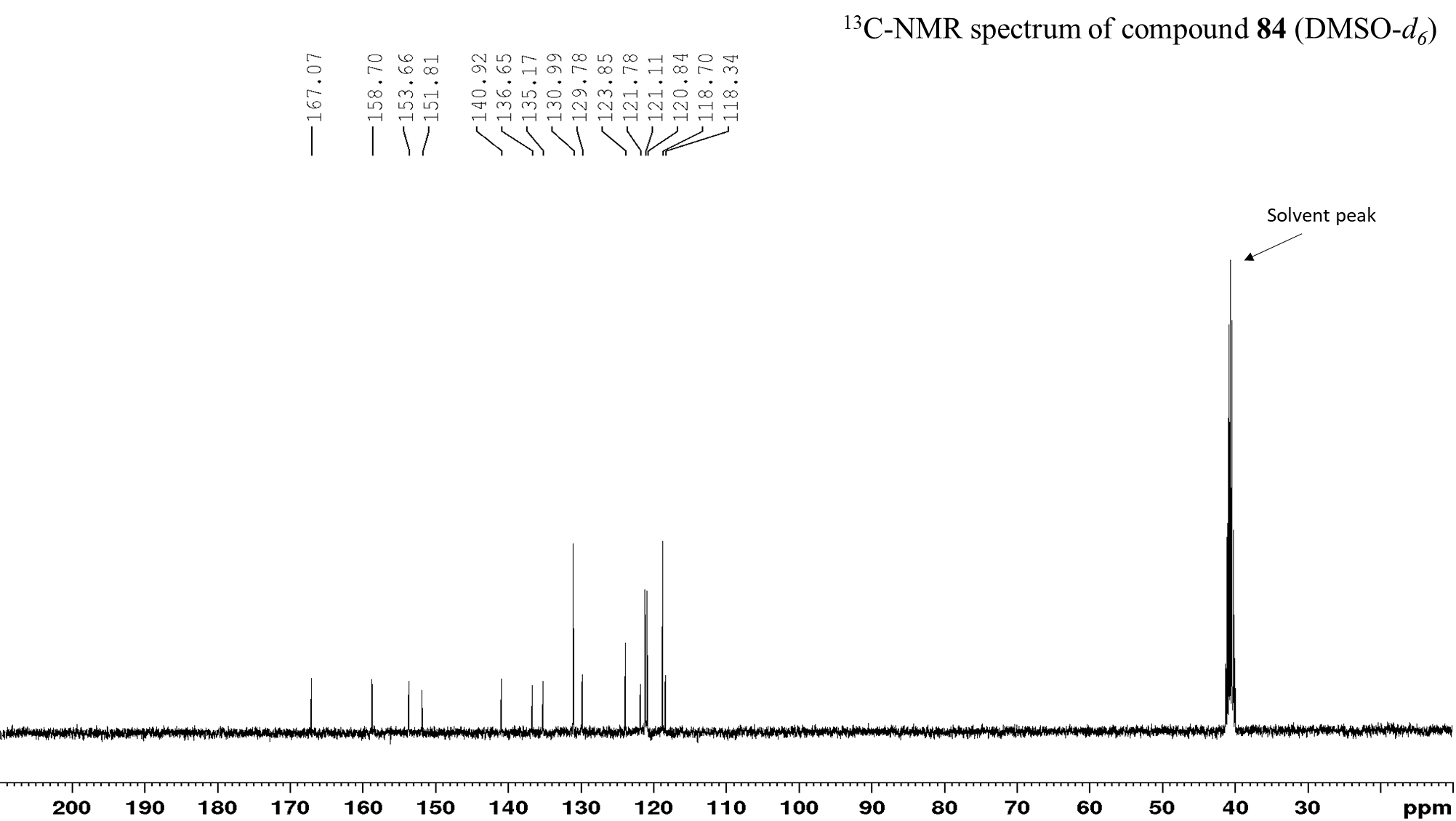


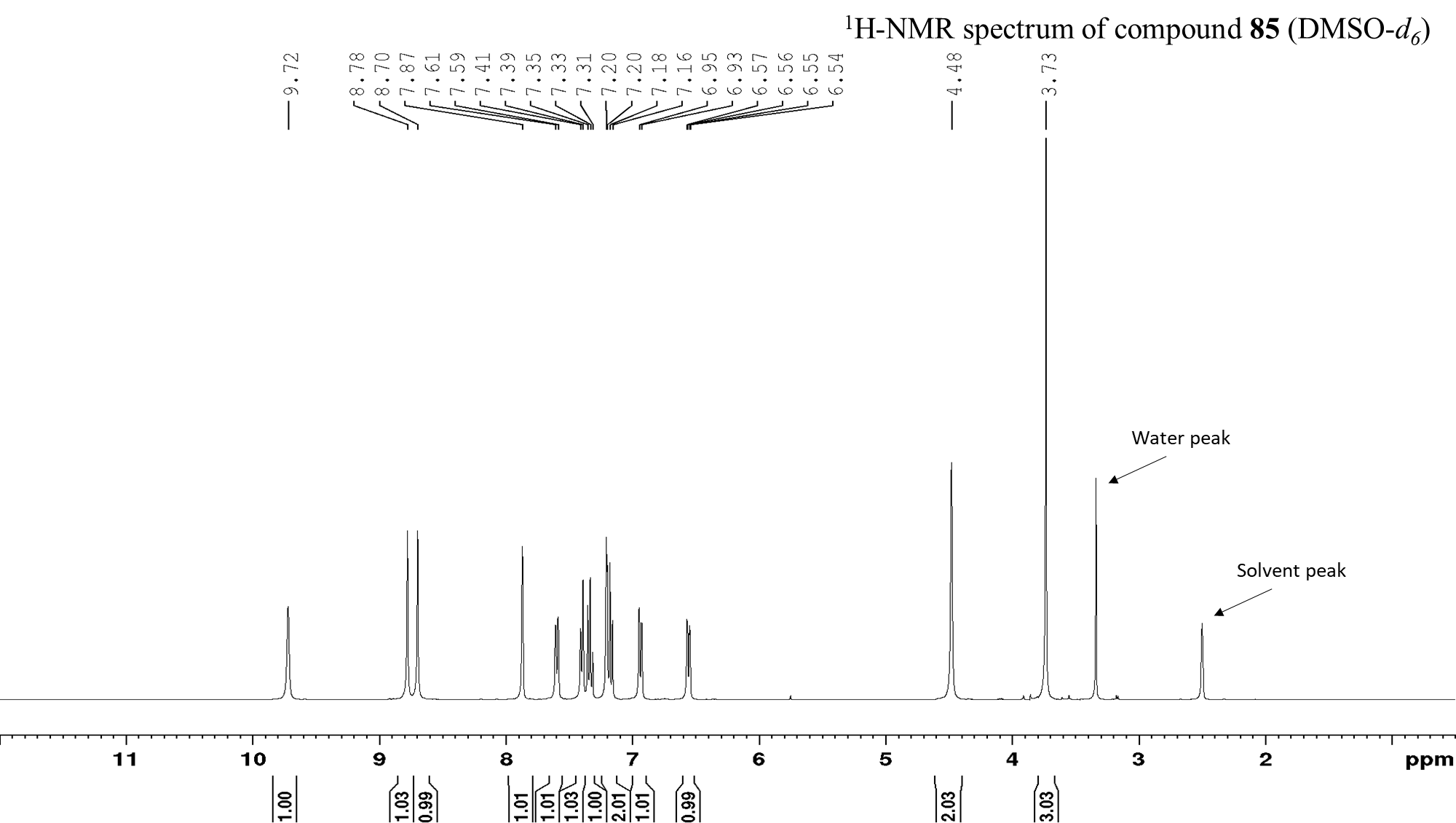


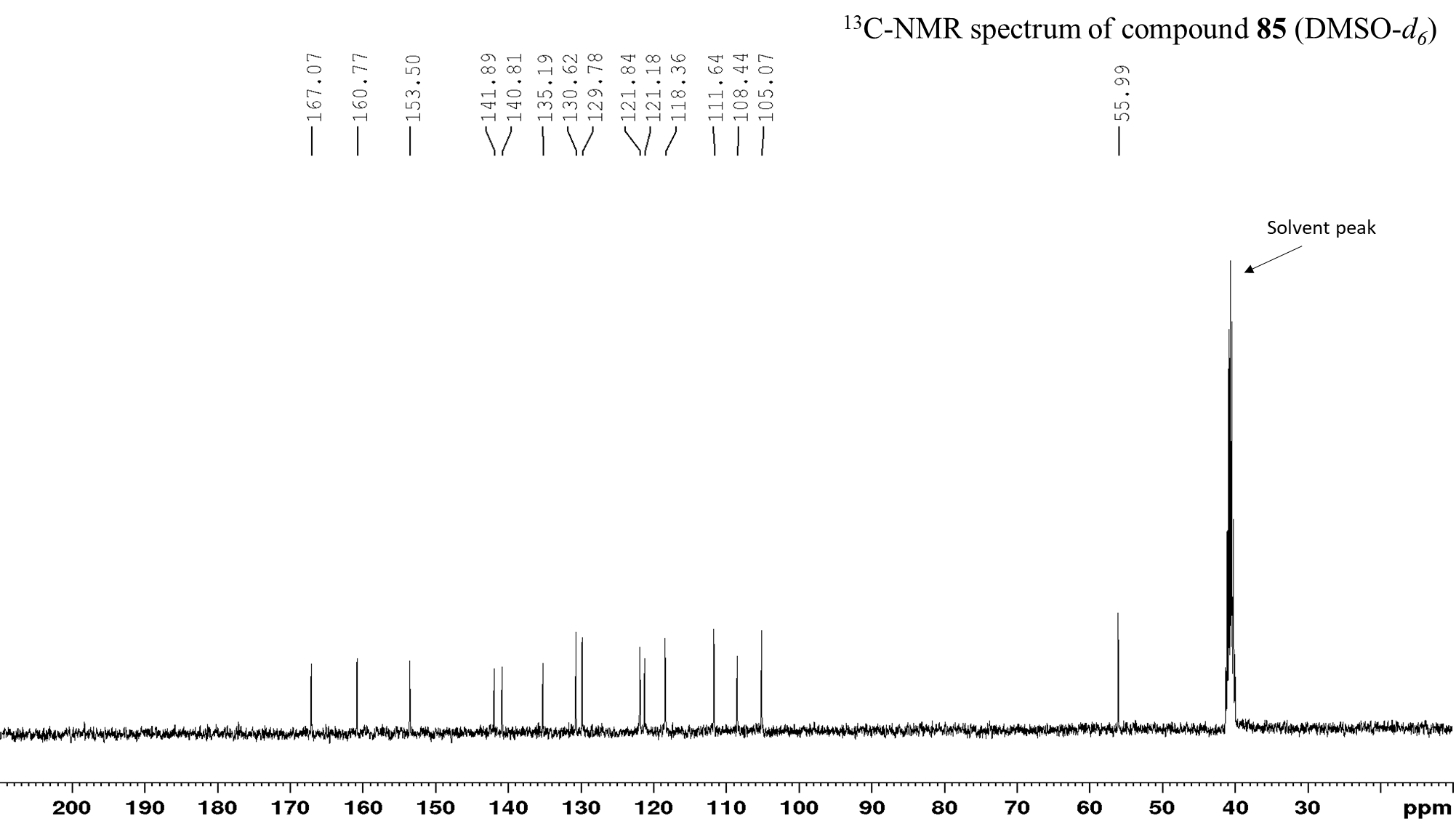


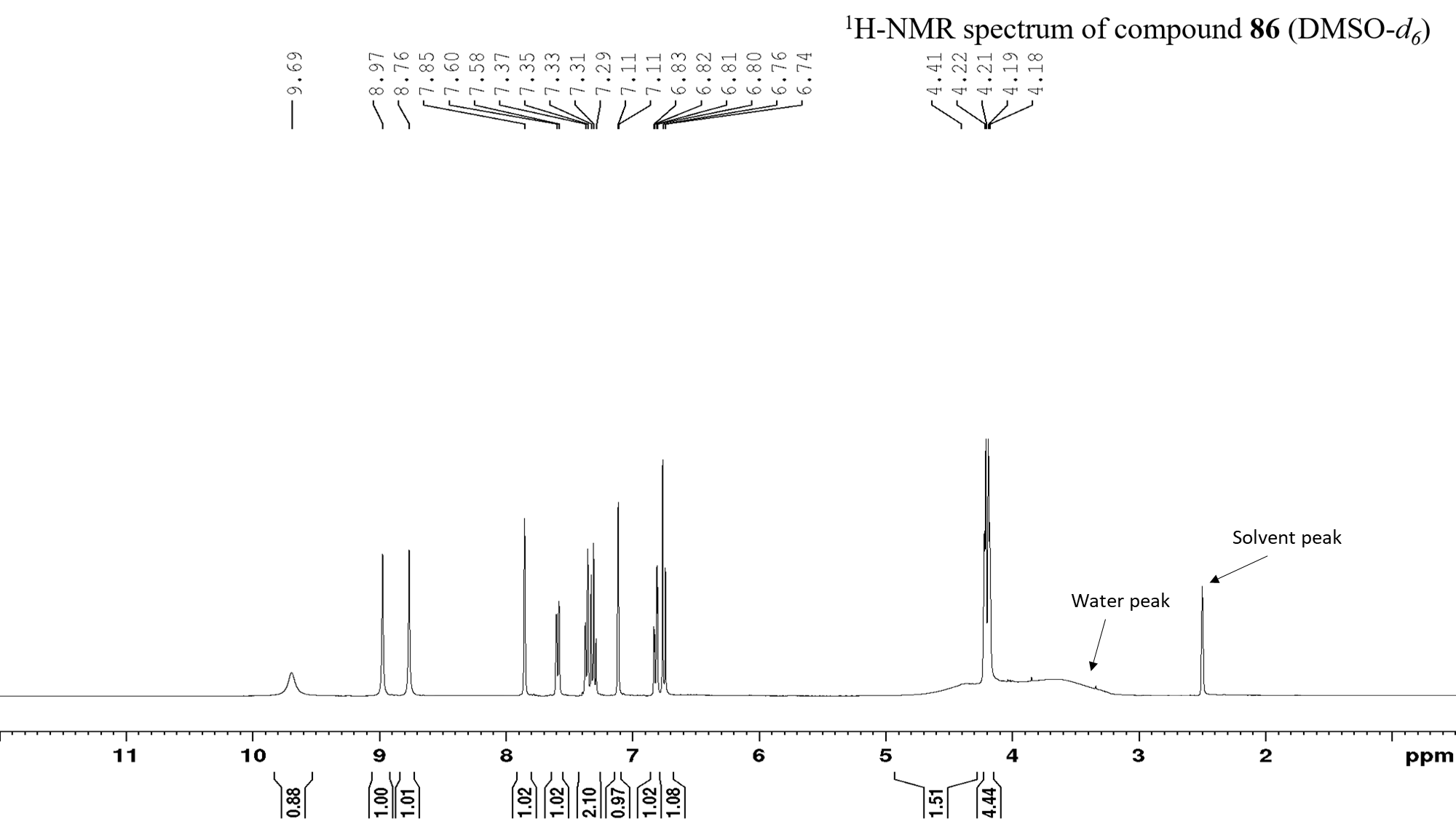


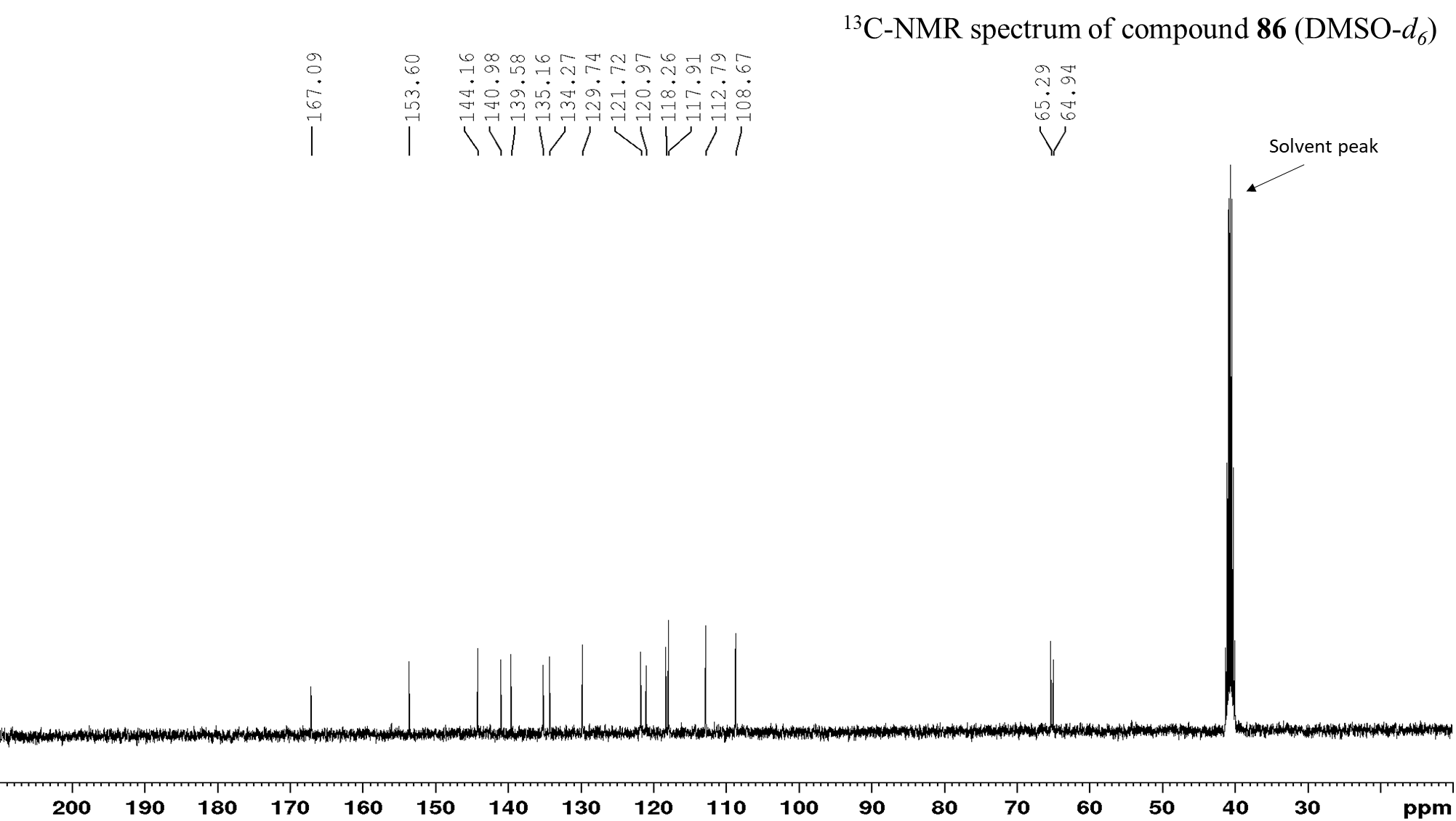

Supplement: Supplementary file 2 — Supporting information. [file ARDP-358-e202400963-s001.docx]
